# ZEB1 integrates stemness, differentiation blockade and immune suppression to orchestrate osteosarcoma aggressiveness

**DOI:** 10.1186/s13046-026-03757-9

**Published:** 2026-06-15

**Authors:** Anna Zaccaria, Caterina Cascini, Chiara Ratti, Laura Botti, Davide Pernici, Valeria Cancila, Elisabetta Armiraglio, Antonina Parafioriti, Cristina Meazza, Sabina Sangaletti, Claudio Tripodo, Katia Scotlandi, Mario P. Colombo, Daniele Lecis, Claudia Chiodoni

**Affiliations:** 1 Department of Experimental Oncology, Molecular Immunology Unit, Fondazione IRCCS Istituto Nazionale dei Tumori, Via Venezian 1, Milan, 20133 Italy; 2 Department of Health Sciences, Tumor Immunology Unit, University of Palermo School of Medicine, Palermo, 90134 Italy; 3 Department of Pathology, Istituto Ortopedico Gaetano Pini, Milan, 20122 Italy; 4 Pediatric Oncology Unit, Fondazione IRCCS Istituto Nazionale dei Tumori, Milan, 20133 Italy; 5 Advanced Pathology Laboratory, IFOM ETS-The AIRC Institute of Molecular Oncology, Milan, 20139 Italy; 6 Laboratory of Experimental Oncology, IRCCS Istituto Ortopedico Rizzoli, Bologna, 40136 Italy

**Keywords:** Osteosarcoma, ZEB1, Stemness, Cellular differentiation, Immune suppression

## Abstract

**Background:**

Osteosarcoma (OS) is a highly aggressive bone malignancy primarily affecting children and adolescents, with limited therapeutic options. The transcription factor ZEB1, a key regulator of epithelial-mesenchymal transition (EMT), promotes cancer stemness and tumor progression.

**Methods:**

We investigated ZEB1 role in OS using genetic knockout and silencing in murine and patient-derived cellular OS models. Using in vitro functional assays, we assessed the effect of ZEB1 down-modulation on stemness (sarcosphere formation), anchorage-independent growth, chemotherapy sensitivity, and osteoblastic differentiation (osteogenic markers and matrix mineralization), while gene expression profiling provided hints on ZEB1 regulation of tumor cell transcriptional program. In vivo studies evaluated tumor growth and lung metastasis in ZEB1-deficient models, while ZEB1 restoration experiments mechanistically proved its role in OS aggressiveness.

**Results:**

ZEB1 loss in OS mouse models markedly reduced stemness-associated gene expression and functional sarcosphere formation, impaired anchorage-independent growth, and increased sensitivity to chemotherapy. Furthermore, ZEB1 inhibition restored osteoblastic differentiation capacity, evidenced by upregulation of osteogenic markers and matrix mineralization.

In vivo, ZEB1 deletion in mouse OS cells significantly hindered tumor growth and lung metastasis, accompanied by increased tumor differentiation and decreased infiltration of pro-tumoral macrophages. Gene expression profiling revealed suppression of oncogenic pathways and inflammatory mediators upon ZEB1 loss, while restoration of ZEB1 rescued OS aggressive phenotype.

**Conclusions:**

Overall, our findings position ZEB1 as a critical driver of OS aggressiveness by regulating stemness, cellular differentiation, oncogenic signaling, and immune infiltration, and highlight differentiation-based therapeutic strategies as a promising avenue for osteosarcoma treatment.

**Supplementary Information:**

The online version contains supplementary material available at 10.1186/s13046-026-03757-9.

## Introduction

Osteosarcoma (OS) is a rare but highly aggressive malignant bone tumor that primarily affects children and young adults. It typically arises from abnormal growth of immature bone tissue in the long bones, especially in areas near the growth plates such as the metaphysis. The exact cell of origin is still uncertain, though it is believed that osteoblastic progenitors give rise to OS [1]. Genetic alterations, including mutations in tumor suppressors like p53 and Rb, as well as chromosomal abnormalities, alter the normal process of osteogenic differentiation [2], [3]. This disruption leads to the development of OS and classifies it as a so-called differentiation disease [4, 5].

OS often presents with lung metastases already at diagnosis, and patients with evidence of metastatic disease or having disease relapse, face a poor prognosis [6]. Current treatment strategies combine chemotherapy, using agents such as doxorubicin, cisplatin, and methotrexate, +/- ifosfamide, with surgical removal of the primary tumor. Although this approach achieves a 65% five-year survival rate for non-metastatic patients, it drops dramatically to 20–35% for those with metastasis [7, 8].

A key step in the metastatic cascade is the activation of the epithelial-to-mesenchymal transition (EMT) program, which enables tumor cells to acquire a spindle-like shape and detach from the primary tumor [9]. While EMT’s role in epithelial cancers is well established, emerging evidence shows that it also plays an important, though more plastic, role in cancers of mesenchymal origin such as OS [10, 11]. Indeed, while EMT seems paradoxical in sarcomas—which are mesenchymal tumors by definition—growing evidence indicates that many sarcomas undergo EMT-like processes and that these processes are linked to aggressive clinical behavior. Such intermediate EMT-state, often referred as “metastable” phenotype, observed in both epithelial and mesenchymal tumors, is considered a cellular state characterized by the presence of both epithelial and mesenchymal features, as well as stem cell properties [10, 11]. Among the EMT-regulating transcription factors, Zinc-finger E-box binding homeobox-1 (ZEB1) represses epithelial genes such as E-cadherin and promotes mesenchymal markers like Vimentin and N-cadherin, enhancing cancer cell motility and invasion [12]. In addition to driving EMT, ZEB1 enhances stem-like properties of cancer cells, contributing to metastasis and therapy resistance [13]. Interestingly, several pieces of evidence link ZEB1 expression to such hybrid/intermediate EMT state, at least in carcinomas, such as breast and prostate cancer [14, 15].

Although limited, a few studies have found that ZEB1 is upregulated in OS tumor samples and correlates with poor patient outcomes [16]. Furthermore, recent research shows that ZEB1 can block osteoblastic differentiation in both normal bone development and OS [17].

Based on this, we hypothesized that ZEB1 could affect OS cell behavior by sustaining stemness and mesenchymal traits while blocking differentiation. We investigated this hypothesis by inhibiting ZEB1 expression in mouse immune-competent OS cell lines and in a patient-derived xenograft (PDX) human cell line using CRISPR/Cas9 and shRNA technologies. We then evaluated the effects of ZEB1 loss on OS cellular features and tumor behavior in vitro and in vivo.

## Methods

### Cells and culture conditions

Osteosarcoma mouse cell lines, mOS69 and mOS5 were previously established in our laboratory [18] for mOS69 and unpublished results for mOS5]. Cells are grown in a humidified atmosphere at 37 °C with 5% CO_2_ in DMEM high-glucose supplemented with 10% FBS (Gibco), 1% L-glutamine, 1% Hepes, 1% Sodium Pyruvate and antibiotics (all from Lonza). Human PDX-derived cell line, PDX-OS#19-C2 ( PDX19) was kindly provided by our collaborator Prof. Katia Scotlandi (Istituto Ortopedico Rizzoli, Bologna, Italy) [19]. All cell lines are routinely tested for mycoplasma infection every 2 months.

### ZEB1 gene knock-out, silencing and over-expression

To silence ZEB1 expression we employed CRISPR/Cas9-based plasmids [20] and shRNA interfering lentiviral vectors. For the first approach we used two different guide RNAs [(gRNAs) Zeb1 1.1 (GCAGAAAGCAGGCGAACCCG) and Zeb1 2.2 (GACCAGACAGTATTACCAGG)], and a scramble one, cloned into the plasmid vector pSpCas9(BB)-2 A-GFP, containing GFP as reporter gene for selection. mOS69 cell line was transfected with Lipofectamine 2000 (Thermo Fisher Scientific) following the producer’s protocol. To select transfected cells, we FACS-sorted GFP^+^ cells and performed a single cell cloning by plating one cell/well in 96 well plate. After 2–4 weeks single cell colonies were harvested and amplified for further analysis. For shRNA approach, mOS5 and mOS69 cells were silenced for Zeb1 using two short hairpin sequences (named 850–853) cloned into the pLKO-puro lentiviral backbone. A pLKO-puro vector carrying a short hairpin sequence for eGFP was used as control. All shRNA-vectors (shRNA Mission®) were purchased from Merck. The production of lentiviral particles was performed with a third-generation packaging system by CaCl_2_-mediated transfection in 293T cells [21]. After 24 h, supernatant was removed and fresh medium added. The next day supernatants containing viral particles were collected, centrifuged and filtered. Recipient cells were seeded in 6-well plates in presence of viral supernatant (dilution 1:2 with medium). After 24 h medium was replaced with DMEM with antibiotic selection (puromycin, 4 µg/mL). The effectiveness of KO or knock-down (KD) was assessed by western blot.

For ZEB1 silencing in human PDX-derived lines two short hairpin sequences (named sh545 and sh631) in the pLKO-puro lentiviral backbone were also purchased from Merck. Lentiviral production and cell line infection were performed as above.

To overexpress ZEB1 we employed a lentiviral vector, from GeneCopoeia (pReceiver-Lv165 EX-Mm05622). As control we used the empty vector (pReceiver-Lv165 EX-NEG Lv165). Both vectors carried the EF1α promoter and puromycin as selection marker.

### Protein extraction and western blot analysis

A total of 2.5 × 10^5^ cells were seeded in 6 well plate, harvested after 48 h and lysed using RIPA buffer (Sigma Aldrich) supplemented with SDS 0,1%, Phospho STOP (Roche, PHOSS-RO), Protease inhibitor (Roche). Cell lysates were maintained in ice for 20 min, then centrifugated at 13,000 rpm for 20 min. The supernatant was harvested and protein concentration evaluated by BCA assay kit (Thermo Fisher Scientific). A total of 45 µg of proteins were mixed with NuPAGE LDS Sample Buffer 4x, NuPAGE Reducing Agent 10x and water and loaded in NuPage 4–12% Bis-Tris Gels (all from Invitrogen). Antioxidants (Invitrogen) were added in MOPS NuPAGE running buffer (Invitrogen). Total proteins were then blotted on nitrocellulose membrane (Fisher Scientific) using transfer blot buffer (Invitrogen) and membranes were incubated at 4 °C overnight with the antibody listed in Supplementary Table S1A. Protein expression was visualized by Azure western blot Imaging System (Azure Biosystem) and densitometric analysis performed by ImageJ software.

### RNA extraction and real time PCR

A total of 2.5 × 10^5^ cells were seeded in 6 well plate and harvested after 48 h. Cells were lysed directly in the plate using RNA lysis buffer and RNA extraction was performed using Quick RNA Microprep (Zymo Researcher) following the manufacturer’s procedure. Total RNA was quantified by Nanodrop (Thermo Scientific) and RNA was then reverse transcribed in cDNA by using High-Capacity cDNA KIT (Thermo Scientific). Real Time RT PCR was performed using Quant Studio 3 (Thermo Fisher Scientific) to evaluate the expression of the different genes of interest (Supplementary Table S2).

### Sarcosphere forming assay

A total of 10^4^ cells/well were seeded in ultra-low attachment 6 well plates (Corning Costar) in DMEM/F-12-HEPES and Neurobasal medium (Thermo Fisher Scientific) supplemented with B27 (Thermo Fisher Scientific), Neuro2 (Sigma Aldrich, SCM012), GlutaMax (Thermo Fisher Scientific) and 55 mM Beta-mercaptoethanol (Sigma Aldrich). After one week of culture the spheres were counted (passage 1, P1), and then disrupted as single cells to proceed to P2, following the same procedure and then, after another week, to P3.

### Osteogenic differentiation, alkaline phosphatase and alizarin red staining

Cells were seeded at the dose of 3–5 × 10^4^ in 6 well plates, in complete medium. When cells reached 65–70% of confluence, they were differentiated towards osteoblasts using MesenCult Osteogenic Differentiation Kit (StemCell Technologies) and changing the medium every 2 days. Alkaline phosphatase was measured by using ALP Staining Kit (Sigma Aldrich), following manufacturer’s protocol. Images of ALP positive cells were obtained with Nikon TE2000-S microscope. To evaluate the calcium deposits, Alizarin Red S staining was employed. Cells were maintained in osteogenic differentiation medium for at least 6 days, then fixed for 2 h with 70% ethanol, washed three times with water and then stained with 40 mM Alizarin Red S (Sigma Aldrich) pH 4.1 for 15 min. Thereafter, cells were washed with PBS 1X until complete clearance of the surface.

### Soft agar invasion assay

A total of 5 × 10^2^ cells were seeded in 12 well plates, in triplicate, in soft agar medium. For the bottom layer, a 0.5% agar solution was prepared in DMEM 10% FBS medium. For the upper layer, a 0.33% agar solution was used in which cells were added. After 14 days at 37 °C, spheroids were counted under a microscope.

### In vitro doxorubicin sensitivity assay

Doxorubicin sensitivity is measured using the CellTiter-Glo Luminescent Cell Viability Assay (Promega G 7571). Briefly, cells are seeded at the dose of 2000 cells/well in 96 W View Plates (White, clear bottom, 96-well, tissue-culture treated, with lid cat. 6005181 Reavvity), in a volume of 100 µl of complete DMEM medium and incubated at 37 °C overnight. Doxorubicin (adriblastin 50 mg/25 ml, Pfizer) is added at the following concentrations: 0.5, 1, 2, 4 u/ml. Cell viability is measured at 24 and 48 h after treatment. Carefully aspirate all medium from the plate, ensuring wells are completely dry. CellTiter-Glo Reagent, previously diluted in PBS 1:4, is added to each well. The plate are incubated for 10 min at RT with shaking and then luminescence is read with Tecan plate reader.

### ROS induction and detection

ROS were detected using the CellROX™ Green Flow Cytometry Assay Kit following the manufacturer’s instructions. Briefly, cells were seeded at the dose of 3 × 10^4^ in 6 well plates, in complete medium and let adhere o/n. The day after cells were treated with the oxidant reagent TBHP (200uM) for 4 hr and then Cell Rox green reagent was added for 60’. Cells were recovered by trypsinization and analyzed by flow cytometry within 1 h.

### In vivo experiments

Eight-week-old female BALB/c mice were purchased from Charles River Laboratories (Calco, Italy). For subcutaneous tumor growth, 2 × 10^5^ cells for mOS69 and 1.5 × 10^5^ for mOS5 were injected in the left flank of BALB/c mice. Tumor size was measured with a caliper and volume calculated in mm^3^ as [D x d^2^/2].

For the evaluation of metastatic potential, 10^5^ cells were injected intravenously in BALB/c mice. Lungs were isolated from mice after 4 weeks and analyzed by flow cytometry for immune cell infiltrate and formalin-fixed and paraffin-embedded for histological analysis and metastasis count.

### Immunohistochemistry and histological staining

Tumors were excised, washed in PBS, fixed in 10% neutral buffered formalin, and paraffin embedded. Four-micrometers-thick mouse tissue sections were deparaffinized, rehydrated and H&E stained for tumor histotype definition. Masson’s Trichrome staining were performed on murine samples with Diapath kit following the manufacturer’s instructions. Masson’s trichrome images were analyzed using the “Area Quantification v2.4.2” algorithm of HALO software (Indica Labs) to quantify the blue-stained collagen fibers. Five non-overlapping fields per sample were evaluated at medium-power magnification (x200).

### Flow cytometry analysis

For the analysis of infiltrating leukocytes, tumors and lungs were mechanically dissociated in single cells, washed and filtered with 70 μm cell strainer (Fisher Scientific). ACK 1x solution was used lo lyse erythrocytes. Cells were then stained for the evaluation of myeloid and lymphoid subsets (supplemental Table S1B for the list of antibodies used). For T regulatory cell detection, cells were fixed and permeabilized with FOXP3 transcription Factor Staining Buffer Set (eBioscience). Samples were acquired with a BD Fortessa Cytometer (Becton Dickinson) and data analyzed with FlowJo 10 software.

### Gene expression analysis on mouse samples

Total RNA was extracted from ZEB1KO clones (B8 and E4), control clone (G5P1) and parental cell line, as above described by using Quick RNA Microprep (Zymo Researcher) following the manufacturer’s procedure. Nucleic acid was quantified by Qubit 2.0 Fluorimetric Assay (Thermo Fisher Scientific) and the quality was checked by TapeStation4200 (Agilent Technologies). Three biological replicates (different in vitro passages and different moments of collection) were used for each cell line. Microarray analysis was performed with Clariom S Assay, mouse (ThermoFisher) containing > 20,000 well-annotated genes. Briefly, the Affymetrix GeneChip WT Pico Kit was used for cDNA preparation, biotin labelling, and cRNA synthesis starting from 200 ng of total RNA. The arrays were subsequently incubated for 16 h in an Affymetrix GeneChip 645 hybridization oven and processed with the GeneChip Hybridization. Washing and staining was performed using the GeneChip HT hybridization, Wash and Stain Kit and with the Affymetrix Genechip fluidics station 450. The chips were scanned with an Affymetrix GeneChip Scanner 3000 with default manufacturers’ settings and raw data were acquired with the AGCC scan control v4.0.

Raw data were imported into R (version 4.5.1) and processed through standard Bioconductor packages. Using oligo (v. 1.72.0), CEL files were read and pre-processed with the implemented Robust Multi-Array Average (RMA) algorithm, performing background correction, quantile normalization and probe summarization. Array layout and probe-level mapping were provided by the platform design package pd.clariom.s.mouse (v. 3.14.1). Probe sets identifiers in the resulting log2-scale gene expression matrix were annotated to gene-level information with clariomsmousetranscriptcluster.db (v. 8.8.0), retrieving corresponding Entrez gene identifiers and official gene symbols. Probe sets lacking an Entrez identifier were removed and duplicates were collapsed by maximal row variance with WGCNA (v. 1.73). Standard quality-control diagnostics were generated with arrayQualityMetrics (v. 3.64.0), including array intensity distributions, MA plots, principal component analysis and hierarchical clustering. Differential expression analysis was conducted using limma (v. 3.64.3) while GSEA with clusterProfiler (v. 4.16.0).

### Statistical analysis

Data from real-time PCR, sarcosphere forming assays, soft agar and the analysis of metastasis are represented as mean +/- SD with one-way ANOVA adding the Bonferroni’s multiple comparison test, with a single pooled variance. Tumor volumes are analyzed with two-way ANOVA. The analysis of the immune infiltrate was performed with one-way ANOVA. Statistical significance was considered at *p* < 0.05.

## Results

### ZEB1 inhibition reduces OS stemness features and aggressive traits in vitro

To evaluate the effects of ZEB1 inhibition on OS aggressive features, its genetic knock-out (KO) was obtained using CRISPR/Cas9-based plasmids in mOS69 OS cell line. Western blot analysis allowed the identification of ZEB1KO clones (Supplementary Figure S1A). Three ZEB1KO clones (B8, E4 and H3) and a scramble control (SCR-G5P1) were selected for further experiments, sequenced to verify the presence of indels in genomic DNA and re-checked by WB for ZEB1 absence (Supplementary Figure S1B).

**Fig. 1:**
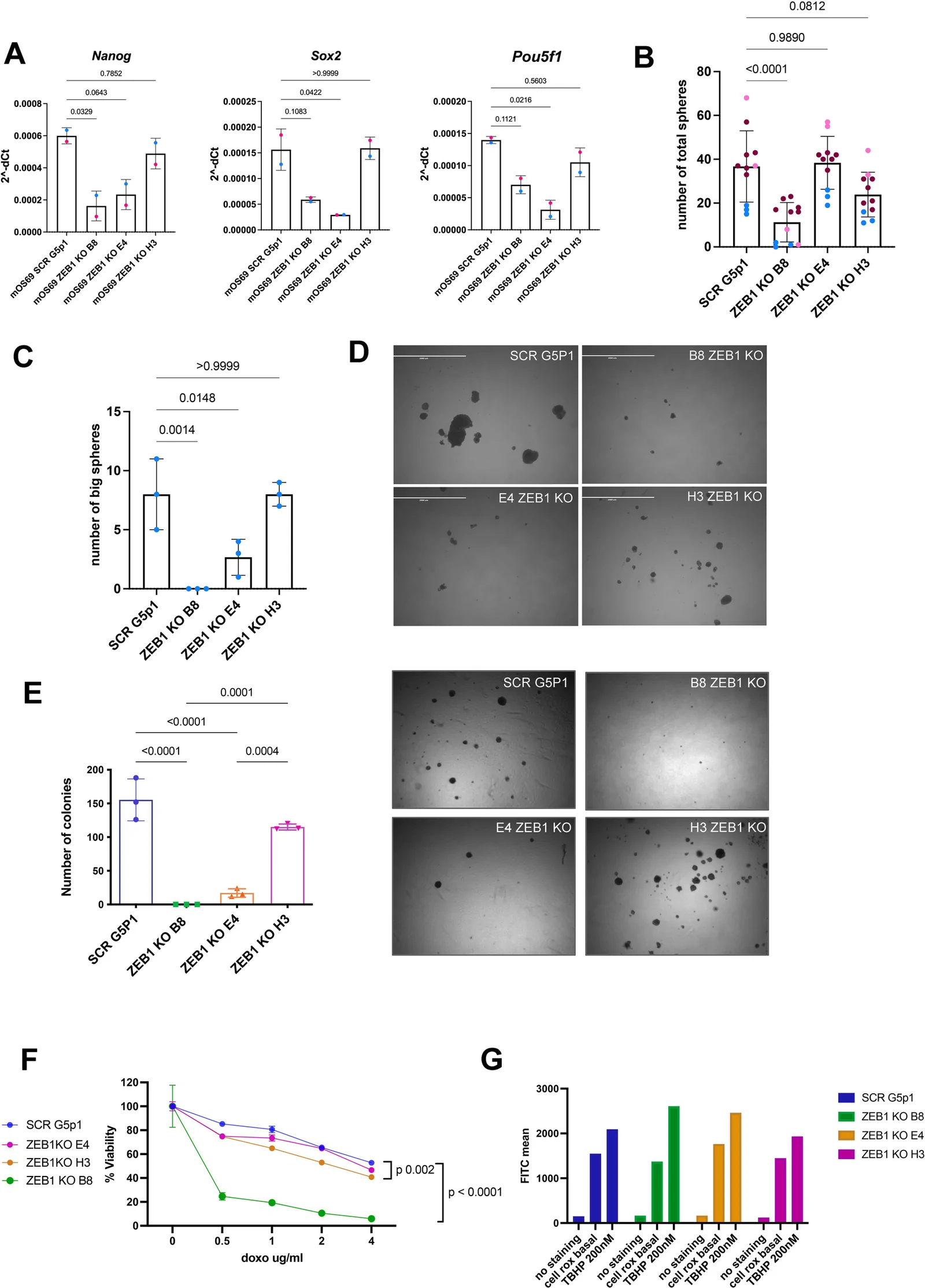
ZEB1 knock-out reduces aggressive traits in mOS69 cells in vitro. **A** Real Time PCR to evaluate the expression of Nanog, Sox-2 and Pou5f1 inZEB1KO clones and control one. Data are represented as 2^− dCT^. Two biological samples (in two technical replicates) for each cell lines are used and the experiment has been performed twice. Each dot is the mean of the samples in each experiment. **B** Sarcophere forming assay with mOS69 ZEB1KO clones and SCR G5P1 control one. Total number of spheres are shown. The graph is a pool of three experiments and each experiment has been done with three-six replicates (each experiment has a different color code). **C** Graph showing only the number of big spheres in one representative experiment from B.; **D** Representative images of the sarcopheres formed. Sarcopheres has been counted in blind by two operators. Side bar 2 mm. **E** Soft agar assay on mOS69 ZEB1 KO clones and controls. **D** Representative images of the colonies formed. **F** In vitro sensitivity to doxorubicin after 24 h of treatments. Viability measured with Cell Titer Glow. **G** ROS induction upon treatment with TBHP 200 nM for 4 h. ROS has been measured by flow cytometry with CellROX™ Green Flow Cytometry Assay Kit. Experiment has been performed twice with technical replicates. Statistical analysis: ordinary one-way ANOVA, but for panel F for which nonlinear regression has been used

To address the effect of ZEB1 deletion on OS phenotype, we initially focused on its role in the maintenance of stemness features analyzing the expression of stemness-related genes (*Nanog*, *Sox2* and *Pou5f1*), which were found significantly down-regulated when ZEB1 was deleted, with few differences among the clones (Fig. 1A).

Notably, the reduction in gene expression was associated with a functional impairment of the stemness potential, evaluated by sarcosphere forming assay (Fig. 1B). ZEB1KO clones showed smaller and fewer spheroids, although with some differences among the three KO clones. Clone B8 was almost unable to grow as spheres, while clone E4 produced a similar number of spheroids to the control, but of much smaller size (Fig. 1C-D).

To further investigate whether ZEB1 contributed to OS aggressiveness, we evaluated the effect of ZEB1 deletion in a soft-agar colony formation assay, an in vitro experimental setting assessing anchorage-independent growth and potential invasion ability. ZEB1KO clones produced lower number of colonies than control or were completely unable to form colonies. Also in this setting, clone B8 appeared to be the most affected by ZEB1 absence (Fig. 1E).

To exclude that the results above were due to a different proliferation rate, we tested the in vitro proliferation of the different ZEB1KO clones in comparison with SCR control and found no significant difference among ZEB1-competent and deficient clones (Supplementary Figure S1C).

Since the three ZEB1KO clones, despite being completely knock-out for its expression, behaved differently, to confirm our findings and exclude that they could be clone- or cell line-specific, we down-modulated ZEB1 expression in an additional mouse OS cell line, mOS5, using a shRNA interference with lentiviral vectors and antibiotic selection of the bulk population, an approach that is likely more representative of the whole parental cell line population than the single cell clones. Once assessed ZEB1 down-modulation by western blot (Supplementary Figure S2A-B), we evaluated in this additional model the effect on *Nanog* and *Pou5f1* stemness-related genes that showed a reduced expression, although not statistically significant (Suppl Figure S2C). *Sox2* expression was down-modulated in only one shZEB1 line, likely suggesting a different regulation of these genes in the mOS5 model. Functionally, both shZEB1 lines showed a reduced number of colonies in soft-agar (Suppl Figure S2D). Interestingly, the colonies formed by mOS5 control (SCR shGFP) cell line produced some sprouting-like structures, which could be considered a feature of invasion ability (Suppl. Figure S2E) and that were absent in ZEB1-silenced cells.

### ZEB1-deficient OS cells are more sensitive to chemotherapy in vitro

To further assess how ZEB1 inhibition influences OS cell behavior, we evaluated their in vitro sensitivity to doxorubicin—an essential component of the standard multidrug chemotherapy regimen used in OS clinical management. As shown in Fig. 1F, ZEB1KO B8 clone and to a lesser extent E4 clone, were more sensitive to doxorubicin treatment than control SCR G5p1 clone (IC50 SCR G5p1:4.048 µg/ml; ZEB1KO E4: 3.09 µg/ml; ZEB1KO H3:2.133 µg/ml; ZEB1KO B8: 0.192 µg/ml).

As doxorubicin induces in OS cells mitochondrial ROS generation, which then triggers the apoptotic pathways [22], we checked whether ZEB1KO cells were more prone to ROS production. Consistently with this idea, ZEB1KO B8 and H3 clones produced more ROS than the control counterpart when treated with the oxidant agent TBHP (Fig. 1G).

### ZEB1 inhibition restores osteoblastic differentiation ability of OS cells

**Fig. 2:**
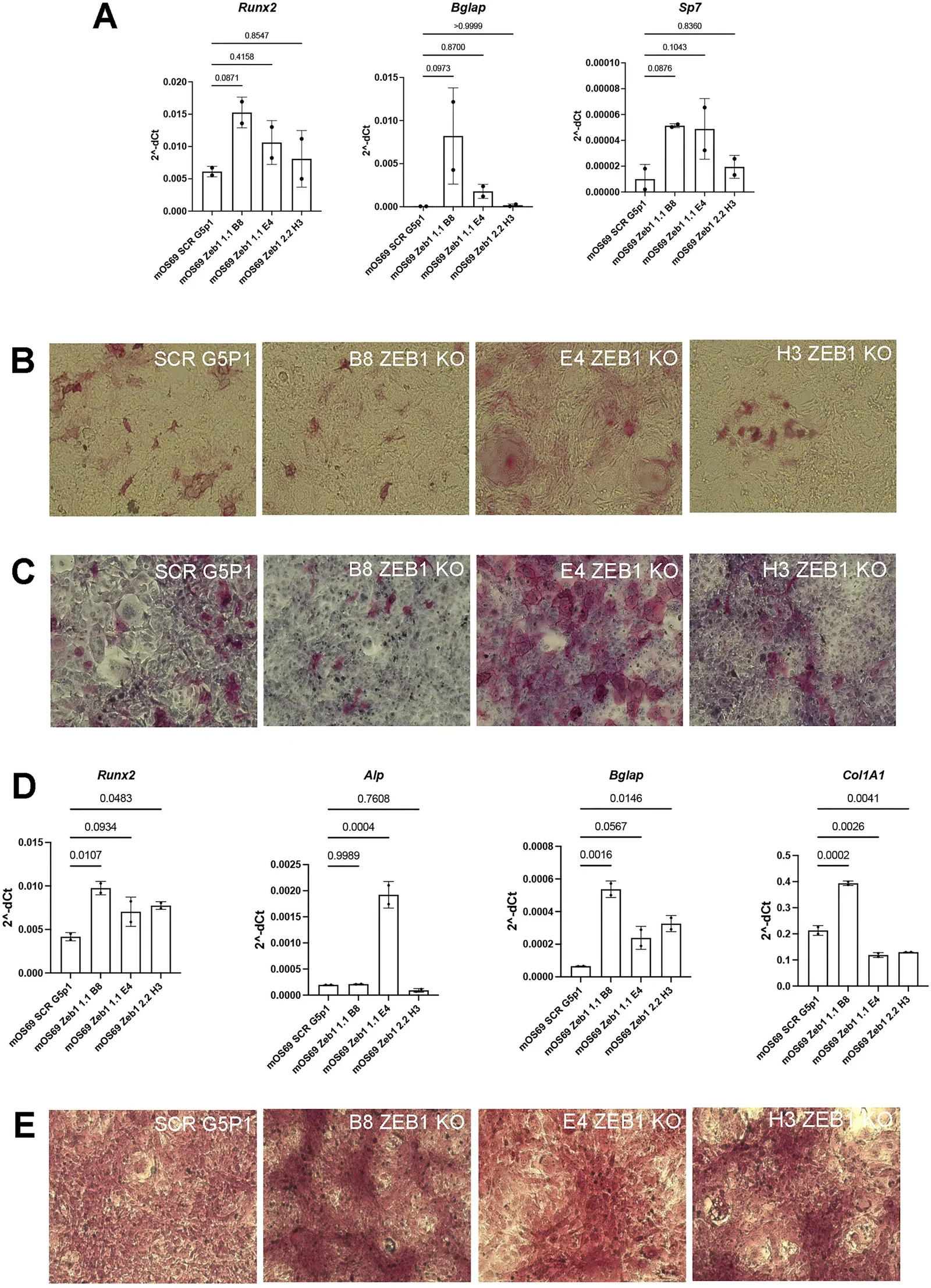
ZEB1 deletion stimulates the expression of osteoblastic-related genes and restores osteogenic differentiation. **A** Real Time PCR analysis on mOS69 ZEB1 KO clones and SCR control of the expression of *Runx2*,* Sp7* and *Bglap*. Data are represented as 2^− dCT^. Two biological samples (in two technical replicates) for each cell lines are used and the experiment has been performed twice. Each dot is the mean of the samples in each experiment; **B-C** Enzymatic staining for ALP after three days of in vitro culture in DMEM complete medium (**B**) and after 6 days in osteogenic differentiation medium (**C**). ALP positive cells are characterized by a red/purple colouring. **D** Real Time PCR for the expression of early-mid (*Alp* and *Sp7*) and late (*Col1A1* and *Bglap*) osteoblastic-related genes, along with the master osteogenic transcription factor *Runx2*. Data are represented as 2^− dCT^. **E** Representative images of Alizarin Red-S staining. Calcium deposits are shown as dense red-brown areas. (10X magnification for all images). Statistical analysis for RT-PCR analysis: ordinary one-way ANOVA

Since the block of osteogenic differentiation of progenitor cells towards osteocytes is crucial to allow OS development, we investigated whether ZEB1 inhibition had any impact on OS differentiation in our mOS69 model. We tested the expression of *Runx2*, the master regulator of osteogenic commitment and differentiation, and of *Sp7* (osterix) and *Bglap* (osteocalcin), other two genes involved in such process. The expression of all three genes was increased in ZEB1-deficient cells (Fig. 2A), with the highest levels in clone B8, the one most impaired also in sarcophere formation and invasiveness. The different expression levels among ZEB1 clones suggested that they could be characterized by diverse differentiation stage. This hypothesis was supported by alkaline phosphatase (ALP) staining, both at steady state and upon culture in osteogenic differentiation medium. Unexpectedly, clone E4 was the most ALP-positive, in both conditions, while clone B8 showed a much lighter staining (Fig. 2B-C). As ALP expression is associated with early/mid stage of differentiation, we hypothesized that clone B8 could reside in a more advanced stage of differentiation than the other ZEB1KO clones. Therefore, we additionally evaluated the expression of differentiation-related genes after 6 days of osteogenic differentiation, including *Alp* and *Col1a1*, as marker of early and mid-late state, respectively. RT-PCR data confirmed that clone E4 highly expressed the sole early osteogenic marker *Alp*, while clone B8 those of the mid-late differentiation markers, *Bglap* and *Col1a1*, along with the highest expression of *Runx2* (Fig. 2D).

To further strength this hypothesis, we performed Alizarin Red-S staining to highlight calcium deposition, which is an event occurring during late stages of osteoblastogenesis. Accordingly, clone B8 showed the strongest Alizarin Red-S staining, supporting the idea of a more advanced stage of differentiation than the other ZEB1KO clones (Fig. 2E).

### ZEB1 inhibition limits OS aggressiveness in vivo

**Fig. 3:**
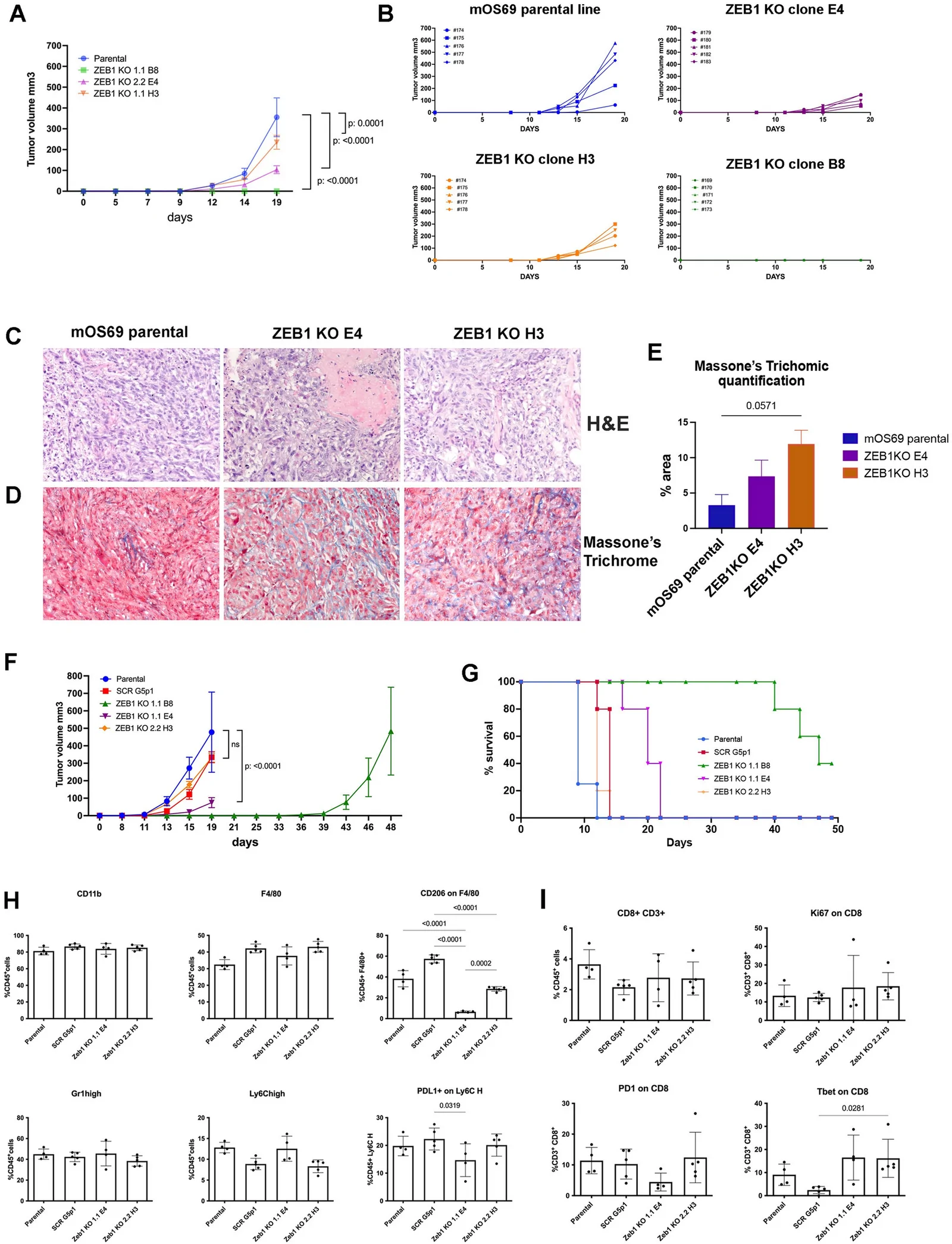
ZEB1 inhibition reduces primary tumor growth in vivo of mOS69 ZEB1 KO clones. **A-B** Tumor growth of mOS69 ZEB1 KO clones and control line injected subcutaneously in immunocompetent BALB/c mice. The graph in panel A represents the mean tumor volume of all mice in each experimental group, while the graphs in panel **B **show the tumor volume of each individual mouse across the different experimental groups. The experiment has been performed three times with 5 animals for each one. Statistical analysis used: two-way ANOVA. **C** Hematoxylin and eosin staining of tumor masses. **D** Masson’s Trichrome staining to visualize collagen matrix deposition (shown in blue). **E** Quantification of Masson’s Trichrome staining (see M&M for details). **F** Tumor growth of mOS69 ZEB1 KO clones and control line injected subcutaneously in immunocompetent BALB/c mice with extended observational time for ZEB1KO clone B8. A different experiment from panel A-B is shown. 5 mice per group. Statistical analysis used: two-way ANOVA. **G**. Survival analysis of tumor growth experiment shown in panel F. **H-I**. Flow cytometry analysis of immune cells infiltrating subcutaneous tumors; panel **H**: myeloid cells (percentage of total CD11b, F480 macrophages, CD206 + M2-like macrophages, Gr1high granulocytic cells, Ly6C high monocytic cells and PD-L1 + cells on monocytes; panel **I**. CD8 + T lymphocytes, percentage of CD8 + CD3+ cells, expression of ki67 (proliferation marker), PD1 and Tbet on CD8 + CD3+ gated cells. Five animals per group are analyzed. Experiment performed three times. Statistical analysis: ordinary one-way ANOVA

To investigate the relevance of ZEB1 for OS aggressiveness in vivo, we subcutaneously injected mOS69 ZEB1 KO clones and parental control line in syngeneic BALB/c mice. As shown in Fig. 3A-B, the absence of ZEB1 in neoplastic cells restrained tumor growth, at different levels in the three KO clones. At the endpoint, tumor volume of ZEB1KO clone H3 was slightly smaller than control tumors, clone E4 was significantly inhibited and clone B8 showed no take (Fig. 3A-B).

Histological analysis highlighted a poorly differentiated phenotype in the control tumors with minimal matrix deposition and cells with spindle-like shape morphology, features characteristic of an aggressive phenotype (Fig. 3C). On the other hand, ZEB1KO clones E4 and H3 showed a decrease in cell cohesion and increase in the frequency of apoptotic/necrotic figures and in matrix deposition. Masson’s Trichome staining confirmed scarce collagen matrix, mainly focused in the center of the lesion in the control tumors, while lesions form ZEB1KO clones show a more consistent collagen mesh with longitudinal fibers between tumor cells, and presence of lacunae, phenotypic aspects that may be related with an increased differentiation rate (Fig. 3D-E). Of note, since clone B8 showed no take at all when all other groups were sacrificed for humane endpoints, we are unable to provide the E/E and Trichrome’s staining images for this clone.

To verify whether ZEB1KO B8 clone completely lost its tumorigenic potential or was only severely impaired, we performed a survival experiment (based on humane endpoints), euthanizing the animals at different time points, when the tumor lesions reached a maximum of 10 mm in diameter for humane reasons. As shown in Fig. 3F-G (and Supplementary Figure S3), while mice bearing control and clone E4 and H3 tumors were sacrificed at day 19, animals injected with clone B8 cells, reached day 50, with some mice with tumor lesions of very small volume (below 50 mm3), doubling the survival of the animals.

We confirmed the in vivo result also in the mOS5 model, in which ZEB1 expression has been down-modulated by shRNA interfering. As shown in Supplemental Figure S2F, both shZeb1 lines showed reduced tumor growth when compared with control shGFP line. Notably, also in this setting the in vivo results were in line with the in vitro data on the soft-agar colonies assay (Supplemental Figure S2D-E), with shZeb1 853 being less aggressive than shZeb1 850.

In a previous study we have demonstrated that an increased tumor cell differentiation and matrix deposition were associated with a reprogramming of the tumor immune infiltrate [18]. Since ZEB1KO tumors were, indeed, characterized by such features, we explored whether the absence of ZEB1 in tumor cells had any impact on immune cell infiltration. Multiparametric flow cytometry analysis of the myeloid compartment in tumor samples from mOS69 ZEB1KO clones showed a significant reduction in the number of pro-tumoral F4/80 + macrophages, expressing the M2-like marker CD206 in particular in clone E4, the one with severely reduced tumor growth (Fig. 3I and Supplementary Figure S4A-C for gating strategies). In addition, monocytic cells highly expressing Ly6C (Ly6C H) were characterized by a lower expression of PD-L1, potentially suggesting a less suppressive environment in clone E4 and consistent with the reduced expression of CD206 in macrophages. Of note, ZEB1KO clone H3 did not show significant difference in myeloid cell infiltration from the control lines, in agreement with both in vitro behavior and in *vivo* tumor growth.

**Fig. 4:**
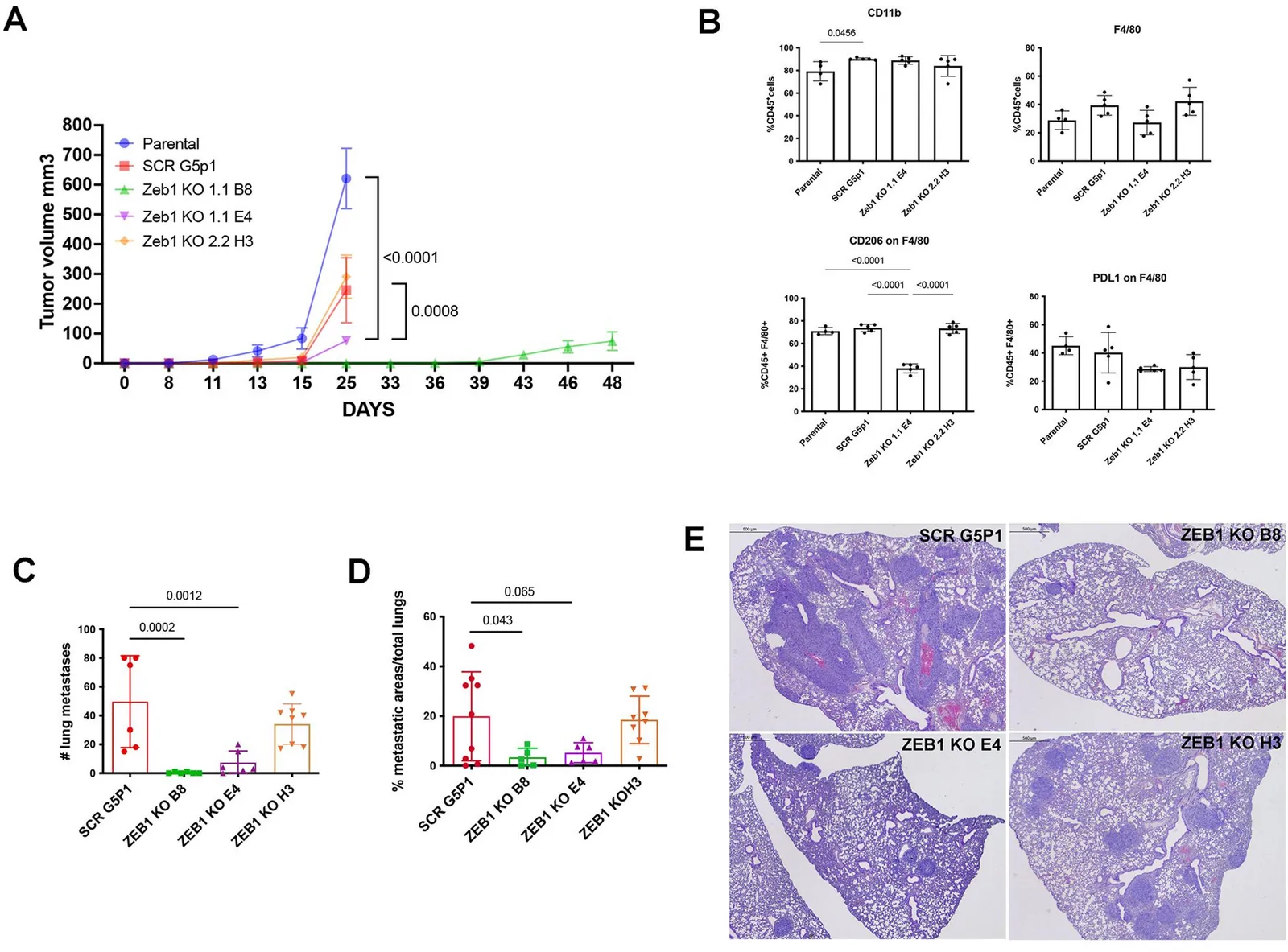
ZEB1 KO OS cells grow similarly in immune-competent and -deficient mice and are less efficient in lung metastatic dissemination. **A** Tumor growth of mOS69 ZEB1 KO clones and control lines injected subcutaneously in immune-deficient nude mice. Graph represents the mean of all mice tumor volume of each experimental group. The experiment has been performed once with 5 animals for each group. Statistical analysis used: two-way ANOVA; statistical significance values for endpoint are shown. **B** Flow cytometry analysis of myeloid cells infiltrating subcutaneous tumors from the experiment shown in panel A. Total percentage of CD11b in CD45 + cells, F480 + macrophages, CD206 + M2-like macrophages, and PD-L1 + expression on macrophages. Statistical analysis: ordinary one-way ANOVA. **C** Number of lung metastases upon intravenous injection of ZEB1KO clones and scramble control. Count has been done in blind by two operators. **D** Percentage of metastatic areas on total lung area. **E** Representative images showing lungs from mice injected with ZEB1 KO clones and SCR control clone. Side bar 500 μm. Eight mice per group has been used. Statistical analysis: ordinary one-way ANOVA

The analysis of T cell compartment, did not highlight major differences neither for CD8 T cells, beside a trend toward a more activated phenotype (increased ki67 and Tbet expression and decreased PD-1, at least for clone E4; Fig. 3L), nor CD4 T cell infiltration (Supplementary Figure S4D), suggesting that adaptive immune cells are not key players in ZEB1-mediated role in OS aggressiveness. To further verify this issue, we injected ZEB1KO clones and control lines into immune-deficient nude (nu/nu) mice. As shown in Fig. 4A the differences in growth between ZEB1-competent and KO cell lines were maintained in immune-deficient mice, suggesting that T cells were not actively involved in the reduced aggressiveness mediated by ZEB1 deficiency. In line, the analysis of tumor infiltrating myeloid cells gave comparable results with those obtained in immune competent mice, with reduced number of CD206 + macrophages in clone E4 (Fig. 4B and Supplementary Figure S4A for gating strategy). Of note, all lines showed a trend of reduced growth in nude mice when compared to the same lines in BALB/c, immune competent, animals.

As the main site for OS metastatic dissemination is the lung, we investigated whether ZEB1 absence affected the ability of tumor cells to metastasize to this organ. To this aim, we intravenously injected ZEB1KO clones and the control one and performed histological analysis of the lungs to evaluate the number and size of the metastatic lesions. Consistently with the results on primary tumour growth, clone B8 was the most impaired in lung dissemination (Fig. 4C-E).

To assess whether our findings in the murine setting could have a translational relevance, we tested the impact of ZEB1 down-modulation in a PDX-derived cell line, PDX-OS#19-C2 [19] (hereafter PDX-19) in vitro. As shown in Supplementary Figure S4, ZEB1 silencing was able to reduce the stemness potential in PDX19 cells, as assessed by sarcophere forming assay, and to foster osteogenic differentiation (ALP staining), with sequence sh631 showing a higher ALP positivity than sequence sh545, in line with the higher silencing efficacy of the former (Supplementary Figure S5).

### ZEB1 inhibition down-modulates pathways related to cancer aggressiveness

**Fig. 5:**
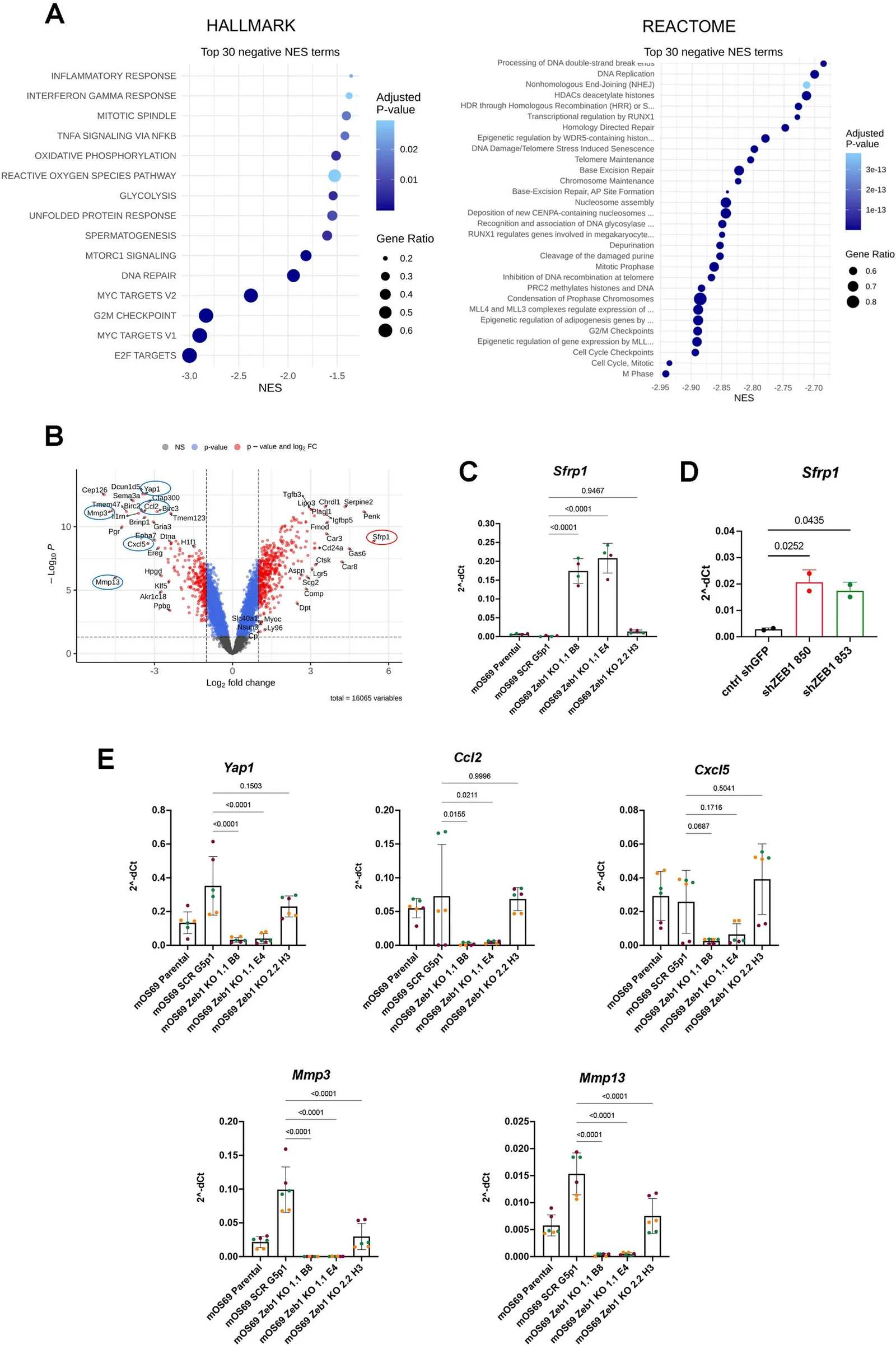
ZEB1 deletion down-regulates pathway related to cancer survival and aggressive behavior. Gene expression profiling of in vitro-cultured cell lines for B8 and E4 ZEB1KO clones in comparison with parental mOS69 line and scramble control G5P1 clone has been performed with Clariom S microarrays. **A** Pathway enrichment analysis using Hallmark and Reactome databases showing the top 30 down-modulated pathways between ZEB1KO clones and controls. **B** Volcano plot of differentially expressed genes of ZEB1KO clones versus parental mOS69 line and scramble control G5P1 clone (see Supplementary Table S3 for a complete list of DEGS (logFC: >1 and <-1, FDR: < 0.005). **C** RT-PCR analysis of the expression of *Sfrp1*, the most up-regulated gene in ZEB1KO clones. Analysis performed on two biological samples different from those of the gene expression analysis and experiment was performed twice (4 samples total). **D** RT-PCR analysis of the expression of *Sfrp1* on mOS5 line silenced for Zeb1 and in control shGFP line. Two biological replicates. Statistical analysis used: one way ANOVA. **E** RT-PCR analysis of the expression of *Yap1, Ccl2, Cxcl5, Mmp3* and *Mmp13* to confirm the gene expression data. Analysis performed on different biological samples from the gene expression analysis. Statistical analysis used: one way ANOVA

To have a more comprehensive view of the impact of ZEB1 inhibition on OS cells, we performed a gene expression profiling (GEP) of B8 and E4 ZEB1KO clones and controls (parental cell line and scramble control clone G5P1). Gene set enrichment analysis (GSEA) showed a down-modulation of several pathways linked to proliferation and survival in ZEB1KO clones, such as mTORC1, MYC and E2F along with glycolysis and oxidative phosphorylation, all pathways that sustain cancer aggressiveness and dissemination showing consistence between GEP and functional findings (Fig. 5A).

Among differentially expressed genes (DEGs), *Sfrp1*, an inhibitor of the wnt/b-catenin signaling pathway, was the most upregulated in ZEB1KO clones [logFC: 5.39864167; FDR: 1.77261731141219E-12 (Fig. 5B)], RT-PCR analysis confirmed Sfrp1 up-regulation in independent biological replicates of ZEB1KO clones (Fig. 5C). Of note, in this analysis we also included samples from clone H3 that according to previous functional data shows a behavior similar to parental line and control clone. The analysis showed negligible *Sfrp1* levels, comparable to controls. We confirmed a reduction of *Sfrp1* expression also in mOS5 cells silenced for ZEB1 (Fig. 5D).

In agreement with the decreased macrophagic infiltration in in vivo ZEB1KO tumors, *Ccl2* and *Cxcl5* expressions were significantly reduced in in vitro ZEB1-deficient cells (*Ccl2*: logFC: -3,34630483; FDR: 2,0081E-15; *Cxcl5*: logFC: -3,06814335; FDR: 4,6863E-12) in comparison to control lines (Fig. 5B). Furthermore, also *Mmp3* and *Mmp13*, known to be involved in invasion and dissemination, were down-regulated in ZEB1KO clones (*Mmp3* logFC − 4,47333191; FDR: 2,569E-14; *mmp13*: logFC: -4,25168465; FDR: 6,1269E-10), as well as *Yap1* expression (logFC: -3,30270728; FDR: 4,4015E-18), another relevant molecule for tumor progression. The down-modulation of these genes observed in the GEP data were confirmed, on different biological replicates, by RT-PCR (Fig. 5E).

#### ZEB1 complementation reactivates aggressive traits in OS ZEB1KO cells

**Fig. 6:**
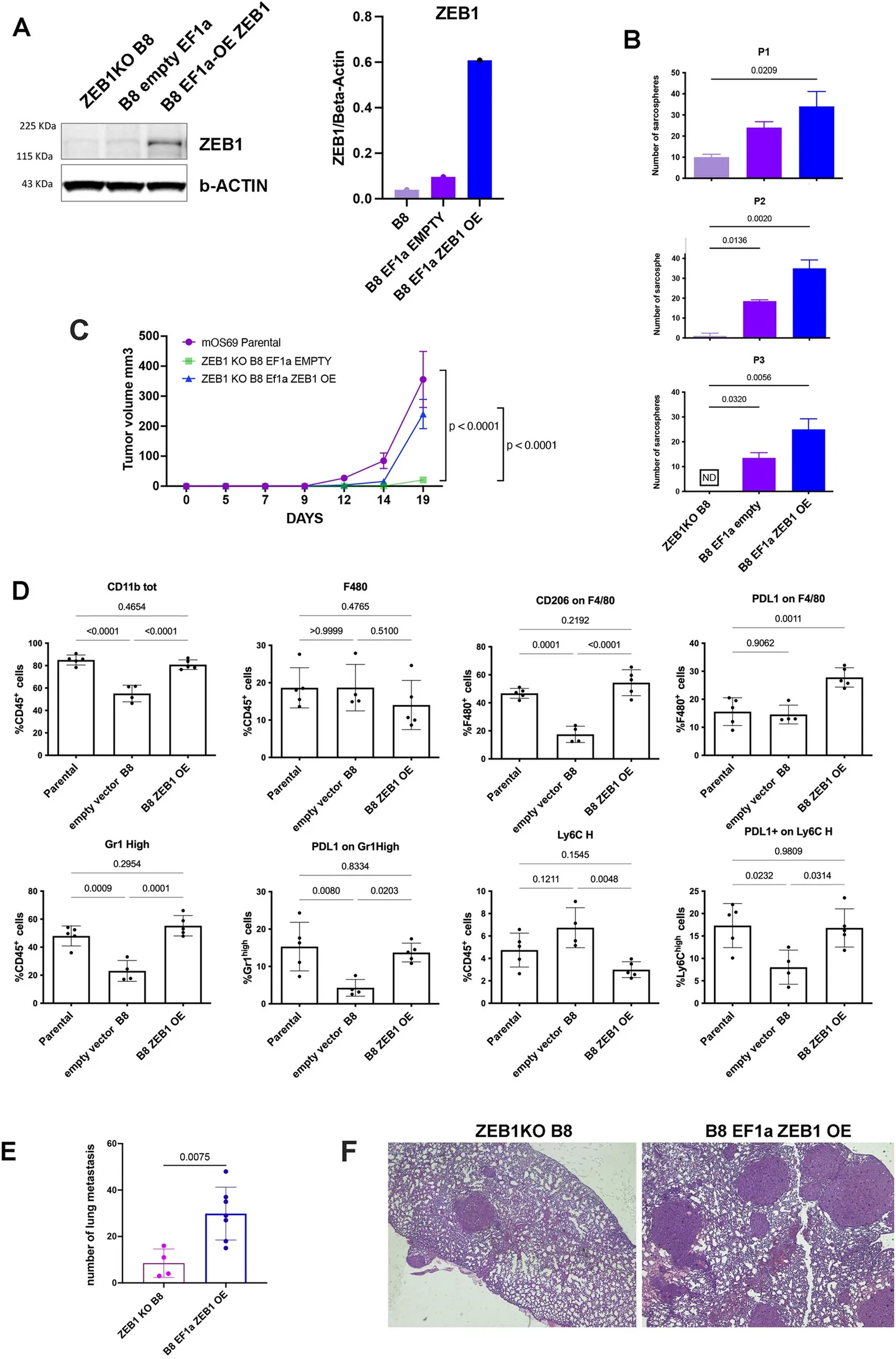
Restoration of ZEB1 expression in ZEB1KO B8 clone reactivates its aggressive traits. **A** Western blot analysis for ZEB1 expression after transduction in ZEB1KO B8. An empty vector has been used for control. Right panel: densitometric analysis of western blot image. **B** In vitro sarcosphere forming assay over three passages, in ZEB1KO B8 clone, B8 transduced with empty vector for control and with ZEB1 over-expressing vector. Experiment performed once with three replicates. Statistical analysis used: one-way ANOVA. **C** Subcutaneous tumor growth of ZEB1KO B8 clone overexpressing ZEB1, in comparision with parental mOS69 line and B8 clone transduced with empty vector. Five mice per group have been injected. Experiment performed twice. **D** Flow cytometry analysis of myeloid cells infiltrating subcutaneous tumors from experiment shown in panel C. Total percentage of CD11b cells, F480 macrophages, CD206 + M2-like macrophages, PDL1 + macrophages, Gr1high granulocytic cells, Ly6C high monocytic cells and PD-L1 + cells on granulocytic and monocytic cells. Statistical analysis used: one way ANOVA. **E** Number of lung metastases of ZEB1 over-expressing clone B8 in comparison with ZEB1KO clone B8. **F** Representative images of lung from mice injected with ZEB1 over-expressing clone B8 cells and ZEB1KO B8 as comparison. Four to seven mice per group. Experiment performed once. Statistical analysis used: one way ANOVA

To mechanistically prove that ZEB1 was responsible for the aggressive phenotype, we restored its expression in ZEB1KO clone B8, the least aggressive clone because of ZEB1 deletion. Once verified the level of ZEB1 re-expression by western blot (Fig. 6A), we tested the recovery of stemness potential that was confirmed by the restored ability to form sarcospheres (Fig. 6B).

In vivo, re-expression of ZEB1 in KO clone B8 completely restored its ability to form tumor masses, although not at the same extent as parental mOS69 line (Fig. 6C).

Interestingly, flow cytometry analysis of tumor immune infiltration clearly showed a restoration of the immune suppressive microenvironment with percentage of CD206 + M2-like macrophages recovered to the level of parental mOS69 line. Likewise, the expression of PDL1 on both monocytic and granulocytic cells is comparable to that of parental control line ((Fig. 6D). Of note, being the volume of tumors from ZEB1KO B8 clone transduced with the empty vector much smaller than those from parental and B8 clone over-expressing ZEB1, immune infiltration was generally lower.

Similarly, the metastatic potential, as assessed by direct intravenous injection of tumor cells, was recovered when ZEB1 expression was restored (Fig. 6D-E).

## Discussion

**Fig. 7:**
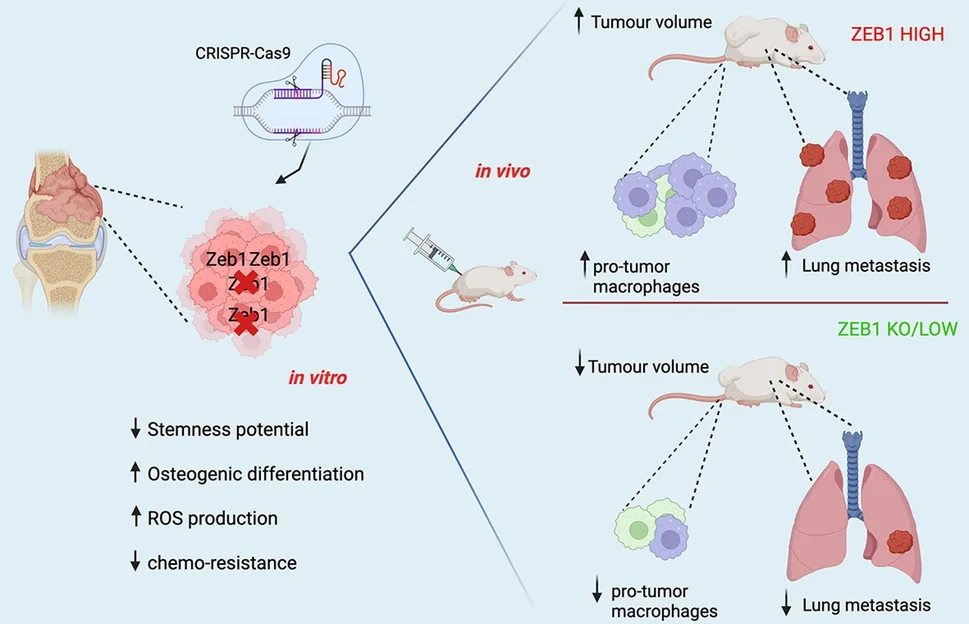
Schematic representation of the effects of ZEB1 deletion on in vitro and in vivo behavior of OS cells. Art created by BioRender

This study establishes ZEB1 as a relevant molecule for OS aggressiveness, by combining genetic knockout, shRNA silencing, and re-expression approaches in murine and patient-derived OS cellular models. We show here that ZEB1 inhibition reduces stemness features and invasiveness, restores osteoblastic differentiation, enhances chemosensitivity to doxorubicin, and limits tumor growth and metastatic dissemination (Fig. 7).

We demonstrate that ZEB1 deletion down-regulates stemness-associated genes (*Nanog*,* Sox2* and *Pou5f1*) and impairs sarcosphere formation, consistent with prior reports in epithelial tumors (prostate, colon and pancreatic) sustaining ZEB1 role in the maintenance of stemness programs [23, 24]. The functional loss of anchorage-independent growth and the severely reduced ability to form colonies in soft-agar in ZEB1-deficient cells further underscores its role in tumor aggressiveness. Moreover, ZEB1-KO cells were more sensitive to doxorubicin and to ROS inducing agent (TBHP), in line with ZEB1 regulation of DNA damage response and therapy resistance [13].

The inability of osteogenic differentiation is a hallmark of OS pathogenesis [25]. Wagner et al. showed that variable expression of osteogenic markers correlates with tumor aggressiveness and therapy response [26]. In this context, Ruh et al. (2021) provided evidence that ZEB1 directly restrains osteoblastogenesis during bone development and OS progression. In line with these findings, our work shows that ZEB1 inhibition restores expression of differentiation-related genes, such as *Runx2*, *Sp7*, and *Bglap*, promotes ALP activity, and enhances calcium deposition, indicating increased osteogenic differentiation. Notably, ZEB1KO clone B8 displayed advanced differentiation and severely reduced tumorigenicity, suggesting that ZEB1 suppression can drive OS cells toward terminal differentiation.

The correlation between reduced stemness potential and increased osteogenic differentiation suggests that as cancer cells lose their stem-like properties, they may gain the ability to differentiate into less aggressive, more specialized cell types. Our findings align with recent work by Tu et al., which demonstrated that inhibiting stemness pathways enhances osteogenic differentiation [27]. From a translational perspective, these insights on stemness regulation and osteogenic differentiation underscore the therapeutic potential of differentiation-based strategies in osteosarcoma treatment. Along this line, a recent work from Arima et al. demonstrated that treating OS cells with differentiation-inducing agents reduced stemness marker expression and enhanced osteogenic differentiation [28].

In our in vivo settings, the absence of ZEB1 markedly impaired tumor growth and metastatic lung colonization, with associated histological signs of increased differentiation and matrix deposition. These phenotypes coincided with altered immune microenvironment composition, particularly decreased pro-tumoral M2-like macrophages and reduced PD-L1 expression on myeloid cells, suggesting that ZEB1 influences the tumor immune niche potentially to favor immune suppression and progression. These results are also in line with our previous reports that increased differentiation and matrix deposition are associated with immune reprogramming, toward a less suppressive tumor microenvironment [18, 29].

Our data suggest that ZEB1 contributes to macrophage recruitment and polarization, potentially via regulation of cytokines such as CCL2 and CXCL5, which were indeed down-modulated in ZEB1-deficient cells, as shown in the gene expression profile performed.

In the present study, the analysis of tumor infiltrating T lymphocytes showed only minor changes, and the different tumor growth between ZEB1-proficient and deficient cells seen in immune-competent mice was maintained in immune-deficient mice, suggesting that ZEB1 primarily modulates myeloid rather than T cell compartments and indicating a dominant tumor cell-autonomous role of ZEB1 in OS aggressiveness independent of adaptive immunity.

Metastatic spread to the lung is the major cause of OS mortality [7]. Consistent with reduced primary tumor growth, ZEB1-deficient clones showed impaired lung dissemination, with clone B8 almost completely unable to disseminate. Notably, this phenotype was reversed upon ZEB1 re-expression, confirming its causal role. The down-modulation of invasion-related genes (*Mmp3*, *Mmp13*,* Yap1*) in ZEB1-deficient cells observed in the gene expression analysis provides hints on the molecular basis for this reduced metastatic potential.

Gene expression profiling analysis revealed broad down-regulation of oncogenic pathways, including mTORC1, MYC, E2F targets, glycolysis, and oxidative phosphorylation, all of which sustain cancer aggressiveness. This aligns with the concept that ZEB1, and EMT transcription factors in general, integrate multiple oncogenic pathways to maintain tumor plasticity [30].

Of note, *Sfrp1*, encoding for an inhibitor of Wnt/β-catenin signaling, was one of the mostly upregulated genes in ZEB1-deficient clones, suggesting that ZEB1 represses differentiation-promoting signals. With the idea that SFRP1 forced expression could, at least in part, mimic ZEB1 deletion, we are now over-expressing it in our parental cell lines, which are negative or very low for Sfrp1 expression, to test whether its over-expression will reduce OS aggressiveness similarly to ZEB1 deletion.

Although we observed heterogeneous behavior among the ZEB1 knockout clones, with clone H3 displaying traits more similar to control, this variability highlights the complexity of OS biology and suggests that additional regulatory mechanisms may modulate the effects of ZEB1 loss. Nevertheless, to strengthen our findings, we validated key results in an independent murine OS model (mOS5) and in a patient-derived xenograft (PDX) human cell line using an alternative genetic approach (shRNA interference). Even more importantly, we definitively demonstrated that re-expression of ZEB1 in the knockout B8 clone restored the malignant phenotype, providing direct and compelling evidence that ZEB1 is relevant for osteosarcoma aggressiveness. Interestingly, restoring ZEB1 expression in the ZEB1-deficient clone recreated the immunosuppressive tumor microenvironment of the mOS69 parental line, as evidenced by their comparable levels of CD206 and PDL1 on infiltrating macrophages.

Nevertheless, further studies are needed to dissect the underlying molecular mechanisms by which ZEB1 influences the cellular features we observed to be affected by its deletion or downregulation. We performed an in silico analysis to determine whether ZEB1, as a transcription factor, directly binds the promoter of any genes modulated in ZEB1KO clones. However, none of these genes harbor a ZEB1 DNA-binding site in their promoters, suggesting a more complex regulatory mechanism.

Furthermore, given that OS is a multifactorial malignancy, with no single dominant oncogenic driver and multiple alterations potentially sustaining its aggressive phenotype, ZEB1 likely constitute one of such contributing factors, making the therapeutic targeting of ZEB1 a potentially attractive strategy to be combined with standard of care treatments in those cases with high ZEB1 expression.

## Conclusions

Overall, this work clearly supports the hypothesis of a relevant contribution of ZEB1 in OS aggressiveness by simultaneously promoting stemness and EMT-like traits, repressing osteogenic differentiation, and orchestrating an immune-suppressive microenvironment in vivo.

## Supplementary Information

Supplementary Material 1.

Supplementary Material 2.

Supplementary Material 3.

Supplementary Material 4.

Supplementary Material 5.

## Data Availability

The dataset generated during the current study will be available in the GEO repository, with accession number GSE313626, once the article is published (https://www.ncbi.nlm.nih.gov/geo/query/acc.cgi?acc=GSE313626).
