# Supplementary material for: ZEB1 integrates stemness, differentiation blockade and immune suppression to orchestrate osteosarcoma aggressiveness
Source: J Exp Clin Cancer Res. 2026 Jun 15;45:183. doi: 10.1186/s13046-026-03757-9 (PMC13495327; doi:10.1186/s13046-026-03757-9)
Supplement: Supplementary file 1 [file 13046_2026_3757_MOESM1_ESM.pdf]

Supplementary table 3A. Differentially up-regulated genes ZEB1KO clones B8 and E4 vs parental and SCR G5P1 contrl; logFC >1; FDR< 0.05

PROBEID	ENTREZID	SYMBOL	GENENAME	GENETYPE	logFC	AveExpr	t	P.Value	adj.P.Val	B
TC0800000297.mm.2	20377	Sfrp1	secreted frizzled-related protein 1	protein-coding	5,39864167	7,0031592	21,9801197	3,2982E-15	1,77E-12	24,9343866
TC0400002139.mm.2	18619	Penk	preproenkephalin	protein-coding	5,19374065	9,02253754	23,4844718	9,5077E-16	6,1672E-13	26,1078745
TC0100002645.mm.2	20720	Serpine2	serine (or cysteine) peptidase inhibitor, clade E, member 2	protein-coding	4,53857109	7,93413234	26,7206064	1,9573E-17	2,3012E-14	29,7527479
TC0800001737.mm.2	14456	Gas6	growth arrest specific 6	protein-coding	4,50497871	7,83355735	17,205315	3,0982E-13	8,0944E-11	20,5398272
TC0400002174.mm.2	12319	Car8	carbonic anhydrase 8	protein-coding	4,21000931	7,41599176	20,5028185	1,211E-14	5,1775E-12	23,692209
TC0100001218.mm.2	14264	Fmod	fibromodulin	protein-coding	3,67151237	6,12911436	11,4436674	4,327E-10	2,2673E-08	13,2992292
TC1200001643.mm.2	66109	Tspan13	tetraspanin 13	protein-coding	3,51487279	6,32367696	28,2508108	7,4577E-19	1,5587E-15	32,8429507
TC0X00003113.mm.2	83453	Chrdl1	chordin-like 1	protein-coding	3,46719848	7,25520507	25,4086634	7,2807E-18	9,7826E-15	30,7506455
TC0100002562.mm.2	16011	Igfbp5	insulin-like growth factor binding protein 5	protein-coding	3,42058606	5,91644138	22,7661718	7,5858E-17	7,1704E-14	28,5493919
TC0400000736.mm.2	18491	Pappa	pregnancy-associated plasma protein A	protein-coding	3,34719855	7,57341045	18,1258282	7,2452E-14	2,2715E-11	21,9504882
TC1000000093.mm.2	22634	Plagl1	pleiomorphic adenoma gene-like 1	protein-coding	3,30881273	6,74177668	27,4656289	1,3685E-18	2,1452E-15	32,2905908
TC0300000095.mm.2	12350	Car3	carbonic anhydrase 3	protein-coding	3,28561874	5,80765583	22,1751423	1,3261E-16	1,1339E-13	28,0183296
TC0300000902.mm.2	13038	Ctsk	cathepsin K	protein-coding	3,24424865	7,93042835	15,3034549	2,6071E-12	4,302E-10	18,4332781
TC1000002903.mm.2	14160	Lgr5	leucine rich repeat containing G protein coupled receptor 5	protein-coding	3,22300972	8,44481081	19,8014416	6,8412E-15	3,2172E-12	24,2337832
TC0200000048.mm.2	74186	Ccdc3	coiled-coil domain containing 3	protein-coding	3,13024379	6,13384105	19,9263788	1,5363E-15	9,3223E-13	25,6654484
TC1200002103.mm.2	21809	Tgfb3	transforming growth factor, beta 3	protein-coding	3,12312967	8,54967887	30,0025358	2,0355E-19	7,4429E-16	34,0110937
TC0800000747.mm.2	12845	Comp	cartilage oligomeric matrix protein	protein-coding	3,10823686	6,78563216	11,5254973	3,8313E-10	2,0533E-08	13,4223765
TC1000000402.mm.2	12484	Cd24a	CD24a antigen	protein-coding	3,08430824	7,13795553	11,4689396	4,1671E-10	2,1957E-08	13,3373364
TC1300000566.mm.2	66695	Aspn	asporin	protein-coding	3,00225172	7,445781	10,3695196	2,2764E-09	8,9516E-08	11,6155901
TC1700000842.mm.2	83965	Enpp5	ectonucleotide pyrophosphatase/phosphodiesterase 5	protein-coding	2,97520347	8,81707168	23,9175992	2,6526E-17	2,7722E-14	29,5419198
TC1000003226.mm.2	103140	Gstt3	glutathione S-transferase, theta 3	protein-coding	2,94594108	6,33051133	17,7157156	2,3371E-14	9,1588E-12	23,018029
TC0600000277.mm.2	17294	Mest	mesoderm specific transcript	protein-coding	2,92964705	6,08504705	21,4832553	2,5953E-16	2,097E-13	27,3768925
TC1000001336.mm.2	19263	Ptprb	protein tyrosine phosphatase receptor type B	protein-coding	2,9069404	5,65072095	12,7485173	6,5356E-11	5,4884E-09	15,2061738
TC0X00002267.mm.2	320923	Map7d3	MAP7 domain containing 3	protein-coding	2,90377141	5,87081482	19,1408163	2,9451E-15	1,6294E-12	25,0316744
TC0100002640.mm.2	20254	Scg2	secretogranin II	protein-coding	2,8394118	4,79978039	15,1857754	5,1561E-13	1,2435E-10	19,9605061
TC1600001053.mm.2	20465	Sim2	single-minded family bHLH transcription factor 2	protein-coding	2,79663907	6,09071799	16,4814013	1,0048E-13	3,0001E-11	21,5820053
TC1400000269.mm.2	171463	Il17rd	interleukin 17 receptor D	protein-coding	2,75191033	5,29049164	17,0689943	3,1827E-14	1,1597E-11	22,7012237
TC1900001774.mm.2	381236	Lipo3	lipase, member O3	protein-coding	2,71083665	6,38576793	16,3418288	1,1919E-13	3,4494E-11	21,4131764
TC0500002887.mm.2	13602	Sparcl1	SPARC-like 1	protein-coding	2,7093522	7,18323108	11,7990021	2,5632E-10	1,5162E-08	13,8289578
TC0300001117.mm.2	229699	Slc16a4	solute carrier family 16 (monocarboxylic acid transporters), member 4	protein-coding	2,67848756	6,01117227	12,3275872	9,7068E-11	7,2266E-09	14,7933118
TC1700002817.mm.2	12263	C2	complement C2	protein-coding	2,6641823	5,86456582	15,5938291	3,0428E-13	8,0616E-11	20,4846191
TC0100001510.mm.2	56429	Dpt	dermatopontin	protein-coding	2,66281331	6,9730988	9,61395388	7,8892E-09	2,49E-07	10,3520563
TC1200001638.mm.2	11622	Ahr	aryl-hydrocarbon receptor	protein-coding	2,65561016	7,29227397	18,4206876	7,8433E-15	3,5368E-12	24,0799983
TC0800000017.mm.2	20911	Stxbp2	syntaxin binding protein 2	protein-coding	2,6554827	7,24937344	24,5322001	1,5431E-17	1,9351E-14	30,0501862
TC1000000133.mm.2	64058	Perp	PERP, TP53 apoptosis effector	protein-coding	2,64560293	7,73236868	22,7608427	7,6237E-17	7,1704E-14	28,5446708
TC0X00001270.mm.2	64103	Tnmd	tenomodulin	protein-coding	2,62905468	8,60114449	14,9091286	2,828E-12	4,586E-10	18,3324979
TC1700000168.mm.2	64074	Smoc2	SPARC related modular calcium binding 2	protein-coding	2,61497393	6,82886408	18,6677453	4,9673E-15	2,3959E-12	24,5223825
TC1100002103.mm.2	21452	Tcn2	transcobalamin 2	protein-coding	2,56806397	7,72922586	20,7881631	5,1989E-16	3,7614E-13	26,7099425
TC0400001543.mm.2	14077	Fabp3	fatty acid binding protein 3, muscle and heart	protein-coding	2,52774356	7,96470125	20,6632711	5,9032E-16	4,1128E-13	26,5876188
TC1700000269.mm.2	210503	Zfp677	zinc finger protein 677	protein-coding	2,5208371	5,2452646	15,7431702	1,6711E-13	4,763E-11	21,0618971
TSUnmapped00000030.mm.2	15101	H60a	histocompatibility 60a	protein-coding	2,498995	9,23565685	15,9690715	4,9038E-13	1,2299E-10	20,0515795
TC0500001796.mm.2	68895	Rasl11a	RAS-like, family 11, member A	protein-coding	2,46966557	7,78301521	15,5581374	4,9958E-13	1,2365E-10	20,0122246
TC1500000002.mm.2	20363	Selenop	selenoprotein P	protein-coding	2,44925602	10,0183983	14,7499193	4,913E-12	6,9487E-10	17,8006956
TC1000000193.mm.2	22361	Vnn1	vanin 1	protein-coding	2,43693079	5,20755923	16,1000143	1,0574E-13	3,1078E-11	21,515511
TC1100004123.mm.2	114886	Cygb	cytoglobin	protein-coding	2,42904236	7,77539922	21,4523967	2,6754E-16	2,097E-13	27,3477713
TC1400001177.mm.2	380928	Lmo7	LIM domain only 7	protein-coding	2,40200953	8,66209408	15,7789714	4,1171E-13	1,0609E-10	20,2077089
TC1200001810.mm.2	66864	Clec14a	C-type lectin domain family 14, member a	protein-coding	2,36540357	5,49656993	18,9553173	3,6101E-15	1,8864E-12	24,8334996
TC0800001511.mm.2	13479	Dpep1	dipeptidase 1	protein-coding	2,32833388	6,39996306	13,8841363	3,0142E-12	4,725E-10	18,1985582
TC1500002147.mm.2	69354	Slc38a4	solute carrier family 38, member 4	protein-coding	2,30604709	8,0583712	10,8538281	1,0609E-09	4,7068E-08	12,3904754
TC0700004119.mm.2	50781	Dkk3	dickkopf WNT signaling pathway inhibitor 3	protein-coding	2,29168533	8,29151671	14,0711599	4,708E-12	6,7605E-10	17,7926731
TC1600000184.mm.2	20583	Snai2	snail family zinc finger 2	protein-coding	2,26713048	10,2012849	17,7670204	2,4028E-14	9,2242E-12	22,9931246
TC0600002024.mm.2	243743	Plxna4	plexin A4	protein-coding	2,2665733	4,61987962	14,5334452	8,4383E-13	1,8245E-10	19,4508419
TC1900000161.mm.2	68852	Lrrn4cl	LRRN4 C-terminal like	protein-coding	2,20645742	6,05406983	11,1160279	4,1371E-10	2,1922E-08	13,2984432
TC1000002383.mm.2	14871	Gstt1	glutathione S-transferase, theta 1	protein-coding	2,201808	7,43265578	14,5899194	1,1382E-12	2,2777E-10	19,1716903
TC1000002583.mm.2	216188	Aldh1l2	aldehyde dehydrogenase 1 family, member L2	protein-coding	2,19515575	9,17263749	9,41201795	1,1121E-08	3,2385E-07	10,0026144
TC0300002926.mm.2	13809	Enpep	glutamyl aminopeptidase	protein-coding	2,18619478	4,6990725	16,930769	3,7636E-14	1,3358E-11	22,5360542
TC1600001479.mm.2	11815	Apod	apolipoprotein D	protein-coding	2,18593545	6,3532258	10,251412	2,5783E-09	9,898E-08	11,4828007
TC1400000344.mm.2	239017	Ogdhl	oxoglutarate dehydrogenase-like	protein-coding	2,17643236	5,49954589	13,4448821	3,9931E-12	5,9145E-10	17,8962301
TC1200000797.mm.2	171210	Acot2	acyl-CoA thioesterase 2	protein-coding	2,16607694	8,54994065	12,6839813	4,3045E-11	3,9887E-09	15,5906681
TC0400001881.mm.2	433791	Zfp992	zinc finger protein 992	protein-coding	2,16364649	5,67746946	14,140424	1,4626E-12	2,724E-10	18,9015872
TC0700001432.mm.2	207728	Pde2a	phosphodiesterase 2A, cGMP-stimulated	protein-coding	2,16142172	6,55552922	14,9620056	4,6962E-13	1,1938E-10	20,034973
TC0300001409.mm.2	11522	Adh1	alcohol dehydrogenase 1 (class I)	protein-coding	2,16009007	6,38503084	19,9497437	1,2367E-15	7,7544E-13	25,8736405
TC0700002989.mm.2	14132	Fcgrt	Fc fragment of IgG receptor and transporter	protein-coding	2,14293593	8,2239793	14,487191	1,3082E-12	2,5369E-10	19,0327801
TC0400000952.mm.2	21687	Tek	TEK receptor tyrosine kinase	protein-coding	2,13946071	4,96834408	13,2313704	5,4804E-12	7,4704E-10	17,578723
TC1900000335.mm.2	11668	Aldh1a1	aldehyde dehydrogenase family 1, subfamily A1	protein-coding	2,12767765	5,85970883	17,8169618	1,3107E-14	5,4792E-12	23,5731772
TC0800000482.mm.2	11988	Slc7a2	solute carrier family 7 (cationic amino acid transporter, y+ system), member 2	protein-coding	2,1236721	6,37241804	13,1426131	8,4473E-12	1,0593E-09	17,1633408
TC0400004198.mm.2	666532	Zfp991	zinc finger protein 991	protein-coding	2,11871171	6,70544513	14,1046517	1,7632E-12	3,1289E-10	18,7222258
TC0700001816.mm.2	66090	Ypel3	yippee like 3	protein-coding	2,09167118	7,18974144	13,9144203	2,7001E-12	4,4167E-10	18,3048236
TC0600001440.mm.2	319352	Pianp	PILR alpha associated neural protein	protein-coding	2,0781089	6,46423755	18,2569111	7,8967E-15	3,5368E-12	24,0694945
TC0400002733.mm.2	64817	Svep1	sushi, von Willebrand factor type A, EGF and pentraxin domain containing 1	protein-coding	2,0766104	8,29818708	15,5350246	2,2324E-13	6,1756E-11	20,7752878
TC0900000212.mm.2	12797	Cnn1	calponin 1	protein-coding	2,06844031	9,23633611	10,8103934	1,1349E-09	4,9479E-08	12,3220586
TC1500001112.mm.2	15421	Hoxc12	homeobox C12	protein-coding	2,05002516	7,11627418	10,2028205	2,7904E-09	1,0561E-07	11,4024902
TC0X00003276.mm.2	237178	Ppef1	protein phosphatase with EF hand calcium-binding domain 1	protein-coding	2,03064844	8,21155702	9,97990083	4,2862E-09	1,5E-07	10,972557
TC1200001692.mm.2	664883	Nova1	NOVA alternative splicing regulator 1	protein-coding	2,02967115	7,86692056	14,2238751	1,66E-12	2,9738E-10	18,788329
TC0200000543.mm.2	20204	Prrx2	paired related homeobox 2	protein-coding	2,02854624	8,69573709	10,2101444	2,9429E-09	1,1072E-07	11,3547126
TC0200005052.mm.2	16658	Mafb	MAF bZIP transcription factor B	protein-coding	2,02140596	6,86269498	14,4940573	8,9116E-13	1,8507E-10	19,3963902
TC0400004199.mm.2	433801	Zfp268	zinc finger protein 268	protein-coding	2,01986332	5,73428008	13,100845	6,6639E-12	8,7661E-10	17,382499
TC1000001270.mm.2	114774	Pawr	PRKC, apoptosis, WT1, regulator	protein-coding	2,01439403	5,75028297	10,8071603	4,9552E-10	2,5329E-08	13,0884312
TC0200005136.mm.2	18830	Pltp	phospholipid transfer protein	protein-coding	1,98913102	8,85444682	20,254683	8,9899E-16	6,0396E-13	26,1819667
TC1000002586.mm.2	216190	Appl2	adaptor protein, phosphotyrosine interaction, PH domain and leucine zipper containing 2	protein-coding	1,97821457	7,74636373	17,5684312	1,7537E-14	7,0188E-12	23,2874596
TC0700000917.mm.2	14453	Gas2	growth arrest specific 2	protein-coding	1,97504015	8,34785053	12,0646398	8,3831E-11	6,5706E-09	14,9013631
TC1500000389.mm.2	18133	Ccn3	cellular communication network factor 3	protein-coding	1,96070781	11,1283817	11,0917215	7,3577E-10	3,5039E-08	12,7615124
TC0300001292.mm.2	211550	Tifa	TRAF-interacting protein with forkhead-associated domain	protein-coding	1,95634703	6,54779692	11,3472389	1,9554E-10	1,222E-08	14,025128
TC0800000483.mm.2	68797	Pdgfrl	platelet-derived growth factor receptor-like	protein-coding	1,95250857	8,61707104	11,3546338	3,4282E-10	1,8967E-08	13,5041749
TC0300000647.mm.2	20319	Sfrp2	secreted frizzled-related protein 2	protein-coding	1,94897736	10,9982542	14,6586436	2,8623E-12	4,602E-10	18,3046641
TC1000002929.mm.2	17105	Lyz2	lysozyme 2	protein-coding	1,94646376	4,9812193	11,7727065	5,3346E-11	4,7558E-09	15,2894059
TC1600000834.mm.2	19876	Robo1	roundabout guidance receptor 1	protein-coding	1,932706	5,56024452	11,0119325	2,4857E-10	1,475E-08	13,7571733
TC0400002095.mm.2	74761	Mxra8	matrix-remodelling associated 8	protein-coding	1,93167152	7,97443481	12,3892926	3,6502E-11	3,4855E-09	15,7145088
TC1000001895.mm.2	209558	Enpp3	ectonucleotide pyrophosphatase/phosphodiesterase 3	protein-coding	1,92992434	8,18901233	10,8831624	7,3314E-10	3,5003E-08	12,7363977
TC1600000746.mm.2	320712	Abi3bp	ABI family member 3 binding protein	protein-coding	1,92978732	5,10115753	12,3075191	2,2618E-11	2,377E-09	16,1539689
TC0400001774.mm.2	17150	Mfap2	microfibrillar-associated protein 2	protein-coding	1,92650706	6,52271324	14,4478543	9,5021E-13	1,922E-10	19,3323498
TC1000000365.mm.2	171580	Mical1	microtubule associated monooxygenase, calponin and LIM domain containing 1	protein-coding	1,92579649	6,39568238	15,4030755	2,6063E-13	7,004E-11	20,6206509
TC1000000175.mm.2	667281	H60b	histocompatibility 60b	protein-coding	1,92124411	8,40024875	11,6348343	1,6881E-10	1,0838E-08	14,1980714
TC0700004111.mm.2	233733	Galnt18	polypeptide N-acetylgalactosaminyltransferase 18	protein-coding	1,91925184	6,43240526	13,4168786	4,1615E-12	6,1157E-10	17,8548302
TC1200002078.mm.2	104776	Aldh6a1	aldehyde dehydrogenase family 6, subfamily A1	protein-coding	1,91719293	6,77752347	14,2962034	1,1744E-12	2,3254E-10	19,1208763
TC1400000271.mm.2	71704	Arhgef3	Rho guanine nucleotide exchange factor 3	protein-coding	1,91169654	6,25591034	10,3022356	1,3594E-09	5,708E-08	12,0802724
TC1100000780.mm.2	76293	Mfap4	microfibrillar-associated protein 4	protein-coding	1,91164634	6,16738705	11,6738071	7,9351E-11	6,3249E-09	14,9065833
TC0800002403.mm.2	65972	Ifi30	interferon gamma inducible protein 30	protein-coding	1,91075847	8,5337182	16,5923496	5,702E-14	1,9502E-11	22,1261671
TC0800000983.mm.2	17159	Man2b1	mannosidase 2, alpha B1	protein-coding	1,90716976	7,11821215	19,5226866	1,9474E-15	1,1353E-12	25,4335808
TC1300001326.mm.2	13723	Emb	embigin	protein-coding	1,9071478	9,3403367	7,8191707	1,9941E-07	3,5861E-06	7,06190645
TC0200001552.mm.2	12509	Cd59a	CD59a antigen	protein-coding	1,90627769	6,58274599	9,87195357	3,4875E-09	1,2689E-07	11,1439249
TC0600003335.mm.2	17313	Mgp	matrix Gla protein	protein-coding	1,90234977	9,90406603	8,53248897	5,2595E-08	1,1425E-06	8,42014327
TC1000000349.mm.2	78334	Cdk19	cyclin dependent kinase 19	protein-coding	1,89455045	7,72943052	18,6702742	4,9533E-15	2,3959E-12	24,5251394
TC1000002405.mm.2	12834	Col6a2	collagen, type VI, alpha 2	protein-coding	1,88692868	9,42459336	14,6779747	8,905E-13	1,8507E-10	19,4099754
TC0300002802.mm.2	22329	Vcam1	vascular cell adhesion molecule 1	protein-coding	1,88374752	8,11362998	11,4498839	2,0931E-10	1,2825E-08	13,9749464
TC0400002216.mm.2	381511	Pdp1	pyruvate dehydrogenase phosphatase catalytic subunit 1	protein-coding	1,88304712	8,13226225	10,4983378	1,4138E-09	5,8969E-08	12,074002
TC1100004277.mm.2	54409	Ramp2	receptor (calcitonin) activity modifying protein 2	protein-coding	1,87098325	5,42510992	9,29235191	7,987E-09	2,5082E-07	10,282641

TC0400003742.mm.2	230815	Man1c1	mannosidase, alpha, class 1C, member 1	protein-coding	1,86852284	6,28450677	13,6572317	2,9259E-12	4,6644E-10	18,2078053
TC0700002047.mm.2	98845	Eps8l2	EPS8-like 2	protein-coding	1,86745609	4,79230636	10,7567215	2,99E-10	1,7044E-08	13,5495055
TC0900000989.mm.2	11891	Rab27a	RAB27A, member RAS oncogene family	protein-coding	1,86645313	5,91533353	10,0777313	1,7897E-09	7,3188E-08	11,7911524
TC0200000933.mm.2	227960	Gca	grancalcin	protein-coding	1,85570715	5,78865132	8,9716826	1,7124E-08	4,6214E-07	9,52631612
TC1500001968.mm.2	239559	A4galt	alpha 1,4-galactosyltransferase	protein-coding	1,84878549	6,8769968	10,3599613	1,265E-09	5,3959E-08	12,15562
TC0500001682.mm.2	243312	Elfn1	leucine rich repeat and fibronectin type III, extracellular 1	protein-coding	1,84714223	6,88421975	9,46903562	8,0788E-09	2,5244E-07	10,304654
TC0600003598.mm.2	93674	Nat8f3	N-acetyltransferase 8 (GCN5-related) family member 3	protein-coding	1,83466182	5,93428249	14,4807074	9,0782E-13	1,8562E-10	19,3779049
TC0600002996.mm.2	19713	Ret	ret proto-oncogene	protein-coding	1,8265235	6,02115205	14,7595888	6,1829E-13	1,4359E-10	19,7609641
TC1400000316.mm.2	66175	Mustn1	musculoskeletal, embryonic nuclear protein 1	protein-coding	1,82641278	7,56499638	8,4404766	6,2228E-08	1,3108E-06	8,24876514
TC0600001807.mm.2	269823	Pon3	paraoxonase 3	protein-coding	1,81843105	9,32272015	11,0589922	5,6985E-10	2,8283E-08	12,9938649
TC0600000948.mm.2	26903	Dysf	dysferlin	protein-coding	1,8147384	6,07279276	16,1877307	9,461E-14	2,8705E-11	21,6255806
TC1900000046.mm.2	56464	Ctsf	cathepsin F	protein-coding	1,80882079	5,75428304	11,3101226	1,1619E-10	8,1551E-09	14,5048385
TC0500000699.mm.2	68939	Rasl11b	RAS-like, family 11, member B	protein-coding	1,8058762	4,5718621	12,2157134	2,6155E-11	2,6265E-09	16,0076961
TC1700002438.mm.2	19274	Ptprm	protein tyrosine phosphatase receptor type M	protein-coding	1,80440537	6,81921615	13,6257982	3,063E-12	4,7619E-10	18,1619448
TC1600001419.mm.2	12737	Cldn1	claudin 1	protein-coding	1,80217575	5,18164109	13,1908506	5,8224E-12	7,8475E-10	17,5179818
TC0600001422.mm.2	50909	C1ra	complement component 1, r subcomponent A	protein-coding	1,80185008	8,70255849	11,6162456	1,6082E-10	1,0504E-08	14,2409557
TC0100002603.mm.2	74577	Glb1l	galactosidase, beta 1-like	protein-coding	1,79945052	5,6670231	12,1869689	2,7377E-11	2,6963E-09	15,9617181
TC1100000009.mm.2	216505	Pik3ip1	phosphoinositide-3-kinase interacting protein 1	protein-coding	1,79428531	7,64280691	10,9580098	4,3213E-10	2,2673E-08	13,2365077
TC0600000849.mm.2	232086	Tmem150a	transmembrane protein 150A	protein-coding	1,78476393	8,03557895	11,483111	1,4916E-10	9,8798E-09	14,2944724
TC0300002245.mm.2	229474	Fhdc1	FH2 domain containing 1	protein-coding	1,77726835	7,31943673	14,6596254	7,0906E-13	1,5801E-10	19,6244039
TC0700001280.mm.2	83671	Sytl2	synaptotagmin-like 2	protein-coding	1,75656797	6,23637433	12,3469576	2,1255E-11	2,2825E-09	16,2165383
TC1200000211.mm.2	77480	Kidins220	kinase D-interacting substrate 220	protein-coding	1,75243914	7,84734128	17,1940886	2,7375E-14	1,0299E-11	22,8495988
TC1700001571.mm.2	240038	Zfp994	zinc finger protein 994	protein-coding	1,74469248	5,90875983	8,56114858	3,6179E-08	8,4647E-07	8,7640015
TC0400000084.mm.2	60599	Trp53inp1	transformation related protein 53 inducible nuclear protein 1	protein-coding	1,73593419	4,36285401	11,2677302	1,2319E-10	8,5512E-09	14,4446904
TC0400002665.mm.2	11303	Abca1	ATP-binding cassette, sub-family A member 1	protein-coding	1,7358773	6,34145897	13,8991954	2,0626E-12	3,6261E-10	18,557826
TC1900000748.mm.2	27360	Add3	adducin 3	protein-coding	1,73469342	7,89623419	14,1668858	1,4089E-12	2,677E-10	18,9389855
TC0900001635.mm.2	20623	Snrk	SNF related kinase	protein-coding	1,72081319	6,53297656	9,53141816	5,1897E-09	1,7376E-07	10,7205393
TC1100001236.mm.2	327978	Slfn5	schlafen 5	protein-coding	1,71738408	5,54228577	9,09711525	9,7604E-09	2,9283E-07	10,0624566
TC1100001592.mm.2	16010	Igfbp4	insulin-like growth factor binding protein 4	protein-coding	1,71109754	9,34678626	13,5699863	3,7857E-12	5,6519E-10	17,9574176
TC0100000338.mm.2	70676	Gulp1	GULP, engulfment adaptor PTB domain containing 1	protein-coding	1,70920304	8,67757637	9,96994582	3,2031E-09	1,1955E-07	11,2383035
TC0700003570.mm.2	16790	Anpep	alanyl aminopeptidase, membrane	protein-coding	1,70804793	7,1470685	11,96705	3,9464E-11	3,6751E-09	15,5942643
TC1000003103.mm.2	52666	Arhgef25	Rho guanine nucleotide exchange factor 25	protein-coding	1,70779352	8,34595642	15,4943214	2,3115E-13	6,3017E-11	20,7399057
TC0600000182.mm.2	214642	Cped1	cadherin-like and PC-esterase domain containing 1	protein-coding	1,70591275	7,80426995	12,355631	2,1981E-11	2,3361E-09	16,1860157
TC1800001449.mm.2	667742	Piezo2	piezo-type mechanosensitive ion channel component 2	protein-coding	1,69930536	6,43107516	11,1446548	1,6662E-10	1,0734E-08	14,147174
TC0200000781.mm.2	11480	Acvr2a	activin receptor IIA	protein-coding	1,69008184	7,4953025	11,7080983	6,8784E-11	5,7E-09	15,0441623
TC1700001197.mm.2	381110	Rmdn2	regulator of microtubule dynamics 2	protein-coding	1,6823002	7,79213384	8,99451025	1,9499E-08	5,1518E-07	9,41281123
TC0300000949.mm.2	56338	Txnip	thioredoxin interacting protein	protein-coding	1,67011167	9,04290236	12,0041459	4,9485E-11	4,4753E-09	15,386979
TC1800001463.mm.2	54670	Atp8b1	ATPase, class I, type 8B, member 1	protein-coding	1,66525398	6,31440427	13,0082387	7,6627E-12	9,7393E-10	17,2422899
TC0300002056.mm.2	242050	Igsf10	immunoglobulin superfamily, member 10	protein-coding	1,66337261	6,4639026	13,0246759	7,4746E-12	9,6969E-10	17,2672368
TC1100003345.mm.2	338367	Myo1d	myosin ID	protein-coding	1,66250095	8,41974509	14,2593189	1,2368E-12	2,4235E-10	19,0691439
TC1900000552.mm.2	74055	Plce1	phospholipase C, epsilon 1	protein-coding	1,63814125	7,88951833	17,0630516	3,2057E-14	1,1597E-11	22,6941489
TC0500003576.mm.2	66898	Baiap2l1	BAI1-associated protein 2-like 1	protein-coding	1,63519271	5,77205381	14,4907142	8,953E-13	1,8507E-10	19,3917624
TC1000002464.mm.2	72128	2610008E11Rik	RIKEN cDNA 2610008E11 gene	protein-coding	1,63515031	7,70402317	10,6015429	5,1463E-10	2,6094E-08	13,0234149
TC1200001988.mm.2	12192	Zfp36l1	zinc finger protein 36, C3H type-like 1	protein-coding	1,6245884	9,09699647	14,5900199	7,8038E-13	1,7069E-10	19,5288256
TC1400002719.mm.2	66573	Dzip1	DAZ interacting protein 1	protein-coding	1,6232615	7,35964527	8,37091534	6,2383E-08	1,3112E-06	8,23128771
TC0700000566.mm.2	243912	Hspb6	heat shock protein, alpha-crystallin-related, B6	protein-coding	1,62082471	8,23052639	13,0084091	7,6607E-12	9,7393E-10	17,2425485
TC1600000266.mm.2	75785	Klhl24	kelch-like 24	protein-coding	1,61908989	8,44613964	9,77881799	3,6044E-09	1,3039E-07	11,0978842
TC1100004229.mm.2	12409	Cbr2	carbonyl reductase 2	protein-coding	1,61856743	7,36870335	9,53124865	5,1912E-09	1,7376E-07	10,7202321
TC1400001346.mm.2	223272	Itgbl1	integrin, beta-like 1	protein-coding	1,61542582	6,47759134	8,5164919	3,6053E-08	8,4457E-07	8,75755084
TC0300002629.mm.2	99543	Olfml3	olfactomedin-like 3	protein-coding	1,61528264	10,1134507	10,9581776	5,5669E-10	2,7837E-08	13,0017576
TC0900002387.mm.2	26968	Islr	immunoglobulin superfamily containing leucine-rich repeat	protein-coding	1,6097551	6,68762861	9,05066524	1,2409E-08	3,5636E-07	9,83475563
TC1200001956.mm.2	104886	Rab15	RAB15, member RAS oncogene family	protein-coding	1,60876182	4,6644148	11,6087779	6,9796E-11	5,7216E-09	15,0182734
TC1200002416.mm.2	21981	Ppp1r13b	protein phosphatase 1, regulatory subunit 13B	protein-coding	1,60619838	6,28402278	12,3215209	2,2124E-11	2,3381E-09	16,1762011
TC1000001020.mm.2	216198	Tcp11l2	t-complex 11 (mouse) like 2	protein-coding	1,60490509	5,88601277	9,64864809	2,5853E-09	9,9046E-08	11,3801866
TC1000003097.mm.2	216440	Os9	amplified in osteosarcoma	protein-coding	1,59823045	9,32277316	16,5754729	5,8225E-14	1,9558E-11	22,105519
TC1400000368.mm.2	11752	Anxa8	annexin A8	protein-coding	1,59714605	5,95474104	10,1018909	9,8104E-10	4,4149E-08	12,349147
TC1000002093.mm.2	11630	Crybg1	crystallin beta-gamma domain containing 1	protein-coding	1,59331532	5,80621552	12,1070846	3,1094E-11	3,015E-09	15,8334863
TC0600002574.mm.2	11468	Actg2	actin, gamma 2, smooth muscle, enteric	protein-coding	1,59206326	5,78061291	6,89471723	1,2409E-06	1,6928E-05	5,19948562
TC1100003159.mm.2	83429	Ctns	cystinosis, nephropathic	protein-coding	1,57738288	8,30625704	10,8959088	3,0225E-10	1,7177E-08	13,5594212
TC0800000390.mm.2	104271	Tex15	testis expressed gene 15 meiosis and synapsis associated	protein-coding	1,57657783	5,32877078	10,6349995	3,6548E-10	1,9985E-08	13,3454387
TC1700001570.mm.2	210853	Zfp947	zinc finger protein 947	protein-coding	1,57053385	5,3998563	10,414335	5,397E-10	2,7073E-08	12,9509886
TC0100000103.mm.2	17087	Ly96	lymphocyte antigen 96	protein-coding	1,56806358	7,43807205	5,50529749	2,3933E-05	0,00020814	2,19458594
TC1000000794.mm.2	20513	Slc1a6	solute carrier family 1 (high affinity aspartate/glutamate transporter), member 6	protein-coding	1,56688956	6,30250467	7,23058114	6,2334E-07	9,3431E-06	5,89908581
TC0X00001557.mm.2	245671	Klf8	Kruppel-like transcription factor 8	protein-coding	1,56504039	5,13509359	13,009858	7,6439E-12	9,7393E-10	17,2447486
TC1700001852.mm.2	240064	Zfp799	zinc finger protein 799	protein-coding	1,55746011	5,49479154	6,12853408	6,1576E-06	6,522E-05	3,5706784
TC0500000860.mm.2	231440	Parm1	prostate androgen-regulated mucin-like protein 1	protein-coding	1,55743505	6,11176648	9,16538124	6,7114E-09	2,1544E-07	10,417311
TC1100001651.mm.2	11754	Aoc3	amine oxidase, copper containing 3	protein-coding	1,55741453	5,9059863	11,408327	9,7323E-11	7,2266E-09	14,6827209
TC0200004507.mm.2	213696	Duoxa1	dual oxidase maturation factor 1	protein-coding	1,55589386	8,08280978	12,2492654	2,48E-11	2,5354E-09	16,0612553
TC1000002406.mm.2	12833	Col6a1	collagen, type VI, alpha 1	protein-coding	1,55368474	9,4518959	13,3866707	4,3514E-12	6,2964E-10	17,8100894
TC0800001414.mm.2	12554	Cdh13	cadherin 13	protein-coding	1,54694191	7,06290701	10,4911288	5,1723E-10	2,6155E-08	13,0021327
TC0400001790.mm.2	29818	Hspb7	heat shock protein family, member 7 (cardiovascular)	protein-coding	1,54266683	6,54476706	12,0994175	3,1477E-11	3,0365E-09	15,8211437
TC1700002346.mm.2	66972	Slc25a23	solute carrier family 25 (mitochondrial carrier; phosphate carrier), member 23	protein-coding	1,5421727	6,66972882	10,2216658	8,3407E-10	3,8932E-08	12,5183177
TC1100003659.mm.2	72324	Plxdc1	plexin domain containing 1	protein-coding	1,5421143	6,18957955	11,4377972	9,2656E-11	7,0565E-09	14,7323332
TC1700001270.mm.2	73582	Camkmt	calmodulin-lysine N-methyltransferase	protein-coding	1,53980446	7,08532471	8,25136123	6,7529E-08	1,4021E-06	8,13321461
TC1000000674.mm.2	66859	Slc16a9	solute carrier family 16 (monocarboxylic acid transporters), member 9	protein-coding	1,53820153	6,4137213	11,2201344	1,3349E-10	9,0982E-09	14,3636058
TC0700003451.mm.2	233335	Synm	synemin, intermediate filament protein	protein-coding	1,53681495	5,14128379	8,79462528	1,2558E-08	3,5846E-07	9,77223372
TC0X00001555.mm.2	245670	Rragb	Ras-related GTP binding B	protein-coding	1,53268845	7,25228297	11,4135194	9,6483E-11	7,2266E-09	14,6914693
TC0500002280.mm.2	18196	Nsg1	neuron specific gene family member 1	protein-coding	1,5270688	8,8955117	9,01717226	1,6923E-08	4,5804E-07	9,5459833
TC1000001868.mm.2	14051	Eya4	EYA transcriptional coactivator and phosphatase 4	protein-coding	1,52533901	8,45639483	10,6243074	4,8047E-10	2,4762E-08	13,0903941
TC1700000402.mm.2	60455	Pgap6	post-glycosylphosphatidylinositol attachment to proteins 6	protein-coding	1,52266984	5,79097286	10,6168512	3,773E-10	2,0384E-08	13,3132177
TC1400001613.mm.2	66039	Tmem254	transmembrane protein 254	protein-coding	1,52126083	8,43077984	12,3779967	2,0243E-11	2,2399E-09	16,2656696
TC0200002270.mm.2	11602	Angpt4	angiopoietin 4	protein-coding	1,51928842	7,82806236	10,4224177	6,5094E-10	3,1722E-08	12,7788955
TC1000003032.mm.2	320183	Msrb3	methionine sulfoxide reductase B3	protein-coding	1,51727072	9,92006857	16,3826482	7,4032E-14	2,283E-11	21,8681821
TC1700000694.mm.2	386463	Cdsn	corneodesmosin	protein-coding	1,51477437	7,62542507	9,06690322	1,2043E-08	3,4799E-07	9,8651601
TC1200001393.mm.2	238057	Gdf7	growth differentiation factor 7	protein-coding	1,51136998	8,05653865	9,55377292	4,395E-09	1,5142E-07	10,8767132
TC1000002592.mm.2	77976	Nuak1	NUAK family, SNF1-like kinase, 1	protein-coding	1,50846825	8,11012727	11,959657	3,9394E-11	3,6751E-09	15,5950691
TC0300000408.mm.2	50706	Postn	periostin, osteoblast specific factor	protein-coding	1,50758711	9,72024417	7,21792229	6,4632E-07	9,6336E-06	5,86370984
TC1300002481.mm.2	15212	Hexb	hexosaminidase B	protein-coding	1,50678533	8,84891103	10,4324766	6,8949E-10	3,3171E-08	12,7272511
TC1900000763.mm.2	18569	Pdcd4	programmed cell death 4	protein-coding	1,5066691	8,84479102	7,89264349	1,7329E-07	3,171E-06	7,20496975
TC0600003108.mm.2	50908	C1s1	complement component 1, s subcomponent 1	protein-coding	1,49965338	7,98539106	10,3165212	7,999E-10	3,7711E-08	12,5719862
TC0800003161.mm.2	11459	Acta1	actin alpha 1, skeletal muscle	protein-coding	1,4995869	6,11440336	12,3439734	2,1355E-11	2,2825E-09	16,2118095
TC1800001139.mm.2	27528	Nrep	neuronal regeneration related protein	protein-coding	1,49630465	7,44514241	8,2282737	6,8787E-08	1,4219E-06	8,11137159
TC0600001424.mm.2	667277	C1rb	complement component 1, r subcomponent B	protein-coding	1,4946812	7,72450687	9,63477365	2,9645E-09	1,1131E-07	11,2521147
TC0400003043.mm.2	15569	Elavl2	ELAV like RNA binding protein 1	protein-coding	1,48787125	5,21965897	8,34451195	3,2166E-08	7,6249E-07	8,82259089
TC0600002326.mm.2	21743	Inmt	indolethylamine N-methyltransferase	protein-coding	1,48721135	5,32363876	9,18496892	5,2645E-09	1,7559E-07	10,6429015
TC1000000372.mm.2	140742	Sesn1	sestrin 1	protein-coding	1,47995026	7,8691585	10,0495035	1,3173E-09	5,5811E-08	12,0688049
TC1100003948.mm.2	71795	Pitpnc1	phosphatidylinositol transfer protein, cytoplasmic 1	protein-coding	1,47864998	6,50019996	12,2328724	2,5453E-11	2,5794E-09	16,0351017
TC1700002660.mm.2	213760	Prepl	prolyl endopeptidase-like	protein-coding	1,47783074	7,85554892	9,73520524	2,3244E-09	9,0716E-08	11,4930715
TC1200002438.mm.2	100041194	Ahnak2	AHNAK nucleoprotein 2	protein-coding	1,46810792	8,29345079	13,6399891	3,0003E-12	4,725E-10	18,18266
TC1000003019.mm.2	73914	Irak3	interleukin-1 receptor-associated kinase 3	protein-coding	1,46739687	8,26676165	10,9304088	2,2144E-10	1,3351E-08	13,8531513
TC0600002079.mm.2	19242	Ptn	pleiotrophin	protein-coding	1,46376564	9,21752318	6,49534864	2,8325E-06	3,3809E-05	4,3597393
TC1000002418.mm.2	12822	Col18a1	collagen, type XVIII, alpha 1	protein-coding	1,45919286	6,27939928	9,09124977	7,3679E-09	2,3451E-07	10,3176971
TC1400002031.mm.2	76338	Rab2b	RAB2B, member RAS oncogene family	protein-coding	1,45909929	6,85530627	8,14390144	7,0825E-08	1,4608E-06	8,06571508
TC1600001824.mm.2	106338	Nsun3	NOL1/NOP2/Sun domain family member 3	protein-coding	1,45873151	6,18910733	5,75100931	1,3937E-05	0,00013174	2,74205426
TC1100003462.mm.2	77864	Ypel2	yippee like 2	protein-coding	1,45822143	6,11497173	10,9930481	1,9645E-10	1,2237E-08	13,9731819
TC1600001667.mm.2	208177	Phldb2	pleckstrin homology like domain, family B, member 2	protein-coding	1,45683972	8,78777701	10,3157928	8,2169E-10	3,8546E-08	12,5470419
TC0200001006.mm.2	241452	Dhrs9	dehydrogenase/reductase 9	protein-coding	1,44885729	8,76233883	11,3739366	1,0308E-10	7,4292E-09	14,6247029
TC1600000667.mm.2	67896	Ccdc80	coiled-coil domain containing 80	protein-coding	1,44796335	8,14004641	11,5165252	8,1293E-11	6,4357E-09	14,864396
TC1500001457.mm.2	239405	Rspo2	R-spondin 2	protein-coding	1,44658426	9,86421736	10,1381937	1,7974E-09	7,3342E-08	11,7969787
TC1200001028.mm.2	68149	Otub2	OTU domain, ubiquitin aldehyde binding 2	protein-coding	1,44533364	6,37645602	11,7961151	5,1348E-11	4,6216E-09	15,3278846
TC1000002234.mm.2	71279	Slc29a3	solute carrier family 29 (nucleoside transporters), member 3	protein-coding	1,44532341	8,92226861	13,1887796	5,8405E-12	7,8475E-10	17,514873
TC0700001368.mm.2	434215	Lrrc32	leucine rich repeat containing 32	protein-coding	1,44279717	8,64422171	8,90450541	1,7072E-08	4,6141E-07	9,51565244

TC1000000736.mm.2	20203	S100b	S100 protein, beta polypeptide, neural	protein-coding	1,44086944	3,88858466	11,1294999	1,5564E-10	1,0201E-08	14,2084883
TC0200005467.mm.2	227696	Phyhd1	phytanoyl-CoA dioxygenase domain containing 1	protein-coding	1,43881361	7,224088	12,5186104	1,6246E-11	1,8634E-09	16,4870116
TC1600001262.mm.2	224014	Fgd4	FYVE, RhoGEF and PH domain containing 4	protein-coding	1,43861043	6,20813699	12,5555205	1,5339E-11	1,7811E-09	16,5447796
TC0200002426.mm.2	16803	Lbp	lipopolysaccharide binding protein	protein-coding	1,43829852	5,84641695	9,26589041	4,5056E-09	1,5466E-07	10,8007657
TC1400000958.mm.2	13592	Ebf2	early B cell factor 2	protein-coding	1,43691358	7,45587791	13,0684665	6,9967E-12	9,14E-10	17,3335707
TC1000002320.mm.2	71371	Arid5b	AT-rich interaction domain 5B	protein-coding	1,43671528	9,59251359	10,7513261	3,8644E-10	2,0652E-08	13,3108151
TC1800000160.mm.2	67664	Rnf125	ring finger protein 125	protein-coding	1,43496681	6,72415624	11,698482	6,0229E-11	5,2696E-09	15,1670037
TC0900001056.mm.2	235505	Cd109	CD109 antigen	protein-coding	1,43480446	8,12865561	12,2128315	2,6275E-11	2,6265E-09	16,0030903
TC0X00001682.mm.2	14758	Gpm6b	glycoprotein m6b	protein-coding	1,43358133	6,64939228	11,3669735	1,0429E-10	7,4876E-09	14,6129398
TC0800003224.mm.2	70546	Zdhhc2	zinc finger, DHHC domain containing 2	protein-coding	1,43188487	6,67975929	9,32104998	4,6463E-09	1,592E-07	10,7831003
TC1200002296.mm.2	94040	Clmn	calmin	protein-coding	1,43110491	4,52032804	8,3616839	2,6913E-08	6,6486E-07	8,98755867
TC0800000707.mm.2	16956	Lpl	lipoprotein lipase	protein-coding	1,43010334	10,1434168	11,5790952	9,7579E-11	7,2266E-09	14,7019735
TC1000001286.mm.2	237542	Osbpl8	oxysterol binding protein-like 8	protein-coding	1,42284591	6,98088984	9,59475121	2,621E-09	1,0021E-07	11,3580006
TC0700003608.mm.2	12877	Cpeb1	cytoplasmic polyadenylation element binding protein 1	protein-coding	1,4173335	6,64582066	11,1997292	1,3817E-10	9,2687E-09	14,3287646
TC0100002690.mm.2	98496	Pid1	phosphotyrosine interaction domain containing 1	protein-coding	1,41022338	5,78284788	9,10006131	6,2041E-09	2,0191E-07	10,4763389
TC1000000778.mm.2	50723	Icosl	icos ligand	protein-coding	1,40891784	6,14092797	12,4117069	1,92E-11	2,1498E-09	16,3189171
TC1700002420.mm.2	106585	Ankrd12	ankyrin repeat domain 12	protein-coding	1,40637703	8,21193876	13,8831055	2,1108E-12	3,6765E-10	18,5347139
TC0100001877.mm.2	18845	Plxna2	plexin A2	protein-coding	1,40353067	5,76196792	11,107135	1,6168E-10	1,0524E-08	14,1700673
TC0700002669.mm.2	18807	Pld3	phospholipase D family member 3	protein-coding	1,40173514	8,76790815	14,151609	1,4396E-12	2,7081E-10	18,9174023
TC0200002272.mm.2	69698	Slc52a3	solute carrier protein family 52, member 3	protein-coding	1,39911677	5,8775944	9,84608093	1,5118E-09	6,2776E-08	11,9078294
TC0100002789.mm.2	12835	Col6a3	collagen, type VI, alpha 3	protein-coding	1,39721178	9,53054972	12,4974924	1,6789E-11	1,9141E-09	16,4538979
TC0X00002710.mm.2	11989	Slc7a3	solute carrier family 7 (cationic amino acid transporter, y+ system), member 3	protein-coding	1,39628322	8,73644984	3,4393691	0,00354693	0,01489979	-2,70253134
TC1700002313.mm.2	57435	Plin4	perilipin 4	protein-coding	1,39526561	7,12526557	12,156461	2,8739E-11	2,8156E-09	15,912825
TC0400001160.mm.2	69908	Rab3b	RAB3B, member RAS oncogene family	protein-coding	1,39212506	6,87389747	10,6824667	3,3632E-10	1,8718E-08	13,4295279
TC0200003146.mm.2	83768	Dpp7	dipeptidylpeptidase 7	protein-coding	1,39140122	9,3619866	12,1209669	3,0412E-11	2,9641E-09	15,8558183
TC0200002308.mm.2	15162	Hck	hemopoietic cell kinase	protein-coding	1,39042968	3,79591809	8,69044008	1,388E-08	3,9002E-07	9,65940783
TC1100003222.mm.2	20317	Serpinf1	serine (or cysteine) peptidase inhibitor, clade F, member 1	protein-coding	1,38953042	10,4252113	14,7048623	6,6638E-13	1,5103E-10	19,6863046
TC1100000880.mm.2	77579	Myh10	myosin, heavy polypeptide 10, non-muscle	protein-coding	1,3865191	9,742554	13,7805042	2,447E-12	4,1846E-10	18,3867888
TC0500002254.mm.2	81840	Sorcs2	sortilin-related VPS10 domain containing receptor 2	protein-coding	1,38172402	4,76244879	8,79299123	1,1323E-08	3,287E-07	9,86602242
TC0X00003075.mm.2	14605	Tsc22d3	TSC22 domain family, member 3	protein-coding	1,38071981	7,01260825	8,92490634	1,0628E-08	3,1337E-07	9,9506828
TC1200002077.mm.2	12499	Entpd5	ectonucleoside triphosphate diphosphohydrolase 5	protein-coding	1,37716852	6,70039208	10,1656405	8,431E-10	3,9256E-08	12,4993619
TC0400003097.mm.2	13110	Cyp2j6	cytochrome P450, family 2, subfamily j, polypeptide 6	protein-coding	1,37653178	6,58387214	10,6138945	3,7927E-10	2,0384E-08	13,3079646
TC0X00003168.mm.2	80884	Maged2	MAGE family member D2	protein-coding	1,37546297	9,23181548	8,79867194	2,1046E-08	5,4455E-07	9,30423053
TC1000000919.mm.2	216177	AU041133	expressed sequence AU041133	protein-coding	1,37520314	5,0847746	7,56856187	1,6222E-07	2,9916E-06	7,18229577
TC1000002392.mm.2	17385	Mmp11	matrix metallopeptidase 11	protein-coding	1,37448361	7,23607313	10,0485147	1,0428E-09	4,6375E-08	12,2840288
TC1200002242.mm.2	23876	Fbln5	fibulin 5	protein-coding	1,36502619	9,13780097	14,1119615	1,5227E-12	2,781E-10	18,8612931
TC0200001627.mm.2	107515	Lgr4	leucine-rich repeat-containing G protein-coupled receptor 4	protein-coding	1,3648835	9,15841759	12,2970322	2,2996E-11	2,4032E-09	16,1373044
TC1700001599.mm.2	56863	Cldn9	claudin 9	protein-coding	1,36345743	6,09453592	9,16512474	5,47E-09	1,8116E-07	10,604058
TC0800001320.mm.2	244654	Mtss2	MTSS I-BAR domain containing 2	protein-coding	1,35995681	7,39179189	9,67000275	2,1181E-09	8,4056E-08	11,5670405
TC0X00001633.mm.2	237175	Adgrg2	adhesion G protein-coupled receptor G2	protein-coding	1,35726024	6,69401883	8,2517508	4,0865E-08	9,329E-07	8,58552183
TC0600001384.mm.2	50530	Mfap5	microfibrillar associated protein 5	protein-coding	1,35530504	9,51073131	10,5258565	5,3685E-10	2,7002E-08	12,9729302
TC0600000577.mm.2	93695	Gpnmb	glycoprotein (transmembrane) nmb	protein-coding	1,35407723	10,7634552	16,5438796	6,0552E-14	1,9639E-11	22,0668119
TC1000002248.mm.2	404634	Macroh2a2	macroH2A.2 histone	protein-coding	1,35307546	6,71837289	8,36377171	3,1985E-08	7,6162E-07	8,83201338
TC0700004652.mm.2	20646	Snrpn	small nuclear ribonucleoprotein N	protein-coding	1,35263362	5,46682269	9,41231801	3,4068E-09	1,2625E-07	11,0842524
TC0100001570.mm.2	320078	Olfml2b	olfactomedin-like 2B	protein-coding	1,35188283	7,84670769	8,10122447	7,5662E-08	1,5454E-06	7,99642668
TC0100001577.mm.2	75472	Cfap126	cilia and flagella associated protein 126	protein-coding	1,35134834	5,26831945	8,10707096	4,5737E-08	1,0279E-06	8,4503258
TC0500002266.mm.2	17160	Man2b2	mannosidase 2, alpha B2	protein-coding	1,35091445	7,6818199	12,636451	1,353E-11	1,5907E-09	16,6709639
TC0600002315.mm.2	231997	Fkbp14	FK506 binding protein 14	protein-coding	1,35084361	8,39942417	6,56316979	2,4584E-06	3,0029E-05	4,50379632
TC1700000839.mm.2	53901	Rcan2	regulator of calcineurin 2	protein-coding	1,34533412	8,91270457	12,0473669	3,4212E-11	3,2835E-09	15,7371888
TC0900001513.mm.2	100504715	Tmppe	transmembrane protein with metallophosphoesterase domain	protein-coding	1,3453157	8,97431312	11,7226329	5,7893E-11	5,1128E-09	15,2068961
TC0200005164.mm.2	72043	Sulf2	sulfatase 2	protein-coding	1,34384282	7,02953641	7,70578172	1,6638E-07	3,0564E-06	7,19509035
TC1500000086.mm.2	81799	C1qtnf3	C1q and tumor necrosis factor related protein 3	protein-coding	1,3421346	4,81766509	8,1947748	3,7872E-08	8,7628E-07	8,64090213
TC0300000750.mm.2	14466	Gba1	glucosylceramidase beta 1	protein-coding	1,34157866	9,41354753	11,0055631	1,9228E-10	1,2057E-08	13,9948529
TC0600003109.mm.2	317677	C1s2	complement component 1, s subcomponent 2	protein-coding	1,33651306	6,4319886	11,390719	1,0023E-10	7,3361E-09	14,653032
TC0400001382.mm.2	19063	Ppt1	palmitoyl-protein thioesterase 1	protein-coding	1,33635232	8,38141549	9,75948928	1,9311E-09	7,7453E-08	11,667687
TC1000000509.mm.2	57319	Smpdl3a	sphingomyelin phosphodiesterase, acid-like 3A	protein-coding	1,33290689	6,64581401	8,27428745	3,8275E-08	8,8342E-07	8,64972052
TC1800001026.mm.2	12558	Cdh2	cadherin 2	protein-coding	1,33260234	8,73419764	9,52048491	3,4498E-09	1,2625E-07	11,0925955
TC1700001390.mm.2	22350	Ezr	ezrin	protein-coding	1,33239052	8,82093775	12,6813543	1,2624E-11	1,4935E-09	16,7406923
TC1000002553.mm.2	331188	Zfp781b	zinc finger protein 781B	protein-coding	1,3275075	6,52396807	7,16073799	5,2834E-07	8,1732E-06	6,02521749
TC1700001209.mm.2	319625	Galm	galactose mutarotase	protein-coding	1,32143814	9,03020222	8,77984049	1,96E-08	5,1626E-07	9,36488644
TC0900002770.mm.2	212943	Tent5a	terminal nucleotidyltransferase 5A	protein-coding	1,32114987	6,39576731	7,32033954	3,6595E-07	5,986E-06	6,39371866
TC1400000745.mm.2	17387	Mmp14	matrix metallopeptidase 14 (membrane-inserted)	protein-coding	1,31713095	9,5871003	14,6546017	7,1398E-13	1,5801E-10	19,6175191
TC1000000095.mm.2	66848	Fuca2	fucosidase, alpha-L- 2, plasma	protein-coding	1,31016491	8,33724984	13,148635	6,2024E-12	8,2747E-10	17,4545322
TC0200000969.mm.2	26877	B3galt1	UDP-Gal:betaGlcNAc beta 1,3-galactosyltransferase, polypeptide 1	protein-coding	1,31011685	7,08405953	11,2747941	1,2174E-10	8,4814E-09	14,4567026
TC0600002116.mm.2	338523	Kdm7a	lysine (K)-specific demethylase 7A	protein-coding	1,30470355	5,55391636	5,90630321	9,1488E-06	9,1736E-05	3,15487232
TC0800000286.mm.2	18791	Plat	plasminogen activator, tissue	protein-coding	1,30380184	8,33939003	11,1848291	1,417E-10	9,4524E-09	14,3032932
TC0600001867.mm.2	330267	Thsd7a	thrombospondin, type I, domain containing 7A	protein-coding	1,30199731	8,45269479	14,2059691	1,3333E-12	2,5592E-10	18,9941104
TC1000001216.mm.2	17311	Kitl	kit ligand	protein-coding	1,29807374	9,82019751	12,5503436	1,5463E-11	1,7845E-09	16,5366855
TC1200001544.mm.2	15902	Id2	inhibitor of DNA binding 2	protein-coding	1,29311491	8,71520257	7,63841236	2,1832E-07	3,849E-06	6,93598217
TC1000003148.mm.2	23943	Esyt1	extended synaptotagmin-like protein 1	protein-coding	1,29296513	8,43106294	11,7802169	5,2696E-11	4,7203E-09	15,3017579
TC0200003278.mm.2	64292	Ptges	prostaglandin E synthase	protein-coding	1,29245014	7,69456493	8,486821	2,5032E-08	6,2533E-07	9,08092539
TC1700000618.mm.2	14998	H2-DMa	histocompatibility 2, class II, locus DMa	protein-coding	1,29068819	5,76478854	11,6639718	6,3737E-11	5,4007E-09	15,1098895
TC1300002143.mm.2	13024	Ctla2a	cytotoxic T lymphocyte-associated protein 2 alpha	protein-coding	1,28571699	5,44718956	9,6051254	2,3673E-09	9,1817E-08	11,4533021
TC1000002175.mm.2	17155	Man1a	mannosidase 1, alpha	protein-coding	1,28063031	8,29723763	10,7355632	3,0655E-10	1,7369E-08	13,523273
TC0500000173.mm.2	74511	Lrrc17	leucine rich repeat containing 17	protein-coding	1,27902212	6,0952369	7,27170534	3,3778E-07	5,6078E-06	6,45117379
TC1100003603.mm.2	14422	B4galnt2	beta-1,4-N-acetyl-galactosaminyl transferase 2	protein-coding	1,27851022	5,50556113	8,32692222	2,8888E-08	7,03E-07	8,91567078
TC0900003204.mm.2	207181	Rbms3	RNA binding motif, single stranded interacting protein	protein-coding	1,27850102	8,81887199	11,444974	9,1555E-11	7,001E-09	14,7444005
TC0800000736.mm.2	330790	Hapln4	hyaluronan and proteoglycan link protein 4	protein-coding	1,27534994	5,55946555	12,7258762	1,1787E-11	1,4213E-09	16,8096301
TC0900002728.mm.2	235504	Slc17a5	solute carrier family 17 (anion/sugar transporter), member 5	protein-coding	1,27396846	8,00256624	9,41337965	3,4334E-09	1,2625E-07	11,0773268
TC1400000172.mm.2	73068	Fut11	fucosyltransferase 11	protein-coding	1,27328126	6,68499279	9,27229922	4,4506E-09	1,5305E-07	10,8132317
TC1000000512.mm.2	70297	Gcc2	GRIP and coiled-coil domain containing 2	protein-coding	1,27181002	8,21374854	10,4280335	5,2671E-10	2,6563E-08	12,975646
TC1200002081.mm.2	67963	Npc2	NPC intracellular cholesterol transporter 2	protein-coding	1,2698962	8,85482044	13,2352108	5,4491E-12	7,4704E-10	17,5844718
TC1700000419.mm.2	77128	Crebrf	CREB3 regulatory factor	protein-coding	1,26949041	5,55808138	9,42475827	3,3272E-09	1,2369E-07	11,1082088
TC1700001585.mm.2	22700	Zfp40	zinc finger protein 40	protein-coding	1,26896602	4,81411903	8,89472887	9,2641E-09	2,8101E-07	10,0696144
TC1800000575.mm.2	70417	Megf10	multiple EGF-like-domains 10	protein-coding	1,26886052	5,73883635	11,6073708	6,9958E-11	5,7216E-09	15,0159332
TC1700000836.mm.2	56050	Cyp39a1	cytochrome P450, family 39, subfamily a, polypeptide 1	protein-coding	1,26644712	6,65543673	7,99744673	6,6483E-08	1,385E-06	8,08785894
TC0X00001091.mm.2	213449	Tent5d	terminal nucleotidyltransferase 5D	protein-coding	1,26492993	3,72078923	7,77852808	9,0231E-08	1,8134E-06	7,76004651
TC1800000617.mm.2	16149	Cd74	CD74 antigen (invariant polypeptide of major histocompatibility complex, class II antigen-associated)	protein-coding	1,26379265	6,03222442	7,63511824	1,3835E-07	2,6262E-06	7,34145919
TC0100003301.mm.2	15064	Mr1	major histocompatibility complex, class I-related	protein-coding	1,26335995	7,13950434	7,89751268	8,4992E-08	1,7191E-06	7,84324883
TC0300001236.mm.2	14066	F3	coagulation factor III	protein-coding	1,25939293	8,8503811	12,6069906	1,4161E-11	1,6546E-09	16,6251063
TC0400000580.mm.2	100434	Slc44a1	solute carrier family 44, member 1	protein-coding	1,25836259	8,11910516	8,16870559	5,4806E-08	1,1796E-06	8,3020031
TC1700001345.mm.2	100044193	Gm20939	predicted gene, 20939	protein-coding	1,25745583	7,00750515	8,04131411	6,1384E-08	1,2985E-06	8,17005818
TC0100003483.mm.2	66447	Mgst3	microsomal glutathione S-transferase 3	protein-coding	1,25655221	7,21313646	9,45019194	3,1705E-09	1,1881E-07	11,1571244
TC0500001110.mm.2	72392	Tmem175	transmembrane protein 175	protein-coding	1,25464167	6,90030871	10,6170587	3,7717E-10	2,0384E-08	13,3135864
TC1700001451.mm.2	16004	Igf2r	insulin-like growth factor 2 receptor	protein-coding	1,25455532	9,29197979	13,7570579	2,5314E-12	4,2516E-10	18,3528521
TC1500000237.mm.2	17181	Matn2	matrilin 2	protein-coding	1,24928205	8,65982247	10,5292034	4,4027E-10	2,2944E-08	13,1570518
TC0800002992.mm.2	17132	Maf	MAF bZIP transcription factor	protein-coding	1,24555093	7,37958196	9,95518615	1,2368E-09	5,3117E-08	12,1112261
TC1400001676.mm.2	24056	Sh3bp5	SH3-domain binding protein 5 (BTK-associated)	protein-coding	1,24546946	7,53467983	8,97582186	7,9745E-09	2,5082E-07	10,2226235
TC0300000710.mm.2	12904	Crabp2	cellular retinoic acid binding protein II	protein-coding	1,24291069	9,32192842	12,2100967	2,6389E-11	2,6265E-09	15,9987188
TC0100002285.mm.2	53945	Slc40a1	solute carrier family 40 (iron-regulated transporter), member 1	protein-coding	1,24257105	6,84001729	6,40007632	3,0654E-06	3,6221E-05	4,26058137
TC0300000137.mm.2	12870	Cp	ceruloplasmin	protein-coding	1,23669129	6,92416285	4,56669308	0,00019936	0,00127035	0,05940439
TC0100001526.mm.2	433375	Creg1	cellular repressor of E1A-stimulated genes 1	protein-coding	1,23662164	8,13587595	8,18426662	4,7092E-08	1,0521E-06	8,4421497
TC1700002306.mm.2	240120	Zfp119b	zinc finger protein 119b	protein-coding	1,23598688	4,90460613	8,54462097	1,8586E-08	4,9381E-07	9,36319944
TC0X00001371.mm.2	66889	Rnf128	ring finger protein 128	protein-coding	1,23540812	5,78484955	5,80904177	1,1139E-05	0,00010851	2,95248812
TC1100000641.mm.2	14667	Gm2a	GM2 ganglioside activator protein	protein-coding	1,23397706	7,81162385	7,78785264	1,2339E-07	2,3806E-06	7,48250804
TC0200002537.mm.2	19025	Ctsa	cathepsin A	protein-coding	1,2338364	10,1772057	14,9021747	5,0923E-13	1,2435E-10	19,9543284
TC0200001935.mm.2	17289	Mertk	MER proto-oncogene tyrosine kinase	protein-coding	1,23379893	4,77629088	8,55669881	1,814E-08	4,8401E-07	9,38784183
TC1700002324.mm.2	19280	Ptprs	protein tyrosine phosphatase receptor type S	protein-coding	1,23047193	10,1106496	12,9189806	8,7732E-12	1,0929E-09	17,1063646
TC1100000547.mm.2	216725	Adamts2	ADAM metallopeptidase with thrombospondin type 1 motif 2	protein-coding	1,22765382	6,69317489	9,90928056	1,3456E-09	5,6625E-08	12,0258299
TC1000001147.mm.2	22025	Nr2c1	nuclear receptor subfamily 2, group C, member 1	protein-coding	1,22692957	7,33266322	11,508734	8,235E-11	6,4815E-09	14,8513571
TC1700002584.mm.2	106794	Dhx57	DExH-box helicase 57	protein-coding	1,22415013	7,66101648	13,3064223	4,9009E-12	6,9487E-10	17,6908189
TC0400002668.mm.2	381524	Mexis	macrophage expressed LXRa(NR1H3)-dependent amplifier of Abca1 transcription lncRNA	ncRNA	1,22223414	5,040705	6,57896806	1,4045E-06	1,8661E-05	4,99431564

TC1700000391.mm.2	72106	Jmjd8	jumonji domain containing 8	protein-coding	1,21538252	7,64795114	10,7593765	2,941E-10	1,6816E-08	13,5652086
TC0600003005.mm.2	12288	Cacna1c	calcium channel, voltage-dependent, L type, alpha 1C subunit	protein-coding	1,21155996	6,93784902	12,892391	9,1354E-12	1,1306E-09	17,0657231
TC1600000378.mm.2	239790	Ostn	osteocrin	protein-coding	1,21060201	5,87516512	8,35205013	2,7446E-08	6,7488E-07	8,96765213
TC1500000747.mm.2	24050	Septin3	septin 3	protein-coding	1,20985808	4,64004426	7,92698733	6,6028E-08	1,3801E-06	8,07688885
TC1300001135.mm.2	20365	Serf1	small EDRK-rich factor 1	protein-coding	1,20900146	6,13034671	9,62399164	2,285E-09	8,9549E-08	11,4891572
TC0300000765.mm.2	229534	Pbxip1	pre B cell leukemia transcription factor interacting protein 1	protein-coding	1,20897645	5,79101735	9,83009009	1,5571E-09	6,4516E-08	11,877893
TC0500001382.mm.2	74096	Hvcn1	hydrogen voltage-gated channel 1	protein-coding	1,20444986	5,88287615	8,59628281	1,6754E-08	4,561E-07	9,46846788
TC0900001800.mm.2	72560	Naalad2	N-acetylated alpha-linked acidic dipeptidase 2	protein-coding	1,20417942	5,75396166	8,83064812	1,0511E-08	3,1186E-07	9,94153977
TC0100001481.mm.2	17926	Myoc	myocilin	protein-coding	1,20410288	6,15959123	5,90327632	8,9753E-06	9,0093E-05	3,16995445
TC0200001866.mm.2	214968	Sema6d	sema domain, transmembrane domain (TM), and cytoplasmic domain, (semaphorin) 6D	protein-coding	1,20094376	4,82482993	7,44969841	1,8211E-07	3,3162E-06	7,04775727
TC1400002071.mm.2	59049	Slc22a17	solute carrier family 22 (organic cation transporter), member 17	protein-coding	1,19512128	6,17509791	9,70100192	1,9787E-09	7,9195E-08	11,6350432
TC1100000053.mm.2	18082	Nipsnap1	nipsnap homolog 1	protein-coding	1,19160644	6,63935261	8,84668388	1,0183E-08	3,031E-07	9,97364073
TC0100002656.mm.2	210293	Dock10	dedicator of cytokinesis 10	protein-coding	1,1912902	4,46748188	8,68441265	1,4048E-08	3,9324E-07	9,64722043
TC1400001331.mm.2	69634	Clybl	citrate lyase beta like	protein-coding	1,18992219	6,88822137	10,3942255	5,5937E-10	2,7837E-08	12,9147502
TC1700002588.mm.2	225028	Map4k3	mitogen-activated protein kinase kinase kinase kinase 3	protein-coding	1,18878342	8,01182357	7,58425686	1,8735E-07	3,4018E-06	7,0581324
TC1200001813.mm.2	20334	Sec23a	SEC23 homolog A, COPII coat complex component	protein-coding	1,18737813	8,58529087	6,59493353	2,1182E-06	2,6406E-05	4,64277715
TC1000002497.mm.2	66548	Adamtsl5	ADAMTS-like 5	protein-coding	1,18588018	8,13494432	13,255481	5,2868E-12	7,3667E-10	17,6147922
TC1100004270.mm.2	380712	Tlcd2	TLC domain containing 2	protein-coding	1,18480749	8,61710872	8,17831299	4,6456E-08	1,0391E-06	8,45300767
TC1000003105.mm.2	117150	Pip4k2c	phosphatidylinositol-5-phosphate 4-kinase, type II, gamma	protein-coding	1,1847546	9,41601696	12,2829998	2,3511E-11	2,4301E-09	16,1149881
TC0100000129.mm.2	74229	Paqr8	progestin and adipoQ receptor family member VIII	protein-coding	1,18155411	5,53245802	11,1985238	1,3846E-10	9,2687E-09	14,326705
TC0600002188.mm.2	29807	Tpk1	thiamine pyrophosphokinase	protein-coding	1,18134464	5,52504591	7,15765738	3,5723E-07	5,8586E-06	6,36958213
TC0300000043.mm.2	20257	Stmn2	stathmin-like 2	protein-coding	1,17816712	7,23216879	4,59518272	0,00018675	0,00120304	0,12487553
TC1200001601.mm.2	73389	Hbp1	high mobility group box transcription factor 1	protein-coding	1,17648146	8,65133105	12,3855088	2,0005E-11	2,2267E-09	16,2775455
TC1200002122.mm.2	20773	Sptlc2	serine palmitoyltransferase, long chain base subunit 2	protein-coding	1,17544391	9,02785028	6,11025903	6,4034E-06	6,7143E-05	3,5309408
TC0500002244.mm.2	16976	Lrpap1	low density lipoprotein receptor-related protein associated protein 1	protein-coding	1,17496451	8,54116118	8,2472554	4,0416E-08	9,2378E-07	8,59444017
TC1400000742.mm.2	105501	Abhd4	abhydrolase domain containing 4	protein-coding	1,17486404	9,52176002	8,8012079	1,3503E-08	3,8031E-07	9,70768369
TC1200000869.mm.2	14874	Gstz1	glutathione transferase zeta 1 (maleylacetoacetate isomerase)	protein-coding	1,17433797	7,55684209	7,32440413	3,2098E-07	5,3527E-06	6,51085022
TC1000001708.mm.2	338362	Ust	uronyl-2-sulfotransferase	protein-coding	1,17196213	7,06770677	10,9452137	2,1326E-10	1,2941E-08	13,8901847
TC1700001563.mm.2	73233	Zfp942	zinc finger protein 942	protein-coding	1,1715796	7,25867119	5,08181188	6,1696E-05	0,00046797	1,23823846
TC1200002203.mm.2	71375	Foxn3	forkhead box N3	protein-coding	1,17121559	7,41675194	9,94350148	1,2636E-09	5,3959E-08	12,0895146
TSUnmapped00000040.mm.2	225998	Rorb	RAR-related orphan receptor beta	protein-coding	1,17040595	7,98768766	8,89788943	9,2068E-09	2,7999E-07	10,0759171
TC0200003806.mm.2	14231	Fkbp7	FK506 binding protein 7	protein-coding	1,16794865	9,40611277	7,20986259	5,4641E-07	8,3974E-06	6,00936796
TC0600001313.mm.2	20315	Cxcl12	C-X-C motif chemokine ligand 12	protein-coding	1,16775332	11,9866778	14,0805167	1,5922E-12	2,8799E-10	18,8166947
TC1700000351.mm.2	27361	Msrb1	methionine sulfoxide reductase B1	protein-coding	1,16749632	8,17151064	8,5964957	1,7224E-08	4,6418E-07	9,44344074
TC0700004580.mm.2	63830	Kcnq1ot1	KCNQ1 overlapping transcript 1	ncRNA	1,16614529	3,98649209	5,66423284	1,2108E-05	0,00011669	2,82668125
TC1000001946.mm.2	66847	Hint3	histidine triad nucleotide binding protein 3	protein-coding	1,16512235	7,88014563	10,0641007	1,0136E-09	4,5508E-08	12,3127814
TC1000002000.mm.2	319638	Nt5dc1	5'-nucleotidase domain containing 1	protein-coding	1,16475393	7,48193704	9,23397098	4,7905E-09	1,6325E-07	10,7385985
TC0400000093.mm.2	12557	Cdh17	cadherin 17	protein-coding	1,16238395	5,69755531	7,36372292	2,1933E-07	3,8523E-06	6,85915563
TC0700001169.mm.2	18584	Pde8a	phosphodiesterase 8A	protein-coding	1,16221327	7,71697903	11,4290553	9,4015E-11	7,1025E-09	14,7176265
TC0800000530.mm.2	66756	Cfap97	cilia and flagella associated protein 97	protein-coding	1,16124296	7,63265603	7,94497342	7,1984E-08	1,4783E-06	8,00379735
TC1900000141.mm.2	225845	Plaat3	phospholipase A and acyltransferase 3	protein-coding	1,16083605	6,60004166	9,31089058	4,1335E-09	1,4616E-07	10,8881836
TC1000000753.mm.2	80294	Pofut2	protein O-fucosyltransferase 2	protein-coding	1,15528371	9,11234438	12,6931057	1,2397E-11	1,476E-09	16,7589074
TC1200002251.mm.2	19141	Lgmn	legumain	protein-coding	1,15481699	10,4635259	13,2487624	5,34E-12	7,3861E-10	17,6047468
TC0900000090.mm.2	270118	Maml2	mastermind like transcriptional coactivator 2	protein-coding	1,15299701	6,33974828	7,52536672	1,5885E-07	2,9393E-06	7,18959154
TC1400000195.mm.2	54169	Kat6b	K(lysine) acetyltransferase 6B	protein-coding	1,15274947	7,62430403	9,99175911	1,1567E-09	5,0161E-08	12,1790724
TC1100001988.mm.2	56745	C1qtnf1	C1q and tumor necrosis factor related protein 1	protein-coding	1,15273109	7,43123364	8,09091249	4,9962E-08	1,0979E-06	8,36697752
TC0600001193.mm.2	16438	Itpr1	inositol 1,4,5-trisphosphate receptor 1	protein-coding	1,15201359	7,86654525	10,0139783	1,1106E-09	4,8928E-08	12,2202094
TC0700004431.mm.2	11489	Adam12	ADAM metallopeptidase domain 12	protein-coding	1,15049745	7,68970089	9,93737988	1,2779E-09	5,4262E-08	12,0781332
TC1200001885.mm.2	110095	Pygl	liver glycogen phosphorylase	protein-coding	1,14822477	8,42219747	10,5100606	4,5542E-10	2,3665E-08	13,1228217
TC0500001641.mm.2	74570	Zkscan1	zinc finger with KRAB and SCAN domains 1	protein-coding	1,14720579	6,48919091	7,84451047	7,8509E-08	1,5983E-06	7,90123191
TC0X00001710.mm.2	17318	Mid1	midline 1	protein-coding	1,14444535	8,51586088	10,6943927	3,2939E-10	1,8386E-08	13,4506132
TC1000000590.mm.2	73132	Slc25a16	solute carrier family 25 (mitochondrial carrier, Graves disease autoantigen), member 16	protein-coding	1,14434759	8,20028688	11,6296472	6,7437E-11	5,6131E-09	15,0529534
TC1000002353.mm.2	83675	Bicc1	BicC family RNA binding protein 1	protein-coding	1,14337128	8,85949656	9,7334081	1,8628E-09	7,5521E-08	11,6962066
TC1000000191.mm.2	76306	Slc18b1	solute carrier family 18, subfamily B, member 1	protein-coding	1,14176116	7,23834636	6,99064534	6,519E-07	9,694E-06	5,79142591
TC0900000686.mm.2	121021	Cspg4	chondroitin sulfate proteoglycan 4	protein-coding	1,14021965	5,73156865	8,56612917	1,7799E-08	4,7764E-07	9,40706895
TC0600001595.mm.2	12576	Cdkn1b	cyclin dependent kinase inhibitor 1B	protein-coding	1,13842314	8,89899475	11,5463973	7,7369E-11	6,217E-09	14,9143253
TC0800000955.mm.2	12286	Cacna1a	calcium channel, voltage-dependent, P/Q type, alpha 1A subunit	protein-coding	1,13808896	5,24136393	7,71802792	1,0256E-07	2,0309E-06	7,63007856
TC0100002576.mm.2	21961	Tns1	tensin 1	protein-coding	1,13773781	7,9586777	8,0932259	5,0537E-08	1,1049E-06	8,35723995
TC0200005113.mm.2	17183	Matn4	matrilin 4	protein-coding	1,13712235	5,22381778	6,78624574	7,8891E-07	1,1457E-05	5,56268504
TC1000002050.mm.2	103199	Fig4	FIG4 phosphoinositide 5-phosphatase	protein-coding	1,13696766	8,68645445	10,3648752	5,8942E-10	2,8949E-08	12,8617712
TC1200000679.mm.2	15512	Hspa2	heat shock protein 2	protein-coding	1,13628438	6,2174492	10,0906114	9,6593E-10	4,3783E-08	12,3616184
TC0700003990.mm.2	12751	Tpp1	tripeptidyl peptidase I	protein-coding	1,13525555	10,0807097	11,2909466	1,1847E-10	8,2847E-09	14,4841492
TC1200000319.mm.2	217473	Ankmy2	ankyrin repeat and MYND domain containing 2	protein-coding	1,13509294	7,26015375	8,18580952	3,8578E-08	8,8932E-07	8,62217579
TC0200001847.mm.2	99439	Duox1	dual oxidase 1	protein-coding	1,13453628	5,87676433	8,35390412	2,7342E-08	6,7322E-07	8,97148404
TC1700001289.mm.2	18754	Prkce	protein kinase C, epsilon	protein-coding	1,13305257	6,19668446	7,95447213	6,2339E-08	1,3112E-06	8,13522197
TC0500003513.mm.2	78339	Ttyh3	tweety family member 3	protein-coding	1,13241741	8,42993046	11,3294078	1,1106E-10	7,8541E-09	14,5493843
TC0100000870.mm.2	12778	Ackr3	atypical chemokine receptor 3	protein-coding	1,13225515	6,93619509	7,90940176	6,8506E-08	1,4177E-06	8,03951229
TC0600002466.mm.2	68477	Rmnd5a	required for meiotic nuclear division 5 homolog A	protein-coding	1,13208349	7,75835819	8,0027964	5,9929E-08	1,2738E-06	8,18235333
TC1200000664.mm.2	80837	Rhoj	ras homolog family member J	protein-coding	1,13121808	8,59206444	10,06227	1,017E-09	4,5551E-08	12,3094057
TC1300000565.mm.2	407800	Ecm2	extracellular matrix protein 2, female organ and adipocyte specific	protein-coding	1,12755412	3,81506423	7,15912299	3,4279E-07	5,6713E-06	6,40643553
TC1400000177.mm.2	22330	Vcl	vinculin	protein-coding	1,12745618	8,61338081	11,4474976	9,1171E-11	7,0001E-09	14,7486424
TC1000000547.mm.2	19156	Psap	prosaposin	protein-coding	1,12728445	11,5182615	15,6742106	1,8275E-13	5,131E-11	20,9731404
TC0700002497.mm.2	11816	Apoe	apolipoprotein E	protein-coding	1,12684308	6,36541576	8,20971348	3,6726E-08	8,5715E-07	8,67208143
TC1700001147.mm.2	77889	Lbh	limb-bud and heart	protein-coding	1,12552029	7,73236677	12,0019344	3,6801E-11	3,4963E-09	15,6636746
TC1400002632.mm.2	239217	Kctd12	potassium channel tetramerisation domain containing 12	protein-coding	1,12313218	7,54745695	8,74425434	1,2471E-08	3,5762E-07	9,76800482
TC0X00003114.mm.2	12338	Capn6	calpain 6	protein-coding	1,1227216	6,76662878	6,05583996	5,773E-06	6,1896E-05	3,60001552
TC0300000722.mm.2	56700	Glmp	glycosylated lysosomal membrane protein	protein-coding	1,12100597	9,45150919	12,7044789	1,2182E-11	1,4596E-09	16,7765231
TC1000000653.mm.2	327766	Tmem26	transmembrane protein 26	protein-coding	1,12075044	8,06702612	7,96150282	6,7244E-08	1,3977E-06	8,06894759
TC0500000131.mm.2	20348	Sema3c	sema domain, immunoglobulin domain (Ig), short basic domain, secreted, (semaphorin) 3C	protein-coding	1,12058844	8,202095	10,3947445	5,5885E-10	2,7837E-08	12,915686
TC0700001086.mm.2	75547	Akap13	A kinase anchor protein 13	protein-coding	1,11967467	5,03328452	5,6183238	1,4283E-05	0,00013441	2,67007942
TC1700001303.mm.2	104215	Rhoq	ras homolog family member Q	protein-coding	1,11889515	9,66007838	11,900587	4,3338E-11	3,9962E-09	15,4988949
TC0400001286.mm.2	68777	Tmem53	transmembrane protein 53	protein-coding	1,11869086	3,65251236	6,79248897	7,7362E-07	1,1272E-05	5,58173579
TC0200004227.mm.2	12359	Cat	catalase	protein-coding	1,118526	9,40059168	8,44383932	2,6968E-08	6,6486E-07	9,00407157
TC0600001530.mm.2	93694	Clec2d	C-type lectin domain family 2, member d	protein-coding	1,11832807	8,66607572	5,89449056	1,0197E-05	0,00010042	3,05885253
TC0500002480.mm.2	21897	Tlr1	toll-like receptor 1	protein-coding	1,11746608	6,18980307	8,36506481	2,6728E-08	6,633E-07	8,99454181
TC1000002380.mm.2	71733	Susd2	sushi domain containing 2	protein-coding	1,11621092	5,8571235	5,32575878	3,2694E-05	0,00027201	1,86279176
TC0400001631.mm.2	433766	Trim63	tripartite motif-containing 63	protein-coding	1,11575787	6,4889992	9,23046061	4,8229E-09	1,6406E-07	10,7317535
TC1700001693.mm.2	106581	Fam234a	family with sequence similarity 234, member A	protein-coding	1,11566767	7,75194762	9,39097552	3,5479E-09	1,2859E-07	11,0431062
TC1400000986.mm.2	21933	Tnfrsf10b	tumor necrosis factor receptor superfamily, member 10b	protein-coding	1,11518004	7,45011723	10,0995644	9,5035E-10	4,3286E-08	12,3780914
TC1900001266.mm.2	71738	Mamdc2	MAM domain containing 2	protein-coding	1,11492472	5,78416312	8,80269706	1,1108E-08	3,2385E-07	9,8855046
TC0700002988.mm.2	52377	Rcn3	reticulocalbin 3, EF-hand calcium binding domain	protein-coding	1,11441857	9,0349708	8,27347921	3,7113E-08	8,6403E-07	8,67733764
TC0900002767.mm.2	83603	Elovl4	ELOVL fatty acid elongase 4	protein-coding	1,11339095	6,42458132	7,34981544	2,3094E-07	4,0298E-06	6,80957999
TC0200005462.mm.2	12496	Entpd2	ectonucleoside triphosphate diphosphohydrolase 2	protein-coding	1,11227878	6,2640232	6,25257601	3,3658E-06	3,9107E-05	4,13055804
TC1000000517.mm.2	19386	Ranbp2	RAN binding protein 2	protein-coding	1,11177497	9,46202578	6,52798759	2,4393E-06	2,9835E-05	4,49933512
TC1000001198.mm.2	67972	Atp2b1	ATPase, Ca++ transporting, plasma membrane 1	protein-coding	1,11071064	7,60528299	9,86760775	1,4529E-09	6,0465E-08	11,9480787
TC0600001992.mm.2	22214	Ube2h	ubiquitin-conjugating enzyme E2H	protein-coding	1,10835114	8,95988241	10,0841791	9,7729E-10	4,4086E-08	12,349777
TC0200002399.mm.2	98932	Myl9	myosin, light polypeptide 9, regulatory	protein-coding	1,10775829	9,26951108	7,21752993	4,5966E-07	7,2707E-06	6,16391683
TC0700001430.mm.2	56018	Stard10	StAR related lipid transfer domain containing 10	protein-coding	1,10618479	6,22891967	8,60690244	1,6401E-08	4,4909E-07	9,49006261
TC0800000585.mm.2	11593	Aga	aspartylglucosaminidase	protein-coding	1,1053805	8,6640138	11,6829879	6,1778E-11	5,3064E-09	15,141377
TC0800000714.mm.2	211135	Zfp781a	zinc finger protein 781A	protein-coding	1,10417714	5,27308085	5,49513119	1,9881E-05	0,00017825	2,34464161
TC0300002543.mm.2	83679	Pde4dip	phosphodiesterase 4D interacting protein (myomegalin)	protein-coding	1,10377317	7,53042517	10,7288189	3,1018E-10	1,7469E-08	13,5113842
TC0300003013.mm.2	26377	Dapp1	dual adaptor for phosphotyrosine and 3-phosphoinositides 1	protein-coding	1,10334454	6,25948157	5,3385276	3,2244E-05	0,00026886	1,87957786
TC0600003148.mm.2	12527	Cd9	CD9 antigen	protein-coding	1,10326853	10,5313595	11,0617605	1,7467E-10	1,1101E-08	14,0919417
TSUnmapped00000014.mm.2	67528	Nudt7	nudix hydrolase 7	protein-coding	1,10269663	4,7255773	5,47790159	1,9369E-05	0,00017424	2,35933178
TC0600000319.mm.2	74354	Lrguk	leucine-rich repeats and guanylate kinase domain containing	protein-coding	1,10142043	3,62392823	8,09693168	4,6349E-08	1,0389E-06	8,43594518
TC1400001914.mm.2	12159	Bmp4	bone morphogenetic protein 4	protein-coding	1,09979127	7,25983844	5,61437133	1,7714E-05	0,00016176	2,48845548
TC1400001253.mm.2	23888	Gpc6	glypican 6	protein-coding	1,09951684	7,58407773	10,4977406	4,6545E-10	2,4054E-08	13,1007685
TC0700001879.mm.2	21804	Tgfb1i1	transforming growth factor beta 1 induced transcript 1	protein-coding	1,09747633	7,39270358	11,3442184	1,0834E-10	7,7195E-09	14,5744604
TC0300000968.mm.2	108097	Prkab2	protein kinase, AMP-activated, beta 2 non-catalytic subunit	protein-coding	1,09712788	5,58881893	8,42421332	2,3702E-08	5,9767E-07	9,11646166
TC1000001993.mm.2	103511	Calhm5	calcium homeostasis modulator family member 5	protein-coding	1,09359156	7,20795684	9,40766443	3,437E-09	1,2625E-07	11,0752858
TC1500001570.mm.2	67731	Fbxo32	F-box protein 32	protein-coding	1,09258102	5,81443056	7,60992646	1,291E-07	2,4706E-06	7,39663318
TC0800001077.mm.2	17390	Mmp2	matrix metallopeptidase 2	protein-coding	1,09165368	10,0468411	7,67758074	1,7898E-07	3,2688E-06	7,12284114

TC1000001044.mm.2	21859	Timp3	tissue inhibitor of metalloproteinase 3	protein-coding	1,09002882	8,85664525	8,12171674	4,8376E-08	1,0718E-06	8,40329528
TC0600001917.mm.2	269831	Tspan12	tetraspanin 12	protein-coding	1,0897904	6,63862299	7,36531176	2,2E-07	3,8568E-06	6,8568951
TC1700002361.mm.2	67993	Nudt12	nudix hydrolase 12	protein-coding	1,08543861	6,37438426	6,70037236	1,0638E-06	1,4788E-05	5,27473387
TC0600002965.mm.2	67784	Plxnd1	plexin D1	protein-coding	1,08520901	6,66950022	8,87929732	9,5496E-09	2,8788E-07	10,0388221
TC0100000813.mm.2	98363	Efhd1	EF hand domain containing 1	protein-coding	1,08512597	4,70800162	8,46131683	2,1986E-08	5,627E-07	9,19270122
TC1200001976.mm.2	110417	Pigh	phosphatidylinositol glycan anchor biosynthesis, class H	protein-coding	1,08394783	5,86036966	8,42838139	2,3503E-08	5,9503E-07	9,12503537
TC1100002314.mm.2	216551	Lgalsl	galectin like	protein-coding	1,08389373	9,4954401	9,78622508	1,6888E-09	6,982E-08	11,7956078
TC0600001154.mm.2	232313	Gxylt2	glucoside xylosyltransferase 2	protein-coding	1,08160908	8,30553647	8,32462131	2,9024E-08	7,0358E-07	8,91090671
TC1000001010.mm.2	319277	Washc4	WASH complex subunit 4	protein-coding	1,08042255	8,96962374	9,35587777	3,7932E-09	1,3617E-07	10,9753129
TC0700004665.mm.2	27973	Vkorc1	vitamin K epoxide reductase complex, subunit 1	protein-coding	1,07950972	9,17077134	8,82349051	1,066E-08	3,1382E-07	9,92720037
TC0400002132.mm.2	56711	Plag1	pleiomorphic adenoma gene 1	protein-coding	1,07949625	5,98735951	6,07753473	4,6791E-06	5,1654E-05	3,7870228
TC0400000535.mm.2	230157	Tmeff1	transmembrane protein with EGF-like and two follistatin-like domains 1	protein-coding	1,07915752	7,86525836	9,75666524	1,784E-09	7,3114E-08	11,74002
TC0600002663.mm.2	69834	Rab43	RAB43, member RAS oncogene family	protein-coding	1,0774057	6,49180563	8,4967914	2,0466E-08	5,3322E-07	9,26542075
TC1200002224.mm.2	104718	Ttc7b	tetratricopeptide repeat domain 7B	protein-coding	1,07541281	6,34143361	9,03998539	6,9723E-09	2,2268E-07	10,357916
TC1000001901.mm.2	432442	Akap7	A kinase anchor protein 7	protein-coding	1,07390782	7,13384328	9,20823473	5,0337E-09	1,6969E-07	10,6883769
TC1000001807.mm.2	56016	Hebp2	heme binding protein 2	protein-coding	1,07247966	6,83384204	9,76907631	1,7434E-09	7,1761E-08	11,7633727
TC1700001740.mm.2	106672	Smim29	small integral membrane protein 29	protein-coding	1,07139704	8,24927763	10,1472338	8,7165E-10	4,0386E-08	12,4656339
TC0500001821.mm.2	77521	Mtus2	microtubule associated tumor suppressor candidate 2	protein-coding	1,07135729	7,546044	7,54215386	1,5775E-07	2,9236E-06	7,20020167
TC0800001078.mm.2	270084	Lpcat2	lysophosphatidylcholine acyltransferase 2	protein-coding	1,07005514	5,41978417	9,29049936	4,298E-09	1,5E-07	10,8486041
TC0600000663.mm.2	330323	Mindy4	MINDY lysine 48 deubiquitinase 4	protein-coding	1,06896235	6,36370246	8,5173211	1,9636E-08	5,1626E-07	9,30742751
TC1400002217.mm.2	14463	Gata4	GATA binding protein 4	protein-coding	1,06807899	7,06148471	8,70962914	1,336E-08	3,7735E-07	9,69817564
TC1300002717.mm.2	109700	Itga1	integrin alpha 1	protein-coding	1,06800647	5,18664495	8,65260355	1,4969E-08	4,1653E-07	9,58282229
TC0600000850.mm.2	56316	Ggcx	gamma-glutamyl carboxylase	protein-coding	1,06757837	9,52980373	10,3020127	6,5957E-10	3,1977E-08	12,7479481
TC0600000190.mm.2	93735	Wnt16	wingless-type MMTV integration site family, member 16	protein-coding	1,06669026	7,10440889	9,35969213	3,7657E-09	1,3544E-07	10,9826883
TC1100001661.mm.2	217203	Tmem106a	transmembrane protein 106A	protein-coding	1,063746	6,59487378	8,0081227	5,5737E-08	1,1942E-06	8,24879582
TC0300000017.mm.2	80892	Zfhx4	zinc finger homeodomain 4	protein-coding	1,06246082	8,29011391	6,16914056	4,6716E-06	5,1602E-05	3,82068446
TC1700000198.mm.2	22759	Zfp97	zinc finger protein 97	protein-coding	1,0619249	8,34593529	7,40158996	2,3312E-07	4,0566E-06	6,8153727
TC0X00003042.mm.2	100034363	Tmsb15b2	thymosin beta 15b2	protein-coding	1,06048982	3,68955077	6,05000312	4,2358E-06	4,7626E-05	3,86220199
TC1200002049.mm.2	217695	Zfyve1	zinc finger, FYVE domain containing 1	protein-coding	1,06042021	7,07880333	6,80304133	8,5362E-07	1,223E-05	5,49886433
TC1700001561.mm.2	70081	Zfp995	zinc finger protein 995	protein-coding	1,05991368	6,43155215	6,78417442	8,4317E-07	1,2108E-05	5,50384176
TC1500001942.mm.2	17939	Naga	N-acetyl galactosaminidase, alpha	protein-coding	1,0593875	8,69062329	11,213942	1,3489E-10	9,1606E-09	14,3530374
TC1700000292.mm.2	74149	Zfp946	zinc finger protein 946	protein-coding	1,05919495	7,14630103	6,85997946	7,5294E-07	1,1022E-05	5,6260804
TC1000001110.mm.2	67454	Ikbip	IKBKB interacting protein	protein-coding	1,05876359	9,3515115	7,84737367	9,146E-08	1,8303E-06	7,7651771
TC0500001853.mm.2	381694	B3glct	beta-3-glucosyltransferase	protein-coding	1,05628725	7,42596779	8,20149663	3,7352E-08	8,6745E-07	8,65493534
TC0500001636.mm.2	381678	Zcwpw1	zinc finger, CW type with PWWP domain 1	protein-coding	1,05586939	5,80094804	9,04570603	6,8951E-09	2,2096E-07	10,3692131
TC0300001703.mm.2	27357	Gyg1	glycogenin 1	protein-coding	1,05249587	6,97951597	8,51845229	1,9591E-08	5,1626E-07	9,30974044
TC1600001648.mm.2	66067	Gtpbp8	GTP-binding protein 8 (putative)	protein-coding	1,05235262	5,79207432	5,99263859	5,3236E-06	5,7853E-05	3,64626275
TC0100002942.mm.2	14633	Gli2	GLI-Kruppel family member GLI2	protein-coding	1,05131182	5,40187779	6,5905287	1,2203E-06	1,667E-05	5,12031746
TC1000000021.mm.2	83397	Akap12	A kinase anchor protein 12	protein-coding	1,05005628	8,53244	9,32729026	4,0059E-09	1,4245E-07	10,9199763
TC1100002907.mm.2	11671	Aldh3a2	aldehyde dehydrogenase family 3, subfamily A2	protein-coding	1,04942109	7,90774497	5,5153478	2,211E-05	0,00019444	2,26444322
TC1700000069.mm.2	381059	Gm1604b	predicted gene 1604b	protein-coding	1,04932392	9,51578226	8,47950094	2,2557E-08	5,7341E-07	9,17352489
TC1000002236.mm.2	107449	Unc5b	unc-5 netrin receptor B	protein-coding	1,04843264	8,41051187	9,54356896	2,6577E-09	1,0141E-07	11,3359975
TC1000000332.mm.2	19714	Rev3l	REV3 like, DNA directed polymerase zeta catalytic subunit	protein-coding	1,04827417	7,23944587	7,64208809	1,2054E-07	2,34E-06	7,4662494
TC1200001106.mm.2	214305	Hhipl1	hedgehog interacting protein-like 1	protein-coding	1,04696903	6,90014625	8,69003053	1,3892E-08	3,9002E-07	9,65857989
TC1700000564.mm.2	70101	Cyp4f16	cytochrome P450, family 4, subfamily f, polypeptide 16	protein-coding	1,04557821	7,10404719	10,5014134	4,6244E-10	2,3964E-08	13,1073448
TC1700001386.mm.2	100040525	Tmem181c-ps	transmembrane protein 181C, pseudogene	pseudo	1,04531135	8,43315308	11,1587701	1,481E-10	9,8442E-09	14,2586853
TC1000003052.mm.2	117600	Srgap1	SLIT-ROBO Rho GTPase activating protein 1	protein-coding	1,04524147	6,9728951	9,19433642	5,1703E-09	1,7367E-07	10,6612197
TC0400000118.mm.2	12395	Runx1t1	RUNX1 translocation partner 1	protein-coding	1,04515664	6,65416419	7,89254331	7,0971E-08	1,4623E-06	8,00364214
TC0X00001335.mm.2	237052	Tceal1	transcription elongation factor A (SII)-like 1	protein-coding	1,04472991	5,98081086	9,28043246	4,3817E-09	1,5135E-07	10,8290441
TC1200001017.mm.2	27277	Golga5	golgin A5	protein-coding	1,04269487	7,98941033	6,7799107	9,5717E-07	1,3507E-05	5,39165953
TC1900000467.mm.2	23972	Papss2	3'-phosphoadenosine 5'-phosphosulfate synthase 2	protein-coding	1,04243662	7,69622558	6,89273239	7,0063E-07	1,0345E-05	5,69907474
TC1000000045.mm.2	237253	Lrp11	low density lipoprotein receptor-related protein 11	protein-coding	1,04163785	6,8302034	8,87044733	9,7175E-09	2,92E-07	10,0211484
TC0900000223.mm.2	319845	Bbs9	Bardet-Biedl syndrome 9	protein-coding	1,04064794	7,32411434	8,73750429	1,264E-08	3,597E-07	9,75440445
TC0800000010.mm.2	94178	Mcoln1	mucolipin 1	protein-coding	1,04038699	8,81042068	9,28005558	4,3849E-09	1,5135E-07	10,8283116
TC0500003619.mm.2	140887	Lnx2	ligand of numb-protein X 2	protein-coding	1,03934448	8,00435231	8,31125126	2,9827E-08	7,2119E-07	8,8832097
TC0600002644.mm.2	11746	Anxa4	annexin A4	protein-coding	1,03916881	10,756357	10,9525164	2,106E-10	1,2831E-08	13,9028728
TC1100004000.mm.2	217262	Abca9	ATP-binding cassette, sub-family A member 9	protein-coding	1,03787907	4,74099943	6,94875259	5,4568E-07	8,3959E-06	5,93530997
TC1000000915.mm.2	408062	Zfp873	zinc finger protein 873	protein-coding	1,03728856	6,26334752	7,21946318	3,0032E-07	5,0396E-06	6,54051778
TC1700001839.mm.2	54194	Akap8l	A kinase anchor protein 8-like	protein-coding	1,03701853	7,56532321	8,51696264	1,965E-08	5,1626E-07	9,30669453
TC1700002240.mm.2	76441	Daam2	dishevelled associated activator of morphogenesis 2	protein-coding	1,03421911	7,90957767	10,8113833	2,687E-10	1,5697E-08	13,6565613
TC0100003826.mm.2	214742	Rcor3	REST corepressor 3	protein-coding	1,0340798	6,85243496	7,36699515	2,1778E-07	3,843E-06	6,86635163
TC0800000416.mm.2	319520	Dusp4	dual specificity phosphatase 4	protein-coding	1,03222854	7,08269559	6,90996142	6,4681E-07	9,6336E-06	5,77436711
TC0300001342.mm.2	56811	Dkk2	dickkopf WNT signaling pathway inhibitor 2	protein-coding	1,03211427	7,93881657	7,21115485	3,3942E-07	5,6255E-06	6,42993156
TC1000000907.mm.2	100233208	Gm10778	predicted gene 10778	protein-coding	1,03178401	7,20283822	7,25101625	2,8208E-07	4,7846E-06	6,60486682
TC0600000565.mm.2	317758	Gimap9	GTPase, IMAP family member 9	protein-coding	1,03146163	5,63932512	6,7426373	8,6532E-07	1,2378E-05	5,46830055
TC1200000576.mm.2	68055	Dmac2l	distal membrane arm assembly component 2 like	protein-coding	1,03138494	5,55825157	6,92227653	5,7879E-07	8,8017E-06	5,87561812
TC1600001006.mm.2	15980	Ifngr2	interferon gamma receptor 2	protein-coding	1,03126528	7,96991531	11,3866793	1,0091E-10	7,3571E-09	14,6462158
TC1700001158.mm.2	210148	Slc30a6	solute carrier family 30 (zinc transporter), member 6	protein-coding	1,03100038	9,03768539	7,08253319	5,2501E-07	8,1417E-06	6,00849404
TC1700001408.mm.2	100037283	Rnaset2a	ribonuclease T2A	protein-coding	1,03080942	9,53461898	8,30394427	3,2323E-08	7,648E-07	8,80900654
TC0400004146.mm.2	26561	Mmp23	matrix metallopeptidase 23	protein-coding	1,03080328	7,49919759	7,08413516	4,4138E-07	7,0303E-06	6,16180473
TC0400003998.mm.2	100041677	Zfp984	zinc finger protein 984	protein-coding	1,03080299	7,15642753	7,83365552	8,0324E-08	1,6317E-06	7,87804526
TC1100002449.mm.2	66753	Erlec1	endoplasmic reticulum lectin 1	protein-coding	1,0292523	8,45157349	7,42948175	2,0936E-07	3,7188E-06	6,91837224
TC0100001533.mm.2	100039795	Ildr2	immunoglobulin-like domain containing receptor 2	protein-coding	1,02569128	4,45986892	5,54100556	1,4948E-05	0,00013996	2,60105026
TC0200000037.mm.2	16922	Phyh	phytanoyl-CoA hydroxylase	protein-coding	1,02460026	6,70809933	6,38616013	2,1668E-06	2,6939E-05	4,55531997
TC1500001215.mm.2	18162	Npr3	natriuretic peptide receptor 3	protein-coding	1,02390468	7,8433718	10,0077201	1,1234E-09	4,9375E-08	12,2086291
TC0X00001634.mm.2	110094	Phka2	phosphorylase kinase alpha 2	protein-coding	1,02104045	9,04046615	9,65917932	2,1394E-09	8,4725E-08	11,5559096
TC0400001668.mm.2	71665	Fuca1	fucosidase, alpha-L- 1, tissue	protein-coding	1,02074863	8,05354897	8,33600376	2,8358E-08	6,9369E-07	8,93446729
TC0700000050.mm.2	269855	Ssc5d	scavenger receptor cysteine rich family, 5 domains	protein-coding	1,02066838	6,71746418	9,62530121	2,2794E-09	8,9516E-08	11,4916444
TC0600001083.mm.2	14115	Fbln2	fibulin 2	protein-coding	1,0180853	10,5878759	11,4802738	8,6334E-11	6,7387E-09	14,8036712
TC0600001787.mm.2	209086	Samd9l	sterile alpha motif domain containing 9-like	protein-coding	1,0159869	7,04722533	3,97556076	0,00077864	0,00405063	-1,29895666
TC1500002056.mm.2	11883	Arsa	arylsulfatase A	protein-coding	1,01488594	7,22451446	10,4754588	4,8419E-10	2,4885E-08	13,060837
TC1000000656.mm.2	69288	Rhobtb1	Rho-related BTB domain containing 1	protein-coding	1,01444488	6,77427045	8,1249804	4,3736E-08	9,8885E-07	8,49483216
TC1200000678.mm.2	268564	Zbtb1	zinc finger and BTB domain containing 1	protein-coding	1,01436904	7,74229011	6,47820283	1,9057E-06	2,4075E-05	4,69684375
TC1700000194.mm.2	449000	Zfp960	zinc finger protein 960	protein-coding	1,01263384	7,97131657	7,00413517	5,3593E-07	8,2838E-06	5,9675548
TC0200005094.mm.2	26943	Serinc3	serine incorporator 3	protein-coding	1,01248204	10,7296677	9,06191342	7,6398E-09	2,4235E-07	10,278871
TC0800000088.mm.2	12827	Col4a2	collagen, type IV, alpha 2	protein-coding	1,01188895	7,94630411	11,0350867	1,8281E-10	1,154E-08	14,0459045
TC0500002580.mm.2	21682	Tec	tec protein tyrosine kinase	protein-coding	1,00949672	5,85414369	7,08948811	3,9956E-07	6,4461E-06	6,25111486
TC0600003601.mm.2	108655	Foxp1	forkhead box P1	protein-coding	1,0085391	9,1623456	9,59878673	2,3956E-09	9,2534E-08	11,4412453
TC1000001679.mm.2	64009	Syne1	spectrin repeat containing, nuclear envelope 1	protein-coding	1,00806435	7,75983033	9,3412203	3,9008E-09	1,395E-07	10,9469538
TC0600002648.mm.2	69538	Antxr1	anthrax toxin receptor 1	protein-coding	1,00786964	9,87883623	11,1376993	1,5349E-10	1,0096E-08	14,2225598
TC1100003236.mm.2	76566	Rflnb	refilin B	protein-coding	1,00736463	5,94522679	6,91191696	5,9231E-07	8,9854E-06	5,85223786
TC1200000765.mm.2	54604	Pcnx1	pecanex 1	protein-coding	1,00658903	6,91346141	7,58729342	1,355E-07	2,5825E-06	7,34755964
TC1200001353.mm.2	17977	Ncoa1	nuclear receptor coactivator 1	protein-coding	1,00614138	5,98862487	6,31193655	2,4E-06	2,9449E-05	4,44200355
TC1700001578.mm.2	240041	Zfp945	zinc finger protein 945	protein-coding	1,00425409	5,1035313	6,87575957	6,4206E-07	9,5855E-06	5,77052965
TC1400001054.mm.2	18826	Lcp1	lymphocyte cytosolic protein 1	protein-coding	1,00296107	4,58345238	6,52831668	1,4057E-06	1,8661E-05	4,97719654
TC0700004363.mm.2	67865	Rgs10	regulator of G-protein signalling 10	protein-coding	1,00278504	6,82157647	8,59153548	1,6915E-08	4,5804E-07	9,45880944
TC0800002783.mm.2	12552	Cdh11	cadherin 11	protein-coding	1,002403	8,49876747	8,74115677	1,2548E-08	3,5846E-07	9,76176441
TC1600001607.mm.2	224143	Poglut1	protein O-glucosyltransferase 1	protein-coding	1,00193387	8,75716756	6,13677029	4,8234E-06	5,3122E-05	3,78203852
TC0700004075.mm.2	330627	Trim66	tripartite motif-containing 66	protein-coding	1,0017878	6,14267953	5,43947648	2,2595E-05	0,00019797	2,21554047
TC1000001701.mm.2	215751	Ginm1	glycoprotein integral membrane 1	protein-coding	1,00153335	8,63926601	8,05488562	5,0572E-08	1,1049E-06	8,34747291
TC1000000626.mm.2	108829	Jmjd1c	jumonji domain containing 1C	protein-coding	1,00145889	8,16478174	4,87779784	9,7941E-05	0,00069761	0,77288497
TC1000000147.mm.2	26408	Map3k5	mitogen-activated protein kinase kinase kinase 5	protein-coding	1,00115744	4,69222528	7,75008459	9,5827E-08	1,9075E-06	7,69900458
TC1000001493.mm.2	75612	Gns	glucosamine (N-acetyl)-6-sulfatase	protein-coding	1,00107578	8,92215144	10,9813173	2,0044E-10	1,2444E-08	13,9528524
