# Supplementary material for: ZEB1 integrates stemness, differentiation blockade and immune suppression to orchestrate osteosarcoma aggressiveness
Source: J Exp Clin Cancer Res. 2026 Jun 15;45:183. doi: 10.1186/s13046-026-03757-9 (PMC13495327; doi:10.1186/s13046-026-03757-9)
Supplement: Supplementary file 2 [file 13046_2026_3757_MOESM3_ESM.pdf]

Supplementary table 3B. Differentially down-regulated genes ZEB1KO clones B8 and E4 vs parental and SCR G5P1 contrl; logFC <-1; FDR< 0.05

PROBEID	ENTREZID	SYMBOL	GENENAME	GENETYPE	logFC	AveExpr	t	P.Value	adj.P.Val	B
TC0900001726.mm.2	234915	Cep126	centrosomal protein 126	protein-coding	-4,95023875	7,20320831	-34,7224829	9,1367E-21	4,2968E-17	36,7128526
TC0900000047.mm.2	17392	Mmp3	matrix metallopeptidase 3	protein-coding	-4,47333191	7,54067003	-26,2810126	2,3216E-17	2,569E-14	29,604156
TC0900000044.mm.2	17386	Mmp13	matrix metallopeptidase 13	protein-coding	-4,25168465	7,08502345	-14,9031497	4,2016E-12	6,1269E-10	17,9581983
TC0900000069.mm.2	18667	Pgr	progesterone receptor	protein-coding	-4,16721359	8,22167501	-24,8622304	8,5799E-17	7,6855E-14	28,3890669
TC0X00000801.mm.2	192216	Tmem47	transmembrane protein 47	protein-coding	-3,75109999	6,92435669	-26,1839533	3,8219E-18	5,5303E-15	31,3475665
TC0500000084.mm.2	20346	Sema3a	sema domain, immunoglobulin domain (Ig), short basic domain, secreted, (semaphorin) 3A	protein-coding	-3,59886126	8,12610517	-35,1874623	6,4156E-21	4,0228E-17	37,0208001
TC0900001720.mm.2	11797	Birc2	baculoviral IAP repeat-containing 2	protein-coding	-3,53149567	8,43666496	-29,6010413	2,7233E-19	7,4429E-16	33,7508496
TC0900000040.mm.2	76863	Dcun1d5	defective in cullin neddylation 1 domain containing 5	protein-coding	-3,48001515	10,4099104	-40,9251894	2,3819E-22	4,4015E-18	39,7284105
TC1100001219.mm.2	20296	Ccl2	C-C motif chemokine ligand 2	protein-coding	-3,34630483	9,12978542	-27,7843544	1,0675E-18	2,0081E-15	32,5170531
TC0800000621.mm.2	15446	Hpgd	hydroxyprostaglandin dehydrogenase 15 (NAD)	protein-coding	-3,34581549	6,0491353	-14,3401125	8,376E-12	1,0574E-09	17,2695974
TC0900001724.mm.2	22601	Yap1	yes-associated protein 1	protein-coding	-3,30270728	10,2036145	-39,6790576	4,6797E-22	4,4015E-18	39,1873603
TC0200000382.mm.2	16181	Il1rn	interleukin 1 receptor antagonist	protein-coding	-3,19939108	7,19420771	-12,0138386	1,8785E-10	1,1818E-08	14,143004
TC0900001725.mm.2	234912	Cfap300	cilia and flagella associated protein 300	protein-coding	-3,10662383	9,50380428	-27,4986005	1,3336E-18	2,1452E-15	32,3141625
TC0500000841.mm.2	20311	Cxcl5	C-X-C motif chemokine ligand 5	protein-coding	-3,06814335	7,34955352	-18,9003673	1,0713E-14	4,6863E-12	23,7920621
TC0900001722.mm.2	11796	Birc3	baculoviral IAP repeat-containing 3	protein-coding	-3,00568152	9,4289757	-29,3781825	3,2061E-19	7,5388E-16	33,6045149
TC0400002837.mm.2	56710	Brinp1	bone morphogenic protein/retinoic acid inducible neural specific 1	protein-coding	-2,97296745	6,06841799	-21,0171749	4,1257E-16	3,1044E-13	26,932252
TC0X00000388.mm.2	53623	Gria3	glutamate receptor, ionotropic, AMPA3 (alpha 3)	protein-coding	-2,84321776	4,98119725	-10,3532817	2,3364E-09	9,0995E-08	11,5891459
TC0400000234.mm.2	13841	Epha7	Eph receptor A7	protein-coding	-2,82239321	6,79166534	-19,5017822	1,9917E-15	1,1353E-12	25,4117875
TC1300001377.mm.2	105349	Akr1c18	aldo-keto reductase family 1, member C18	protein-coding	-2,72734681	5,96166688	-10,1097051	3,4649E-09	1,2631E-07	11,1887769
TC0700003943.mm.2	20128	Trim30a	tripartite motif-containing 30A	protein-coding	-2,66013345	5,30749256	-10,483031	1,8991E-09	7,6826E-08	11,7995992
TC0500000856.mm.2	13874	Ereg	epiregulin	protein-coding	-2,57553224	6,84096577	-14,365718	3,3112E-12	5,0639E-10	18,1466624
TC0500000842.mm.2	57349	Ppbp	pro-platelet basic protein	protein-coding	-2,54542965	5,7620538	-8,43640719	6,2694E-08	1,3148E-06	8,24115971
TC1400001157.mm.2	12224	Klf5	Kruppel-like transcription factor 5	protein-coding	-2,4186738	6,13413295	-11,342295	4,1507E-10	2,1932E-08	13,3250298
TC1300000568.mm.2	27047	Omd	osteomodulin	protein-coding	-2,41703555	4,61178603	-7,12408573	7,7995E-07	1,1347E-05	5,67230321
TC0900003338.mm.2	110350	Dync2h1	dynein cytoplasmic 2 heavy chain 1	protein-coding	-2,41633727	6,98878685	-16,8646364	4,1323E-14	1,4395E-11	22,4443308
TC0100003866.mm.2	18788	Serpinb2	serine (or cysteine) peptidase inhibitor, clade B, member 2	protein-coding	-2,38428788	6,51134508	-12,1643234	1,017E-10	7,3683E-09	14,7320978
TC0600002233.mm.2	65963	Tmem176b	transmembrane protein 176B	protein-coding	-2,36946161	5,74013702	-9,54154655	8,9179E-09	2,7411E-07	10,2273407
TC1500002258.mm.2	74127	Krt80	keratin 80	protein-coding	-2,34825661	7,18382297	-15,104658	5,7341E-13	1,3483E-10	19,8547837
TC0900000051.mm.2	234911	Mmp27	matrix metallopeptidase 27	protein-coding	-2,33328834	6,12761584	-12,2707133	6,1589E-11	5,3064E-09	15,2123014
TC0900000055.mm.2	71929	Tmem123	transmembrane protein 123	protein-coding	-2,28150645	11,0046737	-29,5779151	2,7697E-19	7,4429E-16	33,7357272
TC1800000179.mm.2	13527	Dtna	dystrobrevin alpha	protein-coding	-2,27323891	7,17736361	-18,8218364	4,1843E-15	2,1273E-12	24,6896838
TC0300000307.mm.2	18415	Hspa4l	heat shock protein 4 like	protein-coding	-2,26901502	8,05152554	-17,6423643	1,6076E-14	6,574E-12	23,3728654
TC0X00002247.mm.2	22441	Xlr	X-linked lymphocyte-regulated	protein-coding	-2,26701109	5,0384027	-11,1194067	3,536E-10	1,9506E-08	13,4445742
TC1100002052.mm.2	80879	Slc16a3	solute carrier family 16 (monocarboxylic acid transporters), member 3	protein-coding	-2,21799187	9,48591576	-15,312199	8,6629E-13	1,8507E-10	19,4764302
TC0100000298.mm.2	17082	Il1rl1	interleukin 1 receptor-like 1	protein-coding	-2,21796641	9,9446482	-14,767035	2,4949E-12	4,228E-10	18,4417033
TC0200003759.mm.2	108699	Chn1	chimerin 1	protein-coding	-2,16248105	6,58916786	-11,1072275	5,0345E-10	2,5665E-08	13,115188
TC0500000750.mm.2	319387	Adgrl3	adhesion G protein-coupled receptor L3	protein-coding	-2,12909085	3,95975487	-10,6750779	4,4032E-10	2,2944E-08	13,1787258
TC0900000066.mm.2	22068	Trpc6	transient receptor potential cation channel, subfamily C, member 6	protein-coding	-2,11421047	5,81801841	-14,1206708	1,5041E-12	2,7738E-10	18,8736302
TC0100001338.mm.2	19225	Ptgs2	prostaglandin-endoperoxide synthase 2	protein-coding	-2,08882271	8,30971839	-16,5502138	6,0078E-14	1,9639E-11	22,074578
TC1100003396.mm.2	20304	Ccl5	C-C motif chemokine ligand 5	protein-coding	-2,07892327	6,20813288	-8,32006046	7,7677E-08	1,5848E-06	8,02278333
TC1000001936.mm.2	72780	Rspo3	R-spondin 3	protein-coding	-2,07760113	5,01108575	-10,6530743	5,8298E-10	2,8708E-08	12,9155972
TC0100000372.mm.2	20324	Cavin2	caveolae associated 2	protein-coding	-2,03990594	7,56382025	-13,5160289	5,0837E-12	7,1365E-10	17,6749576
TC0300000521.mm.2	16497	Kcnab1	potassium voltage-gated channel, shaker-related subfamily, beta member 1	protein-coding	-2,03246083	7,29535847	-11,2087032	3,7431E-10	2,035E-08	13,4036349
TC1800000646.mm.2	494504	Apcdd1	adenomatosis polyposis coli down-regulated 1	protein-coding	-1,99127681	7,34056722	-10,5464958	1,3263E-09	5,6066E-08	12,1401003
TC1700001671.mm.2	56047	Msln	mesothelin	protein-coding	-1,98723025	6,18130621	-8,5358091	5,2278E-08	1,1369E-06	8,42630622
TC0900002055.mm.2	208098	Panx3	pannexin 3	protein-coding	-1,97740386	6,04675162	-8,7254612	3,7093E-08	8,6403E-07	8,77593307
TC1100001220.mm.2	20306	Ccl7	C-C motif chemokine ligand 7	protein-coding	-1,94295306	9,08962202	-16,5058824	6,348E-14	2,0239E-11	22,0201663
TC1600001414.mm.2	210530	P3h2	prolyl 3-hydroxylase 2	protein-coding	-1,9339764	5,6416974	-8,54671104	4,73E-08	1,0555E-06	8,51890293
TC1200002520.mm.2	67293	3110039M20Rik	RIKEN cDNA 3110039M20 gene	ncRNA	-1,9320186	6,19875983	-13,6077742	3,1446E-12	4,8486E-10	18,1356077
TC1900000226.mm.2	66607	Ms4a4d	membrane-spanning 4-domains, subfamily A, member 4D	protein-coding	-1,91495871	6,04671065	-13,7369405	2,6062E-12	4,302E-10	18,3236944
TC1500001528.mm.2	18383	Tnfrsf11b	tumor necrosis factor receptor superfamily, member 11b (osteoprotegerin)	protein-coding	-1,88973292	6,70239203	-8,51321028	5,4477E-08	1,1752E-06	8,38432856
TC1600001827.mm.2	13837	Epha3	Eph receptor A3	protein-coding	-1,88060327	5,23111601	-5,94397106	9,1602E-06	9,1802E-05	3,16758015
TC0500002751.mm.2	57425	U90926	cDNA sequence U90926	protein-coding	-1,83590126	4,4062192	-4,77137317	0,00012481	0,00085559	0,52923678
TC1000001696.mm.2	77777	Ulbp1	UL16 binding protein 1	protein-coding	-1,80793137	5,56440075	-10,9242743	2,4414E-10	1,4533E-08	13,7616536
TC1300001789.mm.2	66895	Pxdc1	PX domain containing 1	protein-coding	-1,79690857	6,10377349	-12,4210021	1,8922E-11	2,1314E-09	16,333579
TC1600000447.mm.2	22042	Tfrc	transferrin receptor	protein-coding	-1,79149043	9,09568397	-12,5922033	2,1141E-11	2,2825E-09	16,2476327
TC0100001308.mm.2	240776	Kcnt2	potassium channel, subfamily T, member 2	protein-coding	-1,74404803	4,10335792	-9,93868456	1,2748E-09	5,4254E-08	12,0805593
TC0900000049.mm.2	17384	Mmp10	matrix metallopeptidase 10	protein-coding	-1,71878245	4,74511195	-9,01046531	9,017E-09	2,758E-07	10,1175761
TC1600000109.mm.2	68172	Rpl39l	ribosomal protein L39-like	protein-coding	-1,70853626	6,17129693	-7,11849701	7,8876E-07	1,1457E-05	5,66086613
TC1800001197.mm.2	12475	Cd14	CD14 antigen	protein-coding	-1,7056485	6,54602761	-11,4781587	8,777E-11	6,7944E-09	14,7880199
TC0900001018.mm.2	70082	Lysmd2	LysM, putative peptidoglycan-binding, domain containing 2	protein-coding	-1,69383326	5,67784777	-10,7890356	2,7932E-10	1,6167E-08	13,6173455
TC0X00003204.mm.2	75625	Mageh1	MAGE family member H1	protein-coding	-1,65576824	5,34983541	-10,3237333	6,344E-10	3,0997E-08	12,7873313
TC1100003086.mm.2	69309	Slc16a13	solute carrier family 16 (monocarboxylic acid transporters), member 13	protein-coding	-1,648248	6,42304992	-10,4699015	5,7802E-10	2,8573E-08	12,8960711
TC0900001020.mm.2	12160	Bmp5	bone morphogenetic protein 5	protein-coding	-1,64411756	5,13765661	-6,38350448	3,5826E-06	4,1345E-05	4,12090606
TC0700000961.mm.2	17984	Ndn	necdin, MAGE family member	protein-coding	-1,62475577	5,272537	-7,06630703	7,9749E-07	1,1557E-05	5,63637681
TC0900000063.mm.2	76669	1700128F08Rik	U1-snRNP binding protein homolog pseudogene	pseudo	-1,60720018	3,7591053	-5,3256608	3,5685E-05	0,00029288	1,79071993
TC0900001718.mm.2	83996	Mmp1b	matrix metallopeptidase 1b (interstitial collagenase)	protein-coding	-1,60331752	4,2849926	-5,90048153	1,0065E-05	9,9439E-05	3,07203147
TC1900000859.mm.2	107376	E330013P04Rik	RIKEN cDNA E330013P04 gene	ncRNA	-1,5926714	7,76671101	-6,41738624	3,3359E-06	3,888E-05	4,19342302
TC0200001047.mm.2	228026	Pdk1	pyruvate dehydrogenase kinase, isoenzyme 1	protein-coding	-1,58677257	8,19810102	-11,4197151	1,0969E-10	7,7861E-09	14,5727359
TC0100001317.mm.2	56207	Uchl5	ubiquitin carboxyl-terminal esterase L5	protein-coding	-1,58170271	8,37768154	-14,7123325	6,5959E-13	1,5103E-10	19,6965102
TC0900000048.mm.2	83995	Mmp1a	matrix metallopeptidase 1a (interstitial collagenase)	protein-coding	-1,57853249	4,52089776	-5,59437982	1,9657E-05	0,00017658	2,39376137
TC0800002542.mm.2	16168	Il15	interleukin 15	protein-coding	-1,57463737	5,0302264	-9,49525572	2,9113E-09	1,0997E-07	11,2435889
TC1100003397.mm.2	20308	Ccl9	C-C motif chemokine ligand 9	protein-coding	-1,5713005	6,61172142	-8,26667839	6,2222E-08	1,3108E-06	8,21030671
TC1300000262.mm.2	80838	H1f1	H1.1 linker histone, cluster member	protein-coding	-1,5634664	9,68401718	-12,3826802	2,5505E-11	2,5794E-09	16,0495993
TC0100001381.mm.2	19734	Rgs16	regulator of G-protein signaling 16	protein-coding	-1,56029213	6,01293473	-10,729805	3,0965E-10	1,7469E-08	13,5131228
TC1800000060.mm.2	14370	Fzd8	frizzled class receptor 8	protein-coding	-1,55786773	6,92083439	-11,1010892	1,6335E-10	1,0596E-08	14,1596712
TC0400003814.mm.2	11647	Alpl	alkaline phosphatase, liver/bone/kidney	protein-coding	-1,55772819	5,72734645	-8,9027711	1,1092E-08	3,2385E-07	9,90734804
TC0200000819.mm.2	21930	Tnfaip6	tumor necrosis factor alpha induced protein 6	protein-coding	-1,54699325	6,2660677	-10,1458107	8,8352E-10	4,0835E-08	12,4529213
TC0700003625.mm.2	12173	Bnc1	basonuclin zinc finger protein 1	protein-coding	-1,54552001	5,72923489	-10,466092	4,9229E-10	2,5233E-08	13,0440328
TC0200003670.mm.2	14425	Galnt3	polypeptide N-acetylgalactosaminyltransferase 3	protein-coding	-1,53899835	4,30956181	-5,6636866	1,6877E-05	0,00015516	2,5481845
TC0100003193.mm.2	12628	Cfh	complement component factor h	protein-coding	-1,53430553	5,72753313	-9,12220289	6,9437E-09	2,2214E-07	10,3778732
TC0700000383.mm.2	18793	Plaur	plasminogen activator, urokinase receptor	protein-coding	-1,5173378	7,62763457	-11,4261243	9,4476E-11	7,1087E-09	14,7126937
TC0300001064.mm.2	19260	Ptpn22	protein tyrosine phosphatase, non-receptor type 22 (lymphoid)	protein-coding	-1,51603998	4,44043392	-6,94570698	8,183E-07	1,1841E-05	5,578754
TC1800000682.mm.2	66286	Sec11c	SEC11 homolog C, signal peptidase complex subunit	protein-coding	-1,50253367	7,17156503	-10,3546636	6,5814E-10	3,1977E-08	12,7582128
TC1000001244.mm.2	103098	Slc6a15	solute carrier family 6 (neurotransmitter transporter), member 15	protein-coding	-1,48845897	4,40454354	-6,7773815	1,1859E-06	1,6224E-05	5,20378244

TC1800001169.mm.2	107065	Lrrtm2	leucine rich repeat transmembrane neuronal 2	protein-coding	-1,47930571	4,18108579	-6,56872412	1,8759E-06	2,3795E-05	4,73975057
TC0900000050.mm.2	17394	Mmp8	matrix metallopeptidase 8	protein-coding	-1,47830839	4,87484011	-6,35161497	3,4536E-06	3,9979E-05	4,14188184
TC0700001631.mm.2	11717	Ampd3	adenosine monophosphate deaminase 3	protein-coding	-1,47221026	8,04323166	-10,5169293	5,0648E-10	2,575E-08	13,0254903
TC0X00000512.mm.2	70691	3830403N18Rik	RIKEN cDNA 3830403N18 gene	protein-coding	-1,46527389	3,50448113	-7,41870331	2,1883E-07	3,8507E-06	6,87615603
TC1700001938.mm.2	110956	D17H6S56E-5	DNA segment, Chr 17, human D6S56E 5	protein-coding	-1,41620287	8,89397138	-11,7571009	5,4722E-11	4,8555E-09	15,2637205
TC0500001025.mm.2	20750	Spp1	secreted phosphoprotein 1	protein-coding	-1,41345572	10,8509685	-8,90911833	2,6722E-08	6,633E-07	9,11001815
TC0500001023.mm.2	15891	Ibsp	integrin binding sialoprotein	protein-coding	-1,40623839	5,5825517	-5,78627229	1,2904E-05	0,00012322	2,82011789
TC0300000810.mm.2	381493	S100a7a	S100 calcium binding protein A7A	protein-coding	-1,40456887	7,26686189	-7,88949269	1,217E-07	2,3528E-06	7,51994167
TC1300000249.mm.2	319181	H2bc8	H2B clustered histone 8	protein-coding	-1,40041046	8,97635658	-13,5090309	3,6336E-12	5,4681E-10	17,9907922
TC0200002232.mm.2	69270	Gins1	GINS complex subunit 1	protein-coding	-1,39757277	7,97143997	-10,0072385	1,2274E-09	5,2834E-08	12,126973
TC0700001673.mm.2	233752	Insc	INSC spindle orientation adaptor protein	protein-coding	-1,38222481	4,29673542	-8,94858397	8,335E-09	2,5873E-07	10,1768303
TC0100000112.mm.2	94227	Pi15	peptidase inhibitor 15	protein-coding	-1,38085695	6,90725697	-7,99556334	9,3202E-08	1,8632E-06	7,78438042
TC0900001175.mm.2	26432	Plod2	procollagen lysine, 2-oxoglutarate 5-dioxygenase 2	protein-coding	-1,37301521	8,33929693	-13,5246286	3,5514E-12	5,3875E-10	18,0137273
TC1100003141.mm.2	237831	Slc13a5	solute carrier family 13 (sodium-dependent citrate transporter), member 5	protein-coding	-1,37271772	4,37998359	-7,01171198	5,4002E-07	8,3402E-06	5,96308941
TC1900001437.mm.2	107765	Ankrd1	ankyrin repeat domain 1	protein-coding	-1,36788191	7,24932169	-9,49074883	3,1839E-09	1,1907E-07	11,1607057
TC0300000060.mm.2	16592	Fabp5	fatty acid binding protein 5, epidermal	protein-coding	-1,36154104	7,45965087	-11,2468483	1,2761E-10	8,7605E-09	14,4091474
TC1500001809.mm.2	223672	Apol9a	apolipoprotein L 9a	protein-coding	-1,36072761	8,5345279	-9,86190325	1,7118E-09	7,0615E-08	11,7961852
TC0900000046.mm.2	17381	Mmp12	matrix metallopeptidase 12	protein-coding	-1,35753983	5,12091848	-6,86051459	8,9458E-07	1,271E-05	5,47530907
TC0900002915.mm.2	74769	Pik3cb	phosphatidylinositol-4,5-bisphosphate 3-kinase catalytic subunit beta	protein-coding	-1,35221029	8,25112712	-12,3573884	2,0909E-11	2,2736E-09	16,2330602
TC0200004217.mm.2	14221	Fjx1	four jointed box 1	protein-coding	-1,33279642	6,01829992	-8,87234152	9,6813E-09	2,9138E-07	10,024932
TC1100003161.mm.2	11484	Aspa	aspartoacylase	protein-coding	-1,32363013	4,33283929	-8,610591	1,6281E-08	4,4644E-07	9,49755966
TC0200001624.mm.2	12064	Bdnf	brain derived neurotrophic factor	protein-coding	-1,32213008	7,99827791	-9,58371306	2,6755E-09	1,0188E-07	11,3371267
TC1300000972.mm.2	17260	Mef2c	myocyte enhancer factor 2C	protein-coding	-1,31570971	5,48050222	-6,6648831	1,4635E-06	1,9333E-05	4,98576079
TC1300001837.mm.2	66143	Eef1e1	eukaryotic translation elongation factor 1 epsilon 1	protein-coding	-1,31179415	10,1769532	-14,850681	5,461E-13	1,3003E-10	19,8846882
TC0X00002770.mm.2	75106	4930519F16Rik	RIKEN cDNA 4930519F16 gene	protein-coding	-1,30693389	4,21780617	-7,80169037	8,5923E-08	1,7342E-06	7,80967443
TC1900000504.mm.2	15957	Ifit1	interferon-induced protein with tetratricopeptide repeats 1	protein-coding	-1,30203139	6,05875808	-4,8109532	0,00011404	0,00078836	0,61991265
TC0600000025.mm.2	65255	Asb4	ankyrin repeat and SOCS box-containing 4	protein-coding	-1,30151373	5,37736503	-5,53077968	2,2474E-05	0,00019718	2,25704754
TC1800000704.mm.2	67951	Tubb6	tubulin, beta 6 class V	protein-coding	-1,2987863	9,51317441	-13,8713254	2,1468E-12	3,7049E-10	18,5177778
TC0200000821.mm.2	51869	Rif1	replication timing regulatory factor 1	protein-coding	-1,28887819	7,58362384	-9,23341589	4,9489E-09	1,6743E-07	10,7087467
TC1500001201.mm.2	78244	Dnajc21	DnaJ heat shock protein family (Hsp40) member C21	protein-coding	-1,28647551	7,89796409	-9,36627556	3,8403E-09	1,376E-07	10,9659574
TC0X00001700.mm.2	11856	Arhgap6	Rho GTPase activating protein 6	protein-coding	-1,27931608	6,18235218	-8,10209172	4,8826E-08	1,0793E-06	8,39032586
TC0500001931.mm.2	27214	Dbf4	DBF4 zinc finger	protein-coding	-1,27680059	7,86566997	-8,41373238	2,8948E-08	7,0355E-07	8,93332226
TC0500000363.mm.2	107823	Nsd2	nuclear receptor binding SET domain protein 2	protein-coding	-1,27070659	8,7996762	-11,0683307	1,7273E-10	1,1014E-08	14,1032688
TC0100001240.mm.2	67196	Ube2t	ubiquitin-conjugating enzyme E2T	protein-coding	-1,26769767	6,46511928	-9,22191027	4,9029E-09	1,6648E-07	10,715074
TC0500003730.mm.2	236573	Gbp9	guanylate-binding protein 9	protein-coding	-1,2655248	4,99831989	-5,63874122	1,5905E-05	0,00014745	2,58809736
TC0200000274.mm.2	67448	Plxdc2	plexin domain containing 2	protein-coding	-1,26285296	6,08638795	-5,90954305	9,232E-06	9,2325E-05	3,14839615
TC0900000226.mm.2	73230	Bmper	BMP-binding endothelial regulator	protein-coding	-1,25576449	6,92264284	-9,61989011	2,3027E-09	9,0053E-08	11,4813662
TC0900000854.mm.2	68026	Pclaf	PCNA clamp associated factor	protein-coding	-1,25311501	9,12218513	-13,1336453	6,3435E-12	8,4033E-10	17,431962
TC0X00003241.mm.2	211612	Ptchd1	patched domain containing 1	protein-coding	-1,25246564	5,01066643	-5,1553015	5,1989E-05	0,00040597	1,40976221
TC0700000381.mm.2	16534	Kcnn4	potassium intermediate/small conductance calcium-activated channel, subfamily N, member 4	protein-coding	-1,25096047	5,96206946	-9,09049784	6,3202E-09	2,0444E-07	10,457519
TC0700004592.mm.2	79202	Tnfrsf22	tumor necrosis factor receptor superfamily, member 22	protein-coding	-1,24856797	6,61840834	-6,22724621	4,5244E-06	5,0389E-05	3,86813336
TC0200002850.mm.2	64707	Suv39h2	suppressor of variegation 3-9 2	protein-coding	-1,24749674	7,14916641	-8,82804342	1,0575E-08	3,1232E-07	9,93543063
TC0500001066.mm.2	12545	Cdc7	cell division cycle 7	protein-coding	-1,24578813	8,50441796	-11,6863364	6,144E-11	5,3064E-09	15,1469176
TC1100002029.mm.2	56282	Mrpl12	mitochondrial ribosomal protein L12	protein-coding	-1,2450304	8,85228642	-12,3606476	2,0802E-11	2,2736E-09	16,2382203
TC1300001560.mm.2	56702	H1f5	H1.5 linker histone, cluster member	protein-coding	-1,24109945	9,3019191	-12,7381626	1,1566E-11	1,4037E-09	16,8286196
TC0500002394.mm.2	19017	Ppargc1a	peroxisome proliferative activated receptor, gamma, coactivator 1 alpha	protein-coding	-1,24055024	5,62020267	-6,0530278	5,8092E-06	6,2125E-05	3,59368348
TC1900000502.mm.2	667370	Ifit3b	interferon-induced protein with tetratricopeptide repeats 3B	protein-coding	-1,23681409	6,86434807	-4,62662297	0,00017376	0,00113078	0,19710397
TC1400000968.mm.2	20855	Stc1	stanniocalcin 1	protein-coding	-1,23408686	5,33099032	-8,96555007	8,0627E-09	2,5236E-07	10,2105272
TC1500000852.mm.2	76737	Creld2	cysteine-rich with EGF-like domains 2	protein-coding	-1,23202392	9,05792035	-8,28755789	4,7511E-08	1,0589E-06	8,45788881
TC0100001330.mm.2	215378	Brinp3	bone morphogenetic protein/retinoic acid inducible neural specific 3	protein-coding	-1,22999498	4,77625895	-6,08421163	4,9099E-06	5,3917E-05	3,74828713
TC1900000247.mm.2	107373	Fam111a	family with sequence similarity 111, member A	protein-coding	-1,22761277	7,78668706	-10,1251556	9,0723E-10	4,1798E-08	12,4251233
TC0500002814.mm.2	19092	Prkg2	protein kinase, cGMP-dependent, type II	protein-coding	-1,22662987	9,12288363	-6,54328987	2,5625E-06	3,1099E-05	4,46163055
TC1300000599.mm.2	66197	Cks2	CDC28 protein kinase regulatory subunit 2	protein-coding	-1,22641978	6,50645929	-8,57799082	1,738E-08	4,6773E-07	9,43123622
TC1300000191.mm.2	319184	H2bc12	H2B clustered histone 12	protein-coding	-1,21434292	10,2677446	-12,1948532	2,7036E-11	2,6767E-09	15,9743379
TC1300000016.mm.2	105387	Akr1c14	aldo-keto reductase family 1, member C14	protein-coding	-1,21162298	7,69520087	-8,18551186	4,5312E-08	1,0196E-06	8,47713215
TC1400002297.mm.2	18040	Nefm	neurofilament, medium polypeptide	protein-coding	-1,2114796	5,17130058	-9,08836677	6,3464E-09	2,0444E-07	10,4533236
TC0400000085.mm.2	12448	Ccne2	cyclin E2	protein-coding	-1,20976128	6,37011925	-7,78084995	9,5197E-08	1,897E-06	7,71268876
TC0900001101.mm.2	22137	Ttk	Ttk protein kinase	protein-coding	-1,20957799	7,93619883	-8,09010703	5,5569E-08	1,1919E-06	8,27112244
TC1900000501.mm.2	15959	Ifit3	interferon-induced protein with tetratricopeptide repeats 3	protein-coding	-1,20947647	7,31067489	-5,69985707	1,5589E-05	0,00014502	2,62858391
TC1100001586.mm.2	23834	Cdc6	cell division cycle 6	protein-coding	-1,20506162	6,99346756	-9,33440924	3,9518E-09	1,4106E-07	10,9337664
TC1300001635.mm.2	319180	H2bc7	H2B clustered histone 7	protein-coding	-1,20140878	9,94402387	-12,2590212	2,442E-11	2,5102E-09	16,0768067
TC1300001526.mm.2	353346	Gpr141	G protein-coupled receptor 141	protein-coding	-1,20115743	4,3303443	-5,48749876	2,0402E-05	0,00018206	2,32002484
TC0500001914.mm.2	70358	Steap1	six transmembrane epithelial antigen of the prostate 1	protein-coding	-1,20051742	10,1423297	-11,8921947	4,393E-11	4,0311E-09	15,4852008
TC0700004404.mm.2	77590	Chst15	carbohydrate sulfotransferase 15	protein-coding	-1,19899616	5,57178731	-5,97598615	6,7696E-06	7,0589E-05	3,4357209
TC1600000346.mm.2	67775	Rtp4	receptor transporter protein 4	protein-coding	-1,19836839	5,26942764	-5,70976401	1,2546E-05	0,00012016	2,81460662
TC0400004171.mm.2	100038882	Isg15	ISG15 ubiquitin-like modifier	protein-coding	-1,19201088	5,002701	-5,55104474	1,7992E-05	0,00016398	2,4503503
TC1400000940.mm.2	52033	Pbk	PDZ binding kinase	protein-coding	-1,19118886	8,9759025	-7,7388876	1,5563E-07	2,9014E-06	7,26298744
TC0500001927.mm.2	11496	Adam22	a disintegrin and metallopeptidase domain 22	protein-coding	-1,19053981	5,04016802	-9,64110102	2,213E-09	8,7454E-08	11,5216341
TC0500000494.mm.2	54392	Ncapg	non-SMC condensin I complex, subunit G	protein-coding	-1,18532193	7,37629159	-7,67629637	1,3056E-07	2,4958E-06	7,40382154
TC1400001012.mm.2	14586	Gfra2	glial cell line derived neurotrophic factor family receptor alpha 2	protein-coding	-1,18465169	6,14155263	-9,32933029	3,9903E-09	1,4216E-07	10,9239287
TC1300001575.mm.2	319158	H4c9	H4 clustered histone 9	protein-coding	-1,18313581	8,51583254	-9,40809728	3,4342E-09	1,2625E-07	11,0761199
TC0900003116.mm.2	19228	Pth1r	parathyroid hormone 1 receptor	protein-coding	-1,18208553	5,49352702	-6,66583983	1,157E-06	1,5864E-05	5,19065741
TC0X00002845.mm.2	16431	Itm2a	integral membrane protein 2A	protein-coding	-1,18042941	5,02612363	-6,94707237	5,5091E-07	8,4322E-06	5,92641705
TC0600000570.mm.2	66058	Tmem176a	transmembrane protein 176A	protein-coding	-1,18027854	7,0040937	-5,77378967	1,3166E-05	0,00012559	2,79848508
TC0100000022.mm.2	620393	Alkal1	ALK and LTK ligand 1	protein-coding	-1,17430966	3,93720965	-8,07380719	4,8625E-08	1,0761E-06	8,38731668
TC1800001269.mm.2	22240	Dpysl3	dihydropyrimidinase-like 3	protein-coding	-1,17372739	8,59367981	-10,7081782	3,2155E-10	1,8002E-08	13,4749648
TC1800001537.mm.2	75577	Dynap	dynactin associated protein	protein-coding	-1,17309042	5,49491918	-4,58040315	0,00019318	0,00123899	0,09091342
TC0400000149.mm.2	17389	Mmp16	matrix metallopeptidase 16	protein-coding	-1,16798341	6,49710644	-8,81484115	1,0844E-08	3,1724E-07	9,90986336
TC1400001047.mm.2	13885	Esd	esterase D/formylglutathione hydrolase	protein-coding	-1,16759505	10,1631141	-11,8277105	4,8775E-11	4,4324E-09	15,379727
TC1300001631.mm.2	319157	H4c6	H4 clustered histone 6	protein-coding	-1,16500434	10,0975366	-10,709911	3,2058E-10	1,8001E-08	13,4780241
TC0500002183.mm.2	64339	Fndc4	fibronectin type III domain containing 4	protein-coding	-1,1641674	7,2161189	-10,7731897	2,8712E-10	1,6484E-08	13,5895034
TC1500001192.mm.2	27401	Skp2	S-phase kinase-associated protein 2	protein-coding	-1,16081943	8,51749194	-9,24885546	4,6554E-09	1,5922E-07	10,7676045
TC1300001733.mm.2	66694	Uqcrfs1	ubiquinol-cytochrome c reductase, Rieske iron-sulfur polypeptide 1	protein-coding	-1,15987866	8,50419024	-10,8242647	2,6277E-10	1,5441E-08	13,6791391
TC1300000626.mm.2	319719	Simc1	SUMO-interacting motifs containing 1	protein-coding	-1,1541949	6,23341104	-7,91395652	6,7855E-08	1,4073E-06	8,04919701
TC1100001973.mm.2	11799	Birc5	baculoviral IAP repeat-containing 5	protein-coding	-1,15400612	9,11106253	-11,1499652	1,5033E-10	9,9222E-09	14,2435957
TC0900002283.mm.2	244864	Layn	layilin	protein-coding	-1,15329449	8,48105919	-10,1621242	8,4848E-10	3,9409E-08	12,4929219
TC1900000040.mm.2	18563	Pcx	pyruvate carboxylase	protein-coding	-1,1494726	6,49249446	-8,65635426	1,4857E-08	4,1403E-07	9,5904227
TC1300000259.mm.2	319178	H2bc3	H2B clustered histone 3	protein-coding	-1,14839225	9,0067084	-11,3618929	1,0518E-10	7,5228E-09	14,6043534
TC1100000983.mm.2	109212	Pimreg	PICALM interacting mitotic regulator	protein-coding	-1,14714488	7,20434969	-9,409128	3,4275E-09	1,2625E-07	11,0781061

TC1300000244.mm.2	69386	H4c8	H4 clustered histone 8	protein-coding	-1,14436427	10,0824644	-10,59978	3,8879E-10	2,0718E-08	13,2828732
TC0700000946.mm.2	11982	Atp10a	ATPase, class V, type 10A	protein-coding	-1,14378329	7,04021634	-6,79752291	1,0247E-06	1,4321E-05	5,33744913
TC1300001806.mm.2	380840	Lyrm4	LYR motif containing 4	protein-coding	-1,139546	8,74966144	-8,39285421	2,8436E-08	6,9378E-07	8,9448054
TC1800001595.mm.2	16891	Lipg	lipase, endothelial	protein-coding	-1,13796275	6,34374938	-7,97139753	6,0174E-08	1,2776E-06	8,17109349
TC1300000161.mm.2	19720	Trim27	tripartite motif-containing 27	protein-coding	-1,13535222	7,69782851	-8,30614015	3,0141E-08	7,2596E-07	8,87261533
TC0X00003360.mm.2	170743	Tlr7	toll-like receptor 7	protein-coding	-1,13274339	4,87476794	-6,58392589	1,2452E-06	1,6974E-05	5,10059461
TC0900001038.mm.2	14629	Gclc	glutamate-cysteine ligase, catalytic subunit	protein-coding	-1,12003405	8,57314993	-11,6394568	6,6358E-11	5,5478E-09	15,0692383
TC1300002037.mm.2	74325	Cltb	clathrin light chain B	protein-coding	-1,11692201	7,24227715	-11,0897795	1,6652E-10	1,0734E-08	14,1402125
TC1900001464.mm.2	19662	Rbp4	retinol binding protein 4, plasma	protein-coding	-1,11517227	6,11690657	-8,02770993	5,3511E-08	1,157E-06	8,2901638
TC1300000192.mm.2	319183	H2bc11	H2B clustered histone 11	protein-coding	-1,11450798	10,2253583	-11,0411352	1,8093E-10	1,146E-08	14,0563509
TC0800000690.mm.2	18166	Npy1r	neuropeptide Y receptor Y1	protein-coding	-1,11090612	3,57647277	-6,69957544	9,5348E-07	1,3465E-05	5,3700705
TC1300002197.mm.2	238673	Zfp367	zinc finger protein 367	protein-coding	-1,10792413	8,15237677	-9,18674036	5,2465E-09	1,753E-07	10,6463665
TC1300000491.mm.2	70078	Nol7	nucleolar protein 7	protein-coding	-1,10724058	8,13028234	-8,59178609	1,6906E-08	4,5804E-07	9,45931937
TC0100002308.mm.2	17912	Myo1b	myosin IB	protein-coding	-1,10700786	7,54886353	-8,0277536	5,3506E-08	1,157E-06	8,29025597
TC0600002773.mm.2	16206	Lrig1	leucine-rich repeats and immunoglobulin-like domains 1	protein-coding	-1,10217798	7,55594266	-10,6238308	3,7271E-10	2,0322E-08	13,325614
TC0200002478.mm.2	17865	Mybl2	myeloblastosis oncogene-like 2	protein-coding	-1,10021687	7,08019134	-8,30458618	3,0236E-08	7,2734E-07	8,86939352
TC1500000240.mm.2	67724	Pop1	processing of precursor 1, ribonuclease P/MRP family, (S. cerevisiae)	protein-coding	-1,09760501	8,09838519	-9,16674761	5,4529E-09	1,8091E-07	10,6072366
TC0500003271.mm.2	20778	Scarb1	scavenger receptor class B, member 1	protein-coding	-1,0964265	8,08249688	-11,3956263	9,9409E-11	7,3046E-09	14,6613097
TC0200004219.mm.2	12505	Cd44	CD44 antigen	protein-coding	-1,0963145	10,1208444	-9,73874941	1,8444E-09	7,5098E-08	11,706275
TC0500002827.mm.2	11991	Hnrnpd	heterogeneous nuclear ribonucleoprotein D	protein-coding	-1,09510343	9,7349245	-9,71702501	1,9205E-09	7,7194E-08	11,6653018
TC0200000391.mm.2	67187	Zmynd19	zinc finger, MYND domain containing 19	protein-coding	-1,09160844	7,76503222	-10,679172	3,3827E-10	1,877E-08	13,4236999
TC1300002098.mm.2	70153	Qng1	Q-nucleotide N-glycosylase 1	protein-coding	-1,08772266	7,67174906	-10,1147764	9,2447E-10	4,2312E-08	12,4060581
TC0500001911.mm.2	207686	Cfap69	cilia and flagella associated protein 69	protein-coding	-1,08464822	5,99719842	-6,27566111	2,7837E-06	3,3374E-05	4,30178296
TC0900000508.mm.2	12282	Hyou1	hypoxia up-regulated 1	protein-coding	-1,08413311	9,89645453	-8,94522841	9,1585E-09	2,7922E-07	10,0903117
TC0800000937.mm.2	66929	Asf1b	anti-silencing function 1B histone chaperone	protein-coding	-1,08365109	9,37622274	-11,3811543	1,0184E-10	7,3683E-09	14,6368905
TC1100001899.mm.2	445007	Nup85	nucleoporin 85	protein-coding	-1,08166505	7,33389662	-8,19077871	3,8185E-08	8,8243E-07	8,63255659
TC1500000543.mm.2	223626	Them6	thioesterase superfamily member 6	protein-coding	-1,08020025	6,99950775	-9,68388501	2,0429E-09	8,1418E-08	11,6026828
TC0300001528.mm.2	51886	Fubp1	far upstream element (FUSE) binding protein 1	protein-coding	-1,07890513	10,0856599	-10,9773563	2,0181E-10	1,2488E-08	13,9459843
TC0X00000126.mm.2	21912	Tspan7	tetraspanin 7	protein-coding	-1,07632385	6,35387233	-8,17212327	3,9681E-08	9,1029E-07	8,59356758
TC1300002036.mm.2	28126	Nop16	NOP16 nucleolar protein	protein-coding	-1,07515967	9,30109038	-11,5590228	7,577E-11	6,1436E-09	14,9353984
TC1800000890.mm.2	109672	Cyb5a	cytochrome b5 type A (microsomal)	protein-coding	-1,07314776	10,1105566	-11,8512541	4,6945E-11	4,2868E-09	15,4182879
TC0100000055.mm.2	109294	Prex2	phosphatidylinositol-3,4,5-trisphosphate-dependent Rac exchange factor 2	protein-coding	-1,07237595	4,86511567	-6,83227082	7,0763E-07	1,0416E-05	5,67203789
TC0200002645.mm.2	109054	Pfdn4	prefoldin 4	protein-coding	-1,07204178	8,25877825	-7,71125533	1,1334E-07	2,2154E-06	7,5390835
TC1300000359.mm.2	78903	Wrnip1	Werner helicase interacting protein 1	protein-coding	-1,06854083	6,92966723	-10,572766	4,0772E-10	2,1665E-08	13,2347841
TC0500001909.mm.2	64945	Cldn12	claudin 12	protein-coding	-1,06634229	8,25362034	-12,7556471	1,126E-11	1,3754E-09	16,8556174
TC0900000819.mm.2	66131	Tipin	timeless interacting protein	protein-coding	-1,06544405	7,73329669	-8,04917003	5,1176E-08	1,1155E-06	8,33542792
TC0100003599.mm.2	26388	Ifi202b	interferon activated gene 202B	protein-coding	-1,06414726	8,20278287	-7,48277555	1,9093E-07	3,4567E-06	7,01456489
TC0500000298.mm.2	12615	Cenpa	centromere protein A	protein-coding	-1,06343198	9,58087461	-11,2376035	1,2961E-10	8,8659E-09	14,3933962
TC0400001274.mm.2	108067	Eif2b3	eukaryotic translation initiation factor 2B, subunit 3	protein-coding	-1,05713234	7,70741862	-8,98666215	7,7366E-09	2,4501E-07	10,2524054
TC0700004002.mm.2	74558	Gvin1	GTPase, very large interferon inducible 1	protein-coding	-1,05497285	4,24060635	-5,95406271	5,3016E-06	5,7684E-05	3,63563731
TC0300001031.mm.2	18049	Ngf	nerve growth factor	protein-coding	-1,05296117	8,31177632	-6,60307054	1,6728E-06	2,1612E-05	4,85013285
TC1200000142.mm.2	18263	Odc1	ornithine decarboxylase, structural 1	protein-coding	-1,05037075	8,66607843	-10,1037168	9,4321E-10	4,3065E-08	12,3857283
TC1100004127.mm.2	107817	Jmjd6	jumonji domain containing 6	protein-coding	-1,05030838	7,71279892	-9,29091149	4,2946E-09	1,5E-07	10,8494045
TC0900001908.mm.2	68743	Anln	anillin, actin binding protein	protein-coding	-1,04904131	8,37889795	-8,49953441	2,0353E-08	5,3101E-07	9,27103662
TC1100003693.mm.2	21973	Top2a	topoisomerase (DNA) II alpha	protein-coding	-1,04896417	8,57207869	-10,1228489	9,1103E-10	4,1798E-08	12,4208874
TC0200000354.mm.2	56075	Pdss1	prenyl (solanesyl) diphosphate synthase, subunit 1	protein-coding	-1,04848006	6,05579709	-5,8541374	8,0767E-06	8,2081E-05	3,24097894
TC0900000833.mm.2	214424	Parp16	poly (ADP-ribose) polymerase family, member 16	protein-coding	-1,04838	5,87797104	-7,75823141	9,4189E-08	1,8809E-06	7,71649935
TC0400002016.mm.2	70025	Acot7	acyl-CoA thioesterase 7	protein-coding	-1,04605749	8,62533808	-10,1758657	8,2766E-10	3,8729E-08	12,5180802
TC0700002173.mm.2	77891	Ube2s	ubiquitin-conjugating enzyme E2S	protein-coding	-1,04363189	9,00735408	-9,28745934	4,3231E-09	1,5013E-07	10,8426987
TC1300000247.mm.2	260423	H3c7	H3 clustered histone 7	protein-coding	-1,04162923	10,0044363	-9,15007221	5,6315E-09	1,8585E-07	10,5745594
TC1300001342.mm.2	14165	Fgf10	fibroblast growth factor 10	protein-coding	-1,04156031	5,92370617	-5,22439393	3,8098E-05	0,00031078	1,6938344
TC0X00000096.mm.2	102941	B630019K06Rik	F-box and leucine-rich repeat protein 17 pseudogene	pseudo	-1,04153679	5,9227153	-7,22713917	2,9532E-07	4,9704E-06	6,55754077
TC1300000101.mm.2	19166	Psma2	proteasome subunit alpha 2	protein-coding	-1,04098171	9,43153172	-9,98869035	1,1632E-09	5,0301E-08	12,1733859
TC0100003478.mm.2	80914	Uck2	uridine-cytidine kinase 2	protein-coding	-1,0406788	9,6693921	-9,49384512	2,9191E-09	1,1004E-07	11,2408863
TC1300000177.mm.2	319187	H2bc15	H2B clustered histone 15	protein-coding	-1,0403825	10,5239603	-11,9632353	3,9167E-11	3,6751E-09	15,6008832
TC0500000080.mm.2	108151	Sema3d	sema domain, immunoglobulin domain (Ig), short basic domain, secreted, (semaphorin) 3D	protein-coding	-1,03815514	7,11005108	-4,19958138	0,00046416	0,00260872	-0,78488514
TC1100000859.mm.2	14457	Gas7	growth arrest specific 7	protein-coding	-1,03804535	5,75051371	-6,08157611	4,2933E-06	4,8187E-05	3,86201987
TC0300001757.mm.2	13605	Ect2	ect2 oncogene	protein-coding	-1,03755216	9,18408843	-9,68612651	2,0344E-09	8,125E-08	11,6069225
TC1100003470.mm.2	270906	Prr11	proline rich 11	protein-coding	-1,03734554	6,57184571	-7,42719992	1,9117E-07	3,4578E-06	6,99849726
TC1600000700.mm.2	224171	Cip2a	cell proliferation regulating inhibitor of protein phosphatase 2A	protein-coding	-1,03666023	8,09216949	-6,65032116	1,3631E-06	1,8212E-05	5,0430148
TC0900000876.mm.2	67941	Rps27l	ribosomal protein S27-like	protein-coding	-1,03475308	9,27619624	-12,831815	1,002E-11	1,2319E-09	16,9728757
TC1300001630.mm.2	319182	H2bc9	H2B clustered histone 9	protein-coding	-1,02995252	10,1467214	-11,7058542	5,9506E-11	5,2306E-09	15,1791878
TC0100001882.mm.2	12490	Cd34	CD34 antigen	protein-coding	-1,0296514	10,1763547	-11,3110145	1,1454E-10	8,07E-09	14,5182076
TC0600000047.mm.2	94192	C1galt1	core 1 synthase, glycoprotein-N-acetylgalactosamine 3-beta-galactosyltransferase, 1	protein-coding	-1,02944722	7,63940374	-7,14401847	3,849E-07	6,2525E-06	6,29980197
TC1300001555.mm.2	319185	H2bc13	H2B clustered histone 13	protein-coding	-1,02759751	10,3138804	-10,8816214	2,3795E-10	1,4301E-08	13,7794343
TC0200001760.mm.2	19361	Rad51	RAD51 recombinase	protein-coding	-1,02655811	8,31171646	-9,737217	1,8497E-09	7,515E-08	11,7033868
TC0200005271.mm.2	20878	Aurka	aurora kinase A	protein-coding	-1,02597098	8,44572756	-10,2273165	7,5429E-10	3,5831E-08	12,6120708
TC1200000951.mm.2	399558	Flrt2	fibronectin leucine rich transmembrane protein 2	protein-coding	-1,0246788	7,23398183	-6,04009042	5,778E-06	6,1896E-05	3,5936491
TC0200001620.mm.2	228421	Kif18a	kinesin family member 18A	protein-coding	-1,02251819	6,79836804	-6,72973382	9,5285E-07	1,3465E-05	5,38008201
TC0800001654.mm.2	13642	Efnb2	ephrin B2	protein-coding	-1,0220898	9,10202312	-8,45290834	2,2364E-08	5,7003E-07	9,17543985
TC1800001700.mm.2	110796	Tshz1	teashirt zinc finger family member 1	protein-coding	-1,02148006	7,01569874	-8,79526543	1,1272E-08	3,2773E-07	9,87058847
TC1100000964.mm.2	13808	Eno3	enolase 3, beta muscle	protein-coding	-1,01547813	8,77237779	-9,0149277	7,3213E-09	2,3342E-07	10,3083811
TC0500002046.mm.2	433855	AI506816	expressed sequence AI506816	ncRNA	-1,01512679	8,41369514	-8,28326636	3,1584E-08	7,53E-07	8,8251591
TC1300002392.mm.2	13003	Vcan	versican	protein-coding	-1,01506448	9,1458797	-6,47231548	2,2882E-06	2,8281E-05	4,53664933
TC0900001883.mm.2	67629	Spc24	SPC24, NDC80 kinetochore complex component, homolog (S. cerevisiae)	protein-coding	-1,01310167	7,62004791	-7,47436483	1,7268E-07	3,1628E-06	7,10168702
TC0900002608.mm.2	68178	Cgnl1	cingulin-like 1	protein-coding	-1,01084686	8,28125893	-10,1991143	7,9363E-10	3,751E-08	12,5605912
TC1100003551.mm.2	217109	Utp18	UTP18 small subunit processome component	protein-coding	-1,00962363	9,06569076	-9,58832555	2,4431E-09	9,3984E-08	11,4213358
TC1300000175.mm.2	319186	H2bc14	H2B clustered histone 14	protein-coding	-1,00797993	10,3586426	-10,2789706	6,874E-10	3,3155E-08	12,7061059
TC1300001634.mm.2	319166	H2ac8	H2A clustered histone 8	protein-coding	-1,007971	11,0789257	-11,2014303	1,3778E-10	9,2687E-09	14,3316709
TC1300001171.mm.2	60411	Cenpk	centromere protein K	protein-coding	-1,00658214	6,12109257	-8,36312148	2,6834E-08	6,6486E-07	8,99052813
TC0300002035.mm.2	18645	Pfn2	profilin 2	protein-coding	-1,00642433	7,99649953	-8,50493356	2,0133E-08	5,2746E-07	9,28208757
TC0X00002631.mm.2	18968	Pola1	polymerase (DNA directed), alpha 1	protein-coding	-1,00470245	7,60403041	-8,88366832	9,4679E-09	2,8587E-07	10,0475472
TC0400001135.mm.2	16975	Lrp8	low density lipoprotein receptor-related protein 8, apolipoprotein e receptor	protein-coding	-1,0040654	6,95541019	-6,71564609	9,8045E-07	1,3784E-05	5,3507538
TC0300001446.mm.2	55932	Gbp3	guanylate binding protein 3	protein-coding	-1,00179829	5,56808601	-5,06703087	5,3604E-05	0,0004165	1,34569523
