# Supplementary material for: ZEB1 integrates stemness, differentiation blockade and immune suppression to orchestrate osteosarcoma aggressiveness
Source: J Exp Clin Cancer Res. 2026 Jun 15;45:183. doi: 10.1186/s13046-026-03757-9 (PMC13495327; doi:10.1186/s13046-026-03757-9)
Supplement: Supplementary file 3 [file 13046_2026_3757_MOESM2_ESM.docx]

| **PROBE** | **SOURCE** | **IDENTIFIER** |
| --- | --- | --- |
| Gapdh | Thermo Fisher Scientific | Mm99999915_g1 |
| Zeb1 | Thermo Fisher Scientific | Mm00495564_m1 |
| Nanog | Thermo Fisher Scientific | Mm02019550_s1 |
| Sox2 | Thermo Fisher Scientific | Mm03053810_s1 |
| Pou5f1 | Thermo Fisher Scientific | Mm03053917_g1 |
| Runx2 | Thermo Fisher Scientific | Mm00501584_m1 |
| Sp7 | Thermo Fisher Scientific | Mm00504574_m1 |
| Bglap | Thermo Fisher Scientific | Mm03413826_mH |
| Alp | Thermo Fisher Scientific | Mm00475834_m1 |
| Col1A1 | Thermo Fisher Scientific | Mm00801666_g1 |
| Sfrp1 | Thermo Fisher Scientific | Mm00489161_m1 |
| Ccl2 | Integrated DNA Technologies | Mm.PT.58.42151692 |
| Cxcl5 | Integrated DNA Technologies | Mm.PT.58.29518961.g |
| Yap1 | Integrated DNA Technologies | Mm.PT.58.6477669 |
| Mmp3 | Integrated DNA Technologies | Mm.PT.58.9719290 |
| Mmp13 | Integrated DNA Technologies | Mm.PT.58.42286812 |

**Supplementary Table S2. Probes for RT-PCR**
