# Supplementary material for: ZEB1 integrates stemness, differentiation blockade and immune suppression to orchestrate osteosarcoma aggressiveness
Source: J Exp Clin Cancer Res. 2026 Jun 15;45:183. doi: 10.1186/s13046-026-03757-9 (PMC13495327; doi:10.1186/s13046-026-03757-9)
Supplement: Supplementary file 4 [file 13046_2026_3757_MOESM4_ESM.docx]

| **ANTIBODY** | **COMPANY** | **DILUTION** |
| --- | --- | --- |
| ZEB1 | Cell Signaling, E2G6Y Ref. 70512 | 1:1000 |
| Beta-Actin | Sigma, Ref. A5316 | 1:1000 |
| Goat anti-rabbit IgG HRP | Thermo Fisher Scientific Ref. 31460 | 1:5000 |
| Goat anti-mouse IgG HRP | Thermo Fisher Scientific Ref. 31430 | 1:10000 |

**Supplementary Table S1A. Antibodies for Western-Blot**

| **REAGENT** | **SOURCE** | **IDENTIFIER** |
| --- | --- | --- |
| **Antibodies** | | |
| Rat monoclonal anti-mouse CD206 (clone: C068C2) | BioLegend | Cat# 141704, RRID:AB_10901166 |
| Rat monoclonal anti-mouse CD11b (clone: M1/70) | TONBO Biosciences | Cat# 50-0112, RRID:AB_2621746 |
| Rat monoclonal anti-mouse CD45 (clone: 30-F11) | BD Biosciences | Cat# 563891, RRID:AB_2734134) |
| Rat monoclonal anti-mouse F4/80 (clone: BM8) | Thermo Fisher Scientific | Cat# 25-4801-82, RRID:AB_469653 |
| Rat monoclonal anti-mouse Ly-6G/Ly-6C (clone: RB6-8C5) | eBioscience | Cat# 14-5931-82  RRID: AB_467730 |
| Rat monoclonal anti-mouse Ly-6C (clone: AL-21) | BD Biosciences | Cat# 562727, RRID:AB_2737748 |
| Hamster monoclonal anti-mouse CD11c (clone HL-3) | BD Biosciences | Cat# 560584  RRID:AB_1727422 |
| Hamster monoclonal anti-mouse CD3e (clone: 145-2C11) | eBioscience | Cat# 45-0031-82  RRID: AB_1107000 |
| Rat monoclonal anti-mouse CD8a (clone: 2.43) | TONBO Biosciences | Cat# 50-0081, RRID:AB_2621741 |
| Rat monoclonal anti-mouse PD-L1 (CD274) (clone: MIH5) | BD Biosciences | Cat# 741014, RRID:AB_2740636 |
| Mouse monoclonal anti mouse Tbet (clone 4B10) | BD Biosciences | Cat# 561265  RRID:AB_10565980 |
| Mouse monoclonal anti-human/mouse GZMB (clone: QA16A02) | BioLegend | Cat# 372212  RRID:AB_2728379 |
| Rat monoclonal anti-mouse CD4 (clone: RM4-5) | BD Biosciences | Cat# 563747, RRID:AB_2716859 |
| Hamster monoclonal anti-mouse PD-1 (CD279) (clone: J43) | BD Biosciences | Cat# 744548, RRID:AB_2742319 |
| Rat monoclonal anti-mouse Ki67 (clone: SolA15) | Thermo Fisher Scientific | Cat# 17-5698-82, RRID:AB_2688057 |
| Rat monoclonal anti-mouse FOXP3 (clone: R16-715) | BD Biosciences | Cat# 563902, RRID:AB_2630318 |
| Rat monoclonal anti-mouse CD25 (clone: 3C7) | BioLegend | Cat# 101908, RRID:AB_961212 |
| Hamster monoclonal anti-mouse CTLA-4 (clone UC10-4B9) | BioLegend | Cat# 106311  RRID: AB_10901170 |
| Rat monoclonal anti-mouse OX40 (clone OX-86) | eBioscience | Cat# 12-1341-82  RRID: AB_465854 |
| Mouse monoclonal anti-mouse TIM3 (clone SD-12) | BD Biosciences | Cat# 747626  RRID: AB_2744192 |

**Supplementary Table S1B. Antibodies for flow cytometry**
