# Supplementary material for: ZEB1 integrates stemness, differentiation blockade and immune suppression to orchestrate osteosarcoma aggressiveness
Source: J Exp Clin Cancer Res. 2026 Jun 15;45:183. doi: 10.1186/s13046-026-03757-9 (PMC13495327; doi:10.1186/s13046-026-03757-9)
Supplement: Supplementary file 5 [file 13046_2026_3757_MOESM5_ESM.docx]

***Supplementary figures***

***Supplementary figure S1***

***Supplementary Figure S1. Efficacy of ZEB1 deletion in mOS69 clones and in vitro proliferation of ZEB1KO clones and control***

A. Screening by Western blot of ZEB1 expression in different mOS69 single cell clones. B. Western blot of ZEB1 expression on selected mOS69 ZEB1 KO clones B8, E4 and H3 and SCR G5p1 control clone. Expression of house-keeping b-actin was use as reference. C. In vitro proliferation of ZEB1Ko clones, control clone and parental mOS69 cell line. Cell Titer Glow assay has been used. Three technical replicates for each line. Ratio to T0 is shown.

***Supplementary Figure S2***

***Supplementary Figure S2.*** ***ZEB1 inhibition by shRNA interference reduced aggressive traits also in mOS5 cell line.*** A. Western blot analysis of ZEB1 expression in mOS5 cell line silenced for Zeb1 (mOS5 KD) by shRNA interference. B. B. Densitometric measurement of bands intensity from western blot in panel A. C. RT-PCR analysis for stemness-related genes, Nanog, Pou5f1 and Sox2 on mOS5 cell lines silenced for Zeb1 and control shGFP line. D. Soft agar assay in mOS5 KD cells and controls. Experiment performed once with two biological replicates. Statistical analysis used: one-way ANOVA. E. Representative images of the spheroids formed in soft agar assay. F. In vivo tumor growth of mOS5 KD and control lines inkjected subcutaneously in immune comeptent BALB/c mice. Five mice per group are used. Statistical analysis used: is two-ways ANOVA.

***Supplementary Figure S3***

***Supplementary Figure S3. Tumor size of ZEB1KO tumors.*** Representative images of tumor lesions from experiment shown in Figure 3. As mice injected with ZEB1KO B8 clone did not develop tumors at the time of sacrifice, no images are shown for this line.

***Supplementary Figure S4***

***Supplementary Figure S4. Gating strategies formultiparamentric flow cytometry analysis of tumor immune infiltrates.*** A**.** Gating strategy for myeloid cells (total CD11b on CD45+ gated cells, F480 + macrophages, CD206 and PD-L1 expression on F480+ macrophages, Gr1high granulocytic cells, Ly6Chigh monocytic cells and PD-L1 expression on them. B. CD8 T lymphocytes gating strategy. On CD45+ cells CD3+CD8+ are gated and then the expression of Ki67 as proliferation marker is defined on CD3+CD8+ cells, as well as PD-1 and Tbet expression on the same subset. C. CD4 T lymphocytes gating strategy. On CD45+ cells CD4+ are gated and then FOXP3 expression on CD4+T cells to divide FOXP3+ T regularoty cells (Tregs) and FOXP3- T conventional cells (Tconv). An example of two different markers (ki67 and PD1) is shown on Tregs and Tconv. D. Flow cytometry analysis of CD4 T cells infiltrating subcutaneous tumors for the experiment shown in Figure 3.

***Supplementary Figure S5***

***Supplementary Figure S5. Inhibition of ZEB1 expression in a PDX derived cell line efficiently impairs OS aggressive traits by reducing stemness potential and activating osteogenic differentiation.*** A. Western blot analysis evaluating the protein expression of ZEB1 in PDX-OS19 and its inhibition with short hairpin sh545 and sh631. B. Densitometric measurement of bands intensity from western blot in panel C. Sarcosphere forming assay on PDX-OS19 shZeb1 545 and 631 and shSCR scramble control lines. Experiment performed once with three replicates. D. Representative images of the spheroids formed. For the analysis of the stemness potential the statistical analysis used is the one-way ANOVA. E. Alkaline phosphatase staining at 48 hr on PDX19 shZEB1 and control lines and after 5 days. Side bar 50um.
